# B cell receptor-induced protein dynamics and the emerging role of SUMOylation revealed by proximity proteomics

**DOI:** 10.1242/jcs.261119

**Published:** 2023-08-08

**Authors:** Luqman O. Awoniyi, Diogo M. Cunha, Alexey V. Sarapulov, Sara Hernández-Pérez, Marika Runsala, Blanca Tejeda-González, Vid Šuštar, M. Özge Balci, Petar Petrov, Pieta K. Mattila

**Affiliations:** ^1^ Institute of Biomedicine and MediCity Research Laboratories, University of Turku, 20014 Turku, Finland; ^2^ Turku Bioscience, University of Turku and Åbo Akademi University, 20520 Turku, Finland; ^3^ InFLAMES Research Flagship Center, University of Turku, 20014 Turku, Finland

**Keywords:** B cells, BCR signaling, Lipid rafts, APEX2, SUMOylation, Golga3

## Abstract

Successful B cell activation, which is critical for high-affinity antibody production, is controlled by the B cell antigen receptor (BCR). However, we still lack a comprehensive protein-level view of the very dynamic multi-branched cellular events triggered by antigen binding. Here, we employed APEX2 proximity biotinylation to study antigen-induced changes, 5–15 min after receptor activation, at the vicinity of the plasma membrane lipid rafts, wherein BCR enriches upon activation. The data reveals dynamics of signaling proteins, as well as various players linked to the subsequent processes, such as actin cytoskeleton remodeling and endocytosis. Interestingly, our differential expression analysis identified dynamic responses in various proteins previously not linked to early B cell activation. We demonstrate active SUMOylation at the sites of BCR activation in various conditions and report its functional role in BCR signaling through the AKT and ERK1/2 axes.

## Teaser

**Summary:** By utilizing proximity labeling of proteins in the lipid raft domains of the plasma membrane, this work reveals the molecular environment and dynamics triggered by the B cell receptor upon antigen activation.

## INTRODUCTION

B lymphocytes constitute a critical branch of the adaptive immune system because they differentiate into antibody-producing plasma cells after the specific recognition of antigens via their distinctive B cell receptor (BCR). BCR signaling is a robust trigger that leads to phosphorylation of downstream kinases and cellular structural processes, like actin cytoskeleton reorganization and internalization of the BCR, within minutes of activation. Numerous studies on BCR signaling have sketched a picture of a multibranched signaling network that not only triggers cascades to change transcriptional programming but also to alter other cellular machineries, such as cytoskeleton reorganization, endocytosis and vesicle transport, as well as protein degradation (Harwood and Batista, 2010; Kuokkanen et al., 2015; Kwak et al., 2019). Engagement of such a wide variety of, often interlinked, cellular pathways has challenged our understanding of the early events of B cell activation and raised a need for broader approaches, yet with sufficient spatial and temporal resolution.

An efficient way to analyze the signaling networks and identify novel players in given cellular pathways is proteomics. Quantitative mass spectrometry (MS)-based proteomics has previously been employed to study BCR signaling. For instance, Satpathy and colleagues have used affinity purification to study the dynamics of BCR interactions upon receptor stimulation (Satpathy et al., 2015). Notably, the sample preparation for traditional proteomic approaches, relying on co-immunoprecipitation or organelle purification, occurs in the test tube and, thus, poses significant challenges in capturing weak or transient protein interactions (Qin et al., 2021). In recent years, proximity labeling techniques have become a powerful tool for mapping protein–protein interactions in the native cellular environment. These techniques include antibody-based approaches, such as enzyme-mediated activation of radical sources (EMARS) and selective proteomic proximity labeling using tyramide (SPPLAT), and use of the promiscuous biotin ligases BioID/BirA*, the engineered ascorbate peroxidases APEX and the variants thereof (Bosch et al., 2021; Qin et al., 2021; Samavarchi-Tehrani et al., 2020). Proximity-based techniques are based on biotinylation triggered by enzymes that are tagged to a compartment or protein of interest and generate short-lived biotin radicals marking their immediate molecular environment in living cells.

SPPLAT has been successfully applied to the study of BCR interactions, using anti-IgM horseradish peroxidase (HRP)-conjugated antibodies to capture the proteins proximal to the BCR clusters in the chicken B cell line DT40 (Li et al., 2014). However, because it relies on HRP-conjugated antibodies, the biotinylation in SPPLAT is restricted mainly to the extracellular side of the plasma membrane, failing to capture the complexity of processes triggered inside the cell. BioID and APEX2, on the other hand, have a biotinylation radius of 10–20 nm, and they have been successfully used to identify immediate protein environments in various intracellular compartments, such as the mitochondrial matrix, mitochondrial intermembrane space and primary cilia (Bareja et al., 2018; Bosch et al., 2021; Hung et al., 2014; Mick et al., 2015; Rhee et al., 2013; Roux et al., 2012). The second-generation version of APEX, APEX2, which achieves efficient biotinylation in 1 min, is the fastest and most efficient labeling enzyme to date (Hung et al., 2016; Lam et al., 2015). As a comparison, TurboID, the fastest member of the BioID family, requires 5–10 min (Chen and Perrimon, 2017; Doerr, 2018). Therefore, the fast-labeling kinetics enabled by APEX2 makes it powerful in capturing dynamic signaling events. For example, APEX2 was recently used in a tour de force of tracking GPCR signaling and internalization with a high spatial resolution (Paek et al., 2017). As for any other fusion protein, expression of APEX2 as a fusion partner of a particular signaling protein can prove technically challenging and potentially compromise the protein function. In such a scenario, targeting the enzyme to the cellular compartment of interest could provide a better readout.

Upon antigen binding, BCR is known to shift from fluid, detergent-soluble plasma membrane domains to less fluid, detergent-resistant cholesterol- and sphingolipid-rich membrane domains, called lipid rafts, for signaling and internalization (Aman and Ravichandran, 2000; Cheng et al., 1999; Gupta et al., 2006; Sohn et al., 2006; 2008; Stone et al., 2017; Varshney et al., 2016). In the past, B cell lipid rafts have been isolated for proteomic analysis via cell fractionation methods (Gupta et al., 2006; Mielenz et al., 2005; Saeki et al., 2003). However, the challenging nature of the biochemical fractionation is illustrated by the limited protein identification, with reported protein numbers in these studies varying between 18 and 39.

Here, we pioneer the use of APEX2 for tracking the cellular events occurring at the vicinity of the B cell plasma membrane after activation of the IgM BCR with high spatial and temporal resolution. We utilized the well-defined shift of the BCR to the lipid rafts in order to capture the signaling events and immediate cellular responses at 5, 10 or 15 min after IgM cross-linking, with the 1-min resolution window as allowed by APEX2. Our data, containing 1677 high-confidence hits, provides an encyclopedia of the proteins locating at the vicinity of the B cell plasma membrane and the lipid rafts while at the same time revealing the dynamics therein induced by IgM stimulation. We identify a wealth of previously uncharacterized proteins responding to the IgM signaling. As validation of our data, we show active SUMOylation at the sites of BCR signaling and demonstrate the functional role of SUMO in BCR signaling to AKT and ERK1/2 cascades.

## RESULTS

### Validation of a B cell line expressing lipid raft-targeted APEX2

**Fig. 1.**
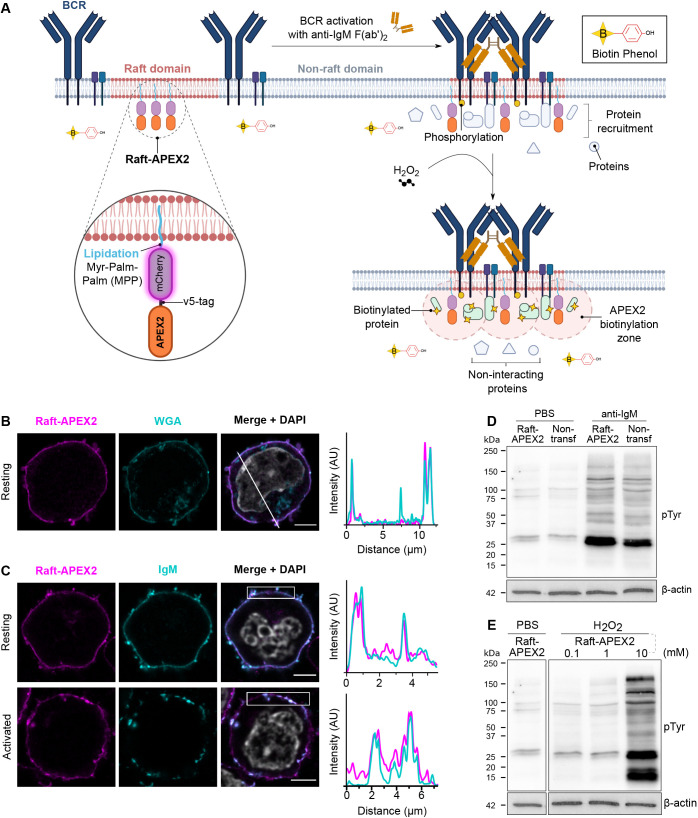
**Targeting of APEX2 to the lipid rafts to study B cell activation – design and validation**. (A) Schematic illustration of the raft-APEX2-mediated proximity biotinylation as a readout of the BCR signaling response. (B) A20 D1.3 B cells expressing raft-APEX2 were settled on fibronectin-coated glass coverslips for 1 h prior to fixation. Cells were stained with WGA as a membrane marker and DAPI for the nucleus. Left, Airyscan super-resolution confocal microscopy was used to image mCherry to visualize raft-APEX2 (magenta), WGA–Atto488 (cyan) and DAPI (gray, merge). Right, line scan analysis of the colocalization of raft-APEX2 and WGA. (C) Raft-APEX2-expressing cells were settled on a fibronectin-coated coverslip for 1 h, activated (bottom panel) or not (upper panel; resting) by addition of 1 µg of HEL antigen for 5 min, and fixed. The samples were stained for IgM and DAPI. Left, imaging was performed as in B to visualize raft-APEX2 (mCherry, magenta), IgM (cyan) and DAPI (gray, merge). Right, box scan analysis of the colocalization of raft-APEX2 and IgM. (D) Parental (non-transfected) and raft-APEX2-expressing B cells were stimulated with 10 µg/ml of anti-IgM F(ab′)_2_ for 10 min and subjected to immunoblotting with HRP-anti phospho-tyrosine antibodies and anti-β-actin as a loading control. See Fig. S1F for quantification. (E) Raft-APEX2 expressing B cells were treated with 0, 0.1, 1 and 10 mM H_2_O_2_ for 1 min and subjected to immunoblotting as in D. See Fig. S1E for the uncropped blot corresponding to D and E. All experiments were performed three times, representative examples shown.

In order to gain novel spatiotemporal information about the immediate cellular responses to BCR activation, we decided to employ APEX2-mediated proximity labeling promiscuously biotinylating proteins within a 20 nm range with a 1-min temporal resolution. The translocation of the BCR to the lipid raft regions of the plasma membrane provides a spatial window to study BCR signaling. We decided to fuse APEX2 with a specific 7 amino acid lipidation sequence, MGCVCSS, to target it to the lipid raft domains (raft-APEX2; Fig. 1A). The MGCVCSS sequence contains one myristoylation (Gly-2) and two S-acylation sites (Cys-3 and Cys-5) for palmitoylation, originally identified in the NH_2_-terminus of Lck. These modifications are responsible for the localization of Lck to the lipid rafts (Yasuda et al., 2000) and target fusion proteins to the membrane and the immunological synapse of T cells (Bécart et al., 2008; Bi et al., 2001). In addition, we equipped our APEX2 construct with an mCherry fluorescent protein to facilitate the detection of APEX2 expression (Fig. 1A, magnified circle).

We transfected raft-APEX2 into cultured A20 B cells that stably expressed transgenic hen egg lysozyme (HEL)-specific D1.3 IgM BCR (A20 D1.3) (Aluvihare et al., 1997) and generated a stable A20 D1.3 raft-APEX2 cell line. Flow cytometry analysis confirmed that cells positive for both mCherry^+^ and IgM^+^ composed >99% of the resulting cell line (Fig. S1A). To verify that raft-APEX2 indeed targets to the plasma membrane, we analyzed its localization using AiryScan confocal microscopy (Huff, 2015) to gain sufficient resolution to unambiguously detect signals deriving from the B cell plasma membrane. Raft-APEX2 clearly colocalized with the membrane marker wheat germ agglutinin (WGA), demonstrating strong enrichment at the plasma membrane (Fig. 1B). The lipid raft domains in resting cells are typically very small and transient in nature, making their detection highly challenging even with modern microscopy techniques (Gupta and DeFranco, 2003; Sezgin et al., 2017; Stone et al., 2017). Upon activation, BCR forms clusters that are rich in signaling activity and, at the same time, represent larger detergent-resistant membrane domains (Gupta and DeFranco, 2003; Mattila et al., 2016; Stone et al., 2017). Thus, we next activated the IgM BCR with the HEL antigen and followed the colocalization of IgM with raft-APEX2. As expected, upon cell activation, we detected increased clustering of the IgM, as well as enrichment of APEX2 in the same structures (Fig. 1C), indicative of localization in the IgM signaling clusters. However, a fraction of raft-APEX2 was also visible outside of the IgM clusters.

To investigate the lipid raft domain localization of raft-APEX2 in more detail, we adopted a flow cytometry-based assay (Gombos et al., 2004). We expressed, in A20 D1.3 cells, raft-APEX2 or model proteins resident either at detergent-resistant (caveolin-1–RFP; Martinez-Outschoorn et al., 2015) or detergent-soluble [hemagglutinin transmembrane domain (TDM) tagged with GFP; Nikolaus et al., 2010] membrane domains. The cells were treated with 0.1% Triton X-100 to release the detergent-soluble proteins from the plasma membrane, and a detergent resistance index was calculated based on the fluorescence before and after detergent treatment (Fig. S1B). The analysis showed that raft-APEX2 resisted the detergent treatment to a similar level with raft-resident caveolin-1 (Fig. S1C). The detergent-soluble model protein TDM-GFP, on the other hand, was almost completely removed from the plasma membrane upon detergent incubation. This analysis provided important support to our approach to use raft-APEX2 as a proxy to label proteins enriched at the lipid raft membrane domains and the vicinity of the signaling IgM.

We then proceeded to test for possible adverse effects caused by the expression of raft-APEX2 in our system. We detected undisturbed internalization of IgM upon receptor stimulation (Fig. S1D). Furthermore, upon activation by IgM cross-linking, the cells showed normal phosphorylation levels, as detected by anti-phospho-tyrosine antibodies, compared to the parental cell line (Fig. 1D; Fig. S1E,F).

Triggering of the biotinylation activity of APEX2 requires addition of H_2_O_2_. Notably, H_2_O_2_ has been shown to inhibit protein phosphatases and thereby is able to trigger signaling (Reth, 2002; Wienands et al., 1996). Importantly, we detected no increase in general protein phosphorylation upon incubation of cells with 1 mM H_2_O_2_ for 1 min, the conditions used to trigger biotinylation by APEX2, where as a 5–10 times higher concentration of H_2_O_2_ induced profound signaling, consistent with previous reports (Reth, 2002) (Fig. 1E; Fig. S1E,G,H). We then asked to what extent the response to these high levels of H_2_O_2_ resembles BCR signaling and compared the tyrosine phosphorylation pattern induced by 5 mM H_2_O_2_ to the pattern of IgM cross-linking. We detected some similarities but also profound differences between the two signals (Fig. S1I,J). Together, although it is possible that the 1 min treatment with 1 mM H_2_O_2_ facilitates some low-level phosphorylation, our data suggests that it has no significant effect to the BCR triggered phosphorylation events in our experimental conditions.

### Proteomic analysis of the lipid raft microenvironment identifies 1677 proteins

**Fig. 2.**
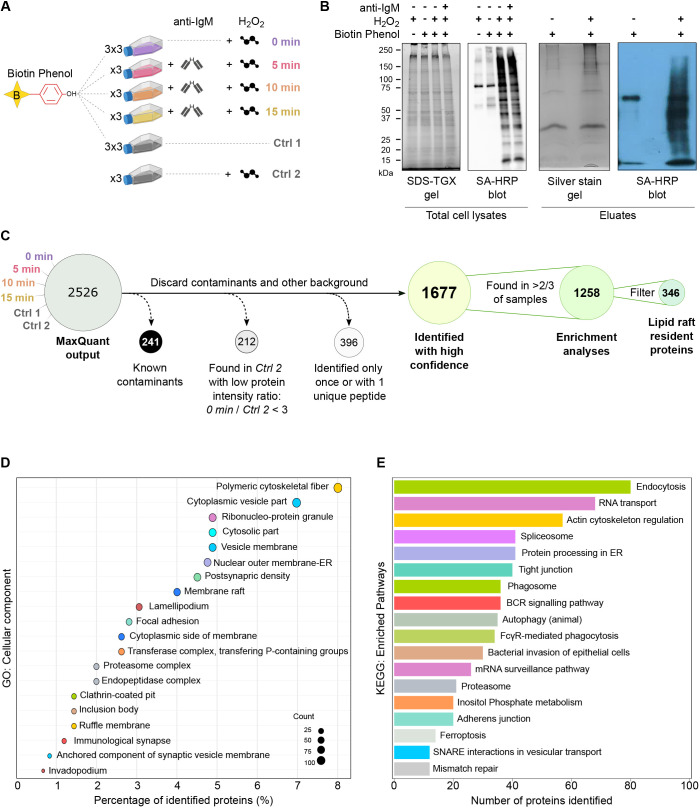
**Experimental design and pathway analysis.** (A) A schematic representation of the experimental samples and controls prepared and analyzed by quantitative label-free MS. (B) To monitor for basal levels of biotinylation, raft-APEX2-expressing A20 D1.3 B cells were subjected to anti-IgM stimulation (10 µg/ml, 10 min), H_2_O_2_ (1 mM, 1 min) or biotin-phenol (500 µM, 45 min), or combinations of these conditions. Left, total cell lysates were analyzed with TGX Stain-Free SDS gel and a streptavidin–HRP immunoblot. Right, the samples eluted from the streptavidin-coated beads were analyzed with a Silver-stained SDS-PAGE and a streptavidin–HRP immunoblot. Experiments were performed three times, representative examples shown. (C) Flow chart with the filtering steps used for the data analysis. (D) Classification of the 1677 identified proteins based on cellular component gene ontology (GO) terms. (E) A KEGG pathway enrichment analysis for the 1677 identified proteins shows the cellular pathways enriched among the identified proteins. To remove redundancy both in the GO terms and in the identified pathway terms, the terms with ≥50% similarity were grouped, and the one with the lowest adjusted *P*-value is shown.

For preparing the proximity biotinylation samples, biotin-phenol supplemented cells were activated with the potent surrogate antigen, F(ab′)_2_ fragments of anti-IgM antibodies for 0, 5, 10 or 15 min (Fig. 2A). The biotinylation was triggered by adding 1 mM H_2_O_2_ and quenched after 1 min. The biotinylation efficiency was verified in each set of samples by flow cytometric analysis, which typically showed biotinylation in ∼70% of cells (Fig. S1K). Lysed cells were subjected to streptavidin affinity purification to pull down the biotinylated proteins for MS (Fig. 2B). To assess the baseline activity of APEX2 and the contribution of endogenous biotinylation, control samples without H_2_O_2_ (Ctrl 1) or without biotin-phenol (Ctrl 2), respectively, were prepared. Trypsin-digested samples were analyzed by liquid chromatography-electrospray ionization tandem MS (LC-ESI-MS/MS) and peptide/protein calling was done with MaxQuant software (Table S1A) (Cox and Mann, 2008). Differential analysis was done using NormalyzerDE (Willforss et al., 2019). After filtration of known contaminants and background, we found 1677 proteins with two or more unique peptides identified (Fig. 2C). High confidence hits from all experimental conditions together are listed in Table S1B. As expected, we detected, with very high intensity values, several proteins associated with lipid rafts and BCR signaling (Tables S2, S4A; Fig. 2D,E). At the same time, the large total number and variety of identified proteins illustrate the efficacy of APEX2-mediated protein biotinylation that also reaches the cytosolic environment immediately beneath the membrane.

### Lipid raft-resident proteins feature stable localization at the rafts

**Fig. 3.**
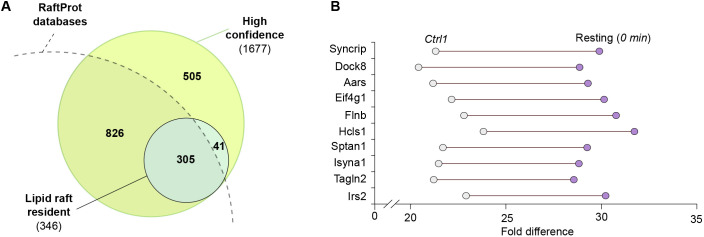
**Lipid raft-resident proteins.** (A) A comparison of the lipid raft-resident proteins identified in this study and the whole dataset (as per Fig. 2C) to the RaftProt database, showing the overlap between the datasets. (B) The top ten proteins that show the highest enrichment in the lipid rafts of B cells as reported by raft-APEX2-mediated biotinylation prior (control 1) and post addition of H_2_O_2_ (resting, 0 min), ranked by the difference in the protein intensities between the two conditions.

To further validate the lipid raft localization of our raft-APEX2 construct, we first shortlisted our data for proteins that were likely to reside at the closest vicinity to raft-APEX2. APEX2 exhibits basal activity that leads to low-level release of biotin radicals with the aid of endogenous H_2_O_2_. Using a similar approach to that in Paek et al. (2017), we took an assumption that the proteins locating at the closest vicinity to the raft-APEX2 are already prone to biotinylation before extracellular addition of H_2_O_2_ and get very efficiently labeled upon the addition of H_2_O_2_, yielding high peptide intensities in the MS data. From the proteins identified in Ctrl 1 sample (without added H_2_O_2_), we selected those that showed a notable ≥1.5 log_2_ fold change upon triggering of biotinylation by H_2_O_2_. Additionally, we filtered out proteins that responded with ≥1.0 log_2_ fold change to IgM stimulation to selectively shortlist only those that were constantly at the closest vicinity of raft-APEX2. This resulted in the identification of 346 proteins that we considered as B cell raft-resident proteins (Table S2). Almost 90% of the proteins were also found in the available RaftProt databases (Mohamed et al., 2019) (Fig. 3A; Fig. S2A), providing further confidence in the preferred raft localization of our construct.

Among the strongest raft-resident proteins were, for instance, Dock8 and Hcls1, reported regulators of BCR signaling and B cell activation (Hao et al., 2004; Randall et al., 2010), as well as filamin and spectrin, scaffold proteins linking the plasma membrane and the underlying cytoskeleton (Liem, 2016; Razinia et al., 2012) (Fig. 3B). An earlier study identified 34 proteins in the isolated detergent-resistant membrane domains from human Raji B cells (Saeki et al., 2003). Out of these 34, our approach identified 20, ten of which were among the 346 prominent raft-resident proteins (Fig. S2A; Table S2). The discrepancy could result from the differences between the *in vitro* biochemical fractionation and labeling in cells, or simply the different cell line used.

### B cell membrane-proximal proteome reveals a variety of different protein groups

To obtain a broad view of the complete set of 1677 proteins identified in our study, we used Gene Ontology (GO) cellular components analysis (Ashburner et al., 2000) and Kyoto Encyclopedia of Genes and Genomes (KEGG) pathway assignment (Kanehisa et al., 2016). We identified the highest protein counts in various cytoskeletal and membrane structures linked to fundamental cell biological pathways, such as regulation of the actin cytoskeleton and endocytosis (Fig. 2D,E). Among the more specific terms, we found ‘Membrane rafts’, ‘Immunological synapse’ and ‘BCR signaling’ to be significantly enriched in our data, providing confidence in our approach to detecting changes in the protein environment linked to BCR signaling.

**Fig. 4.**
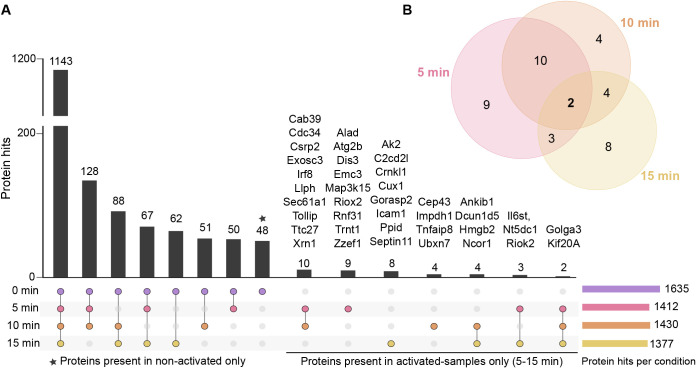
**Proteins identified in different conditions of BCR activation.** (A) An upset plot showing the numbers of proteins identified in each experimental condition of our proteomic dataset (Fig. 2C). When ten or fewer proteins were identified, the names of the identified proteins are shown on top of the bar. (B) A Venn diagram showing intersections of proteins from A, identified in activated samples only (5, 10 and 15 min).

Out of the total of 1677 high-confidence hits in our data (Table S1B), a large majority, 1143 proteins, were common to all conditions. A total of 48 proteins were specific to resting cells, and 40 were specifically detected only upon IgM activation (Fig. 4A,B; Table S1C,D). Only two proteins, Kif20a and Golga3, were identified in all activation time points and not in any of the non-activated controls (Fig. 4A,B; Table S1D).

**Fig. 5.**
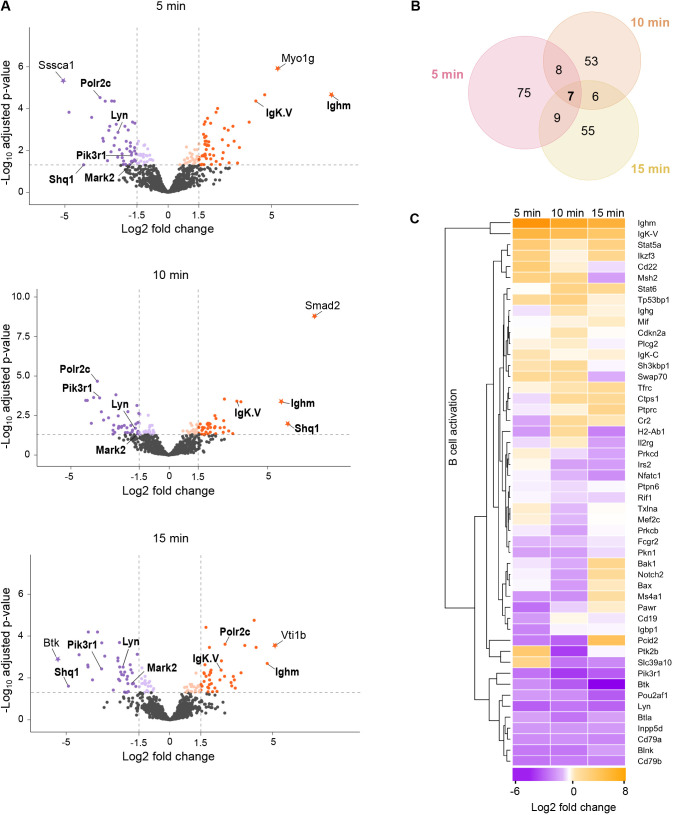
**Enrichment analysis.** (A) Volcano plots illustrating the detected protein intensity dynamics upon anti-BCR activation at 5, 10 and 15 min. The data are based on the differential enrichment analysis of 1258 identified proteins (Fig. 2C). The proteins showing statistically significant (adjusted *P*≤0.05) enrichment in non-activated conditions are shown in violet and in activating conditions in orange. The proteins with a log_2_ fold change ≥1.5 are further denoted with a stronger color tone. The names of the seven proteins differentially enriched in all three time points are shown in bold. The proteins with a log_2_ fold change >5 are denoted with a star symbol. (B) A Venn diagram showing the numbers of significantly enriched proteins with log_2_ fold change ≥1.5 at any time point and their intersections. In total, this comprises 213 proteins. (C) A heatmap of proteins classified to GO term ‘B cell activation’ showing the changes in the protein intensity at different time points of BCR activation.

For statistical analysis of the changes in protein abundance at different conditions, we first applied the criteria of the proteins needing to be present in at least 12 out of 18 experimental samples, which restricted the analysis to 1258 proteins (Figs 2C, 5A,B; Table S3A). The missing values were imputed using k-Nearest Neighbor (kNN), and quantitative differential analysis was undertaken using NormalyzerDE (Willforss et al., 2019). The majority of the proteins did not undergo significant dynamics upon IgM activation but instead showed relatively stable abundance throughout different conditions. A total of 213 proteins showed significant dynamics with log_2_ fold change ≥1.5 or≤−1.5 upon cell activation (Fig. 5B; Table S3B). Distinct sets of proteins were found to be enriched or diminished at different time points. Whereas 99, 74 and 77 proteins were significantly altered at the 5, 10 and 15 min time points, respectively, only seven proteins were found to be significantly altered at all the studied time points (Fig. 5A,B). These findings suggest that although most of the proteins do not dramatically change their localization, a minor fraction of proteins respond by notable changes regarding their vicinity to the lipid rafts.

Interestingly, proteins associated with transcriptional regulation, as defined by belonging to the functional category ‘transcription’ in the DAVID knowledgebase (Huang et al., 2009a,b), constituted ∼10% of the detected proteome. For instance, we identified the NF-κB pathway proteins, c-Rel, RelA, NF-κB2 and regulatory IκBα (also known as NFKBIA), which also showed dynamic behavior upon IgM activation (Fig. S3A). FoxO1 transcription factor, whose translocation out of the nucleus is mediated by phosphoinositide 3-kinase (PI3K)-AKT signaling (Brunet et al., 1999), enriched with raft-APEX2 at 15 min after activation. The transcription factors particularly important for B cell development and differentiation, IRF5 and IRF4, were also detected, particularly IRF5 showing strong raft proximity in all conditions. This could relate to the association of IRFs with membrane-proximal TLR adapter protein MyD88 and Src kinases, including Lyn (Negishi et al., 2005; Nishiyama et al., 2016).

### The dynamics of proteins linked to BCR signaling

Importantly, Ighm and IgK-V, the heavy and light chain components of the transgenic IgM BCR specifically stimulated in our setup, were strongly enriched in the rafts of activated samples (Table S3A,B; Fig. 5A,C). In contrast, Ighg, the heavy chain of the endogenous IgG2a BCR, which was not engaged by the surrogate antigen, remained essentially unchanged upon receptor activation. This notion proposes that the mechanisms driving activated BCR to the lipid raft domains have specificity to engaged receptors and do not carry a notable bystander effect, at least in an inter-isotype manner that would change the localization of the non-ligated BCRs. However, in comparison to IgM, IgG2a was already detected at higher levels in resting cells, suggesting a stronger tendency of this BCR isotype to localize to lipid rafts in resting cells (Table S2). Interestingly, despite substantial enrichment of IgM heavy chain and κ light chain to the lipid rafts upon activation, we saw a decrease in the abundance of the Igα and Igβ (also known as CD79A and CD79B, respectively), proteins essential for both signal transmission and the stability and membrane transport of the BCR (Fig. 5C). This finding could be caused by increased shielding of the phosphorylated cytoplasmic tails of the Igα and Igβ upon receptor triggering, as a result from the recruitment of the downstream signalosome components. Alternatively, this finding could indicate that the local ratio of Igα and Igβ to the BCR heavy chain is not fixed but can be tuned depending on the membrane location and/or the activation state.

When analyzing the known components of the BCR signaling pathway, the results were somewhat unexpected. We did not identify prominent BCR regulator proteins among the proteins exclusively found either in resting cells or activated cells (Fig. 4A), suggesting instead that there are incremental changes in the signaling protein localization rather than dramatic translocations induced by IgM engagement. Furthermore, various components of the BCR signaling pathway either did not show significant dynamics, or instead showed diminution while the IgM enriched to the lipid rafts (Fig. 5C). This finding is consistent with proximal receptor signaling cascades being typically heavily dependent on protein modifications such as phosphorylation, which are not necessarily reflected as changes in total protein localization.

Among the identified tyrosine kinases involved in early BCR signaling were Lyn, Fyn, Blk and Btk (Table S1B). Lyn is considered one of the central kinases triggering BCR signaling owing to its early requirement to phosphorylate Igα and Igβ ITAM motifs, and it also preferentially locates to the lipid rafts (Xu et al., 2005). Accordingly, we found Lyn in the rafts in all conditions but with significantly diminished intensity upon IgM activation. Previous studies, by total internal reflection fluorescence microscopy of B cells activated on bilayers, have indicated that the closest vicinity of Lyn to the BCR, measured by fluorescence resonance energy transfer, is seen within the first 200 s of BCR activation, after which the interaction diminishes (Sohn et al., 2008). The mechanistic details of Lyn are further complicated by the dual role of the kinase as both a negative and positive regulator of BCR signaling (Xu et al., 2005). The triggering of the BCR signaling cascade is shared with Fyn, another Src-family protein tyrosine kinase (Xu et al., 2005). In our data, Fyn shows higher abundance across the conditions and seems to be largely located at the lipid raft-like regions. Another Src-family kinase identified in our data was YES1. Although not commonly linked to BCR signaling, we detected a significant enrichment of YES1 at the lipid rafts at 5 min activation. Notably, we did not detect the prominent BCR proximal kinase Syk in our dataset. To test for possible alterations in the expression levels of Syk in our cell line, we performed immunoblotting. We detected normal levels of both total Syk and Syk phosphorylation in raft-APEX2 cells as compared to the parental A20 cells (Fig. S2C,D), suggesting that the lack of identification of Syk in the dataset could result from inefficient biotinylation due to steric obstruction or a lack of biotinylation-suitable and available amino acid moieties on the protein surface.

From the other components of the BCR signaling pathway, for example, the activatory co-receptor CD19 was constantly found in high abundance in all samples and classified as a lipid raft-resident protein (Table S2) (Fig. 5C). The reported enrichment of the CD19 in the BCR signaling microclusters (Depoil et al., 2008; Mattila et al., 2013) could, thus, reflect gathering of the raft domains already containing CD19. Two other transmembrane proteins linked to BCR activation, predominantly as negative regulators, the sialic acid-binding Ig-like lectin protein CD22 and FcgR2, were also identified in the data. Whereas CD22 showed enrichment to the lipid rafts at 5 min of activation, FcgR2 was defined as lipid raft-resident protein.

Btk, an essential regulator of BCR downstream signaling, showed increasingly strong negative fold-change upon activation, indicating exclusion from the forming BCR clusters in these settings. Btk is known to be recruited to the plasma membrane by PI(3,4,5)P_3_ phosphoinositide, a signaling lipid critical for B cell activation (Saito et al., 2001). Consistent with this, we detected a substantial diminution of the regulatory subunit of PI3K, Pik3r1, from the lipid rafts upon cell activation. These notions would suggest early separation of the inositol trisphosphate (IP_3_) signaling from the immediate vicinity of the BCR. B cell linker (BLNK; also known as SLP65), a binding partner of Btk and various other BCR signaling proteins, showed substantial abundance in the rafts throughout the time points but also significant downregulation upon BCR activation. The recently demonstrated phase-separation properties of BLNK could also be related to its strong localization to ordered lipid domains (Wong et al., 2020). Phospholipase-γ2 (Plcγ2), which forms the other branch of lipid signaling downstream of BCR, was found constitutively present as a lipid raft-resident protein (Table S2). In summary, intriguingly, we found BCR signaling proteins mostly either non-dynamically raft-resident or decreasing from the raft regions upon activation.

### Proximity proteomics identifies multiple vesicle trafficking proteins responding to BCR signaling

We detected substantial dynamics of various proteins linked to the steps subsequent to BCR activation, such as cytoskeleton remodeling, endocytosis and membrane trafficking, all of which are essential for internalization of BCR–antigen complex and further processing for antigen peptide presentation. Our data illuminates the employment of different regulators of these processes, highlighting, for example, the existence of various components of the clathrin-mediated endocytosis (Table S4, Fig. S3B). Several of them, such as Cltc, Hip1R and Eps15, were detected as lipid raft-resident proteins (Table S2), or were found differentially enriched during IgM activation (Table S3), such as the α- and β-AP2 complex subunits.

We next sought to validate some of the proteins that were highlighted in our data but not previously linked to BCR signaling. Our attention was drawn to various candidates linked to intracellular membrane traffic that showed specific recruitment towards the lipid rafts upon IgM engagement. For instance, the two proteins identified solely in activatory conditions, golgin subfamily A member 3 (Golga3) and the kinesin Kif20a (Fig. 4), are both associated with the endomembrane system but have not previously been associated with BCR signaling. In order to verify the observed dynamics of these proteins, we turned to microscopy. We could not find immunofluorescence-compatible antibodies against Kif20a, but went on to visualize Golga3, a poorly understood multifunctional peripheral membrane protein linked to regulation of membrane transport of selected plasma membrane proteins (Hicks et al., 2006; Hicks and Machamer, 2005; Williams et al., 2006), ubiquitylation (Dumin et al., 2006), apoptosis (Maag et al., 2005) and also to dynein function (Yadav et al., 2012). In Raji D1.3 human B cells, which were chosen due to these working better with the anti-Golga3 antibodies, we found that Golga3 was widely distributed in the cells in a vesicular fashion. The distribution of Golga3 vesicles was indeed altered upon IgM engagement, and the vesicle pool at the cell membrane became more prominent (Fig. S4A). Utilizing cell volume segmentation based on microtubule staining (Fig. S4B), we selectively analyzed the Golga3 vesicles at the vicinity of the plasma membrane before and after IgM activation. We found that the Golga3 vesicles became significantly larger and brighter in the activated cells than the non-activated counterparts (Fig. S4D). Also, the shape of the vesicles became more elongated, and the vesicles showed notable yet partial colocalization with internalized surrogate antigen. A colocalization analysis at the cell periphery (determining Manders' overlap coefficients, M1 and M2) revealed marked colocalization of both Golga3 signal in antigen clusters (M1: 0.61) and antigen signal in Golga3 vesicles (M2: 0.56) (Fig. S4C). Thus, the immunofluorescence data well supported our proteomics data and showcased Golga3 as a novel protein translocating to the proximity of the BCR upon antigenic activation.

### SUMOylation regulates BCR signaling and immunological synapse formation

To gain a deeper insight on our dataset and uncover more influential proteins or groups of similarly behaving proteins, we performed further bioinformatic analysis on our list of differentially expressed proteins (Table S3) using unsupervised machine learning and k-means clustering with protein expression log_2_ fold-changes as predictors (Gu et al., 2016; Hartigan and Wong, 1979). The k-means clustering algorithm grouped the proteins into 24 different groups (Fig. S5), supporting a diverse activation of multiple cellular processes, which we classified based on the major GO biological processes enriched in each group using DAVID (Jiao et al., 2012). We became interested in the second largest group, group number 10, containing proteins with gradual increase in their enrichment and classified to be involved in protein transport, nuclear transport and SUMOylation. In this group, we found RanGAP1 and small ubiquitin-like modifier 1 (SUMO1) that complex with RanBP2 SUMO E3 ligase, also identified in our dataset, to regulate nuclear transport of RNAs and proteins (He et al., 2021; Okamura et al., 2015). Notably, post-translational modification of proteins by addition of SUMO moieties regulates a wide variety of cellular functions, such as DNA repair and replication, signal transduction, cell division and cell metabolism (Chang and Yeh, 2020; Courtois and Fauvarque, 2018). In our complete APEX2 biotinylated dataset, we identified nine proteins linked to SUMOylation (Fig. S6A).

**Fig. 6.**
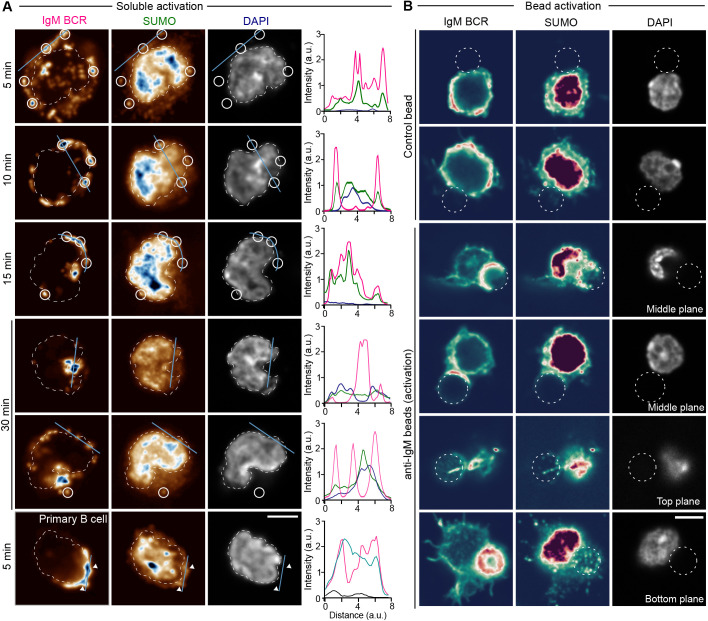
**Enrichment of SUMOylation at the sites of BCR activation.** (A) A20 D1.3 cells or primary mouse B cells were activated with fluorescently labeled anti-IgM (soluble surrogate antigen in magenta, 5–30 min), fixed and stained with anti-SUMO [pseudocolor lookup table (LUT)] and DAPI (gray). The dashed white line outlines the nucleus based on DAPI. The white circles highlight SUMO enrichment colocalizing with anti-IgM clusters. The cyan line represents the line profile shown on the right side of the images (magenta for anti-IgM, green for anti-SUMO, and dark blue for DAPI intensities). (B) A20 D1.3 cells were incubated with control or anti-IgM coated beads for 30 min, fixed and stained with anti-IgM antibodies, anti-SUMO and DAPI. The channels are shown using a pseudocolor LUT or greyscale (DAPI). The white dashed line shows the position of the bead. Scale bar: 5 µm. All experiments were performed three times, ∼10–30 cells/condition/experiment imaged, and representative examples shown. a.u., arbitrary units.

To ascertain a possible involvement of SUMOylation in B cell activation, we went on to investigate the localization of SUMO upon BCR activation. We activated A20 D1.3 B cells with fluorescent anti-IgM F(ab′)_2_ for 5, 10, 15 and 30 min, and performed immunofluorescence staining for SUMO1. We observed a very strong signal of SUMO1 in the nucleus, reflecting its prominent nuclear functions. However, we also indeed observed a clear colocalization of IgM BCR and SUMO in the BCR clusters in the cell periphery, especially at early time points of 5–15 min after activation (Fig. 6A). After 30 min of activation, when the large part of the internalized antigen is clustered to the antigen-processing compartments (Hernández-Pérez et al., 2020) the colocalization with SUMO was notably reduced and only some peripheral antigen clusters still showed colocalization. We were also able to see colocalization of BCR and SUMO1 in primary B cells isolated from mouse spleen, with clear accumulation of SUMO1 at the sites of polarized BCR accumulation, the so-called BCR cap, which is typical for primary B cells (Fig. 6A). The strong nuclear SUMO1 signal significantly challenged the quantitative colocalization analysis in the cells activated with soluble surrogate antigen. We next activated the A20 D1.3 B cells with antigen-coated beads, mimicking an antigen-presenting surface and immunological synapse formation. Again, we saw clear punctate recruitment of SUMO1 to the activatory site, that is the surface of the bead, together with BCRs (Fig. 6B). Such SUMO1 accumulation was not seen with non-activatory control beads (Fig. 6B), although on fibronectin-coated glass SUMO signal was seen to also decorate some of the BCR clusters especially along the filopodia (Fig. S6B). Of note, in the 3D stacks of the cells, we frequently noticed another bright SUMO1 cluster close to the nuclear signal. We identified this structure as microtubule-organizing center (MTOC) by polarizing the cells by letting them adhere on coated microscope slides and staining them for the MTOC marker PCM1. A SUMO1 signal was clearly enriched in the MTOC of B cells regardless of their activatory status (Fig. S6C,D).

**Fig. 7.**
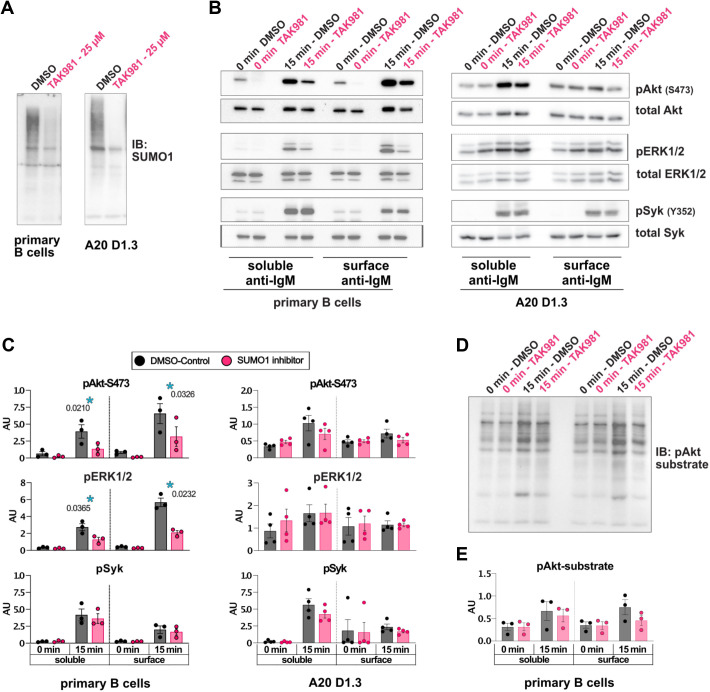
**Pharmacological inhibition of SUMOylation leads to reduced AKT and ERK1/2 signaling downstream of BCR activation.** (A) An immunoblot showing reduction in SUMO1 signal upon 30 min incubation of mouse primary B cells with 25 µM TAK981 (*n*=3). (B) A20 D1.3 (*n*=4) or mouse primary B cells (*n*=3) were pre-treated with 25 µM TAK981 for 30 min, stimulated with surface-bound or soluble F(ab′)_2_ mouse anti-IgM (10 µg/ml) for 15 min in the presence of TAK981 and cell lysates were analyzed for levels of pAkt, pERK1/2 and pSyk. (C) Quantification of the data shown in B. (D) Primary B cells stimulated as in B were analyzed by immunoblotting using pAkt-substrate antibodies recognizing phosphorylated (RXXS*/T*) sequences (*n*=3). (E) Quantification of data in D. Repeated measures two-way ANOVA, shown as mean±s.e.m., *P<0.05.

To test for possible functional effects of SUMOylation, we utilized a pharmacological inhibitor of SUMOylation, TAK981, which selectively inhibits the SUMO-activating enzyme (SAE) complex that catalyzes the first step in the SUMOylation cascade (Langston et al., 2021; Lightcap et al., 2021), and which was also found in our dataset (Fig. S6A). Inhibition of protein SUMOylation by TAK981 at a concentration of 25 µM for 10–30 min efficiently cleared SUMOylation pattern of proteins visible on an immunoblot (Fig. 7A). We first probed for the A20 D1.3 cell spreading on the antigen-coated surfaces, mimicking immunological synapse formation. We saw reduced intensity of filamentous actin structures in cells treated with TAK981 at 10 and 15 min after activation, indicating lowered efficiency in forming the synapse. Despite a similar trend, we however, did not detect a significant reduction in the overall tyrosine phosphorylation in this set-up (Fig. S6E,F).

We then probed for the activation of phosphorylation cascades downstream of BCR upon SUMOylation inhibition both in mouse primary B cells and in the A20 D1.3 cell line using either soluble antigen activation or activation by surface-tethered antigens, mimicking immune synapse formation. In our western blot analysis, we found a significant reduction in both phosphorylated (p)AKT (herein AKT refers to AKT1) and pERK1/2 (MAPK3/1) levels in both soluble and surface-bound activating conditions in primary mouse B cells upon inhibition of SUMOylation (Fig. 7B,C). Of note, phosphorylation of Syk was unchanged, suggesting that the effect of SUMO occurs at the level of downstream signal regulation. However, no significant functional defects were seen in the A20 D1.3 B cell line, fitting with only small defects detected in the spreading response. Finally, we probed for the efficiency of AKT to phosphorylate its target proteins in the conditions when SUMOylation was inhibited, using an antibody that recognizes the phosphorylated forms of AKT substrate signature sequences. Although no statistical significance was reached, we saw indications of reduced AKT substrate phosphorylation upon TAK981 treatment in primary B cells, particularly when activated on surface-tethered antigens (*P*-value=0.0524; Fig. 7D,E).

## DISCUSSION

To better understand BCR signaling and the immediate, multi-branched cellular responses it triggers, the development of improved large-scale approaches with sufficient spatiotemporal resolution is critical. Here, we pioneer an APEX2-mediated proximity biotinylation proteomics approach to track large-scale protein dynamics at the plasma membrane lipid raft domains, where BCR translocates upon activation. As APEX2 efficiently biotinylates its vicinity in the range of 20 nm on the time scale of 1 min, it poses significant power to report on protein abundancies at a large scale in a time-resolved manner. By identifying and quantitatively analyzing over 1600 proteins, we draw a landscape of proteins at, or very close to, the plasma membrane in B cells and report their dynamics during IgM BCR activation. Furthermore, our data proposes various new protein players responding to the IgM engagement, out of which we validated a vesicle traffic regulator, Golga3, and SUMOylation. We also demonstrate a functional role of SUMOylation on AKT and ERK1/2 activation and show that, in primary mouse B cells, acute lack of SUMOylation during BCR signaling disrupts signal propagation.

With its high efficiency, APEX2-based proximity proteomics provides a sought-after opportunity for an ensemble view of the various cellular machineries triggered upon BCR activation. We faced challenges in fusing APEX2 to the signaling subdomains of the BCR, namely Igα or Igβ, and to the proximal signaling protein Lyn, which all failed to get successfully expressed in B cells, despite the same constructs being well expressed in fibroblast type of cells. For this reason, we took advantage of the well-described association of the receptor with the lipid rafts induced upon activation. APEX2 targeting by a lipid raft-directed lipidation sequence minimized the risk of interference with the signaling cascades while still reporting about BCR vicinity with reasonable accuracy. Although a fraction of the probe might also remain in the non-raft regions, strong preference for lipid rafts was clear, based on our microscopic and flow cytometric assays (Fig. 1; Fig. S1A,B). Also, in our shortlisting of the most efficiently and constitutively biotinylated proteins, 90% were found in the RaftProt database (Table S2). We also saw a drastic enrichment in IgM BCR in activatory conditions (Fig. 5), further arguing for the raft targeting of the APEX2 and the validity of this approach to report on the IgM-proximal proteome.

Of the 1677 proteins identified, a vast majority were detected, at least to some level, both in resting and activatory conditions. This can be a consequence of the known heterogeneity of the raft domains (Sezgin et al., 2017) or simply from the high sensitivity of APEX2-mediated biotinylation, leading to the detection of the proteins also present at low levels. A total of 88 proteins were selectively identified either in resting or activated cells (Fig. 4). Additionally, the quantitative analysis revealed 213 proteins with a condition-specific enrichment profile (Fig. 5B). The relatively small proportion of differentially behaving proteins is consistent with the fact that only a few changes have been reported to occur in the isolated lipid rafts upon BCR signaling (Gupta et al., 2006). Unfortunately, the study of Gupta and colleagues only reports on a selected and limited set of 39 proteins in rafts making thorough comparisons between the data impossible. The highly dynamic nature of the BCR signaling response was apparent in the data, such that several changes occurring at 5 min after activation, for instance, were seen reset by 10 or 15 min (Fig. 4). Interestingly, in general, we found slightly more proteins that were diminished rather than enriched at lipid rafts upon signaling. This is in agreement with the fusion and stabilization of lipid rafts to promote signaling microclusters concomitant with the coming together of smaller nanoclusters (Gupta and DeFranco, 2003; Mattila et al., 2013, 2016; Stone et al., 2017), which could reduce the detection of proteins locating preferentially at the borders or surroundings of the rafts. The presence of 48 proteins exclusively in non-activated samples (Fig. 4A; Table S1C) shows an IgM-induced exclusion of quite a substantial set of proteins, perhaps partially reflecting the reorganization of the plasma membrane but also suggesting interesting new players orchestrating the signaling cascades. Also, although the abundance of the IgM BCR itself drastically and clearly increased upon activation, the known components of the BCR signaling pathway showed variable responses (Fig. 5C). For example, we noticed an interesting reduction in the abundance of Lyn, Btk and the Igα and Igβ signaling sheath of the BCR, which could indicate that the signaling pathways partly separate from the lipid rafts, which on the other hand, become platforms for BCR endocytosis. Such separation of IgM and Igα and Igβ sheath has been previously suggested by the finding that Igα and Igβ co-precipitate 50–80% less with IgM at 15–30 min after receptor cross-linking as compared to in the resting conditions (Vilen et al., 1999). This finding would also fit with the dissociation-activation model of BCR activation, where opening-up of the BCR oligomers and the Igα and Igβ sheath is proposed as a driver of receptor activation (Yang and Reth, 2010). Furthermore, mutagenesis studies on the intracellular tails of Igα and Igβ have proposed Igα and Igβ signaling and internalization as mutually exclusive events, such that the phosphorylated proteins would remain on the cell surface, whereas non-phosphorylated forms are internalized (Hou et al., 2006).

As expected, we found several BCR signaling proteins readily located in the lipid rafts, as suggested by the robust detection of many of them already in the steady-state (Table S1) as well as the raft-resident, non-BCR-responsive localization of some of them, such as Plcγ2 and CD19 (Table S2). Thus, the translocation of the engaged BCR to the rafts could promote signaling in an energy-efficient manner, as it reduces the need for various other concerted protein translocations.

A crucial role in BCR signaling is played by various protein phosphorylation cascades that have been studied in good detail. Rapidly adjustable post-transcriptional modifications are well-suited drivers for fast signaling events and might not always go fully hand-in-hand with the potentially slower changes in protein localization reported by our proteomic study. It is also important to note that although a wealth of our knowledge on BCR signaling comes from studies using soluble antigen, the specific details about signaling protein recruitment are derived mainly from microscopy studies using surface-bound antigens and processes might differ between different forms of antigenic stimuli (Depoil et al., 2008; Harwood and Batista, 2010; Kuokkanen et al., 2015; Mattila et al., 2016). Next, it will be very interesting to apply proteomics to the B cell activation by different types of antigens, including surface-bound antigens, such as in Cunha et al. (2023a,b preprint). During the revision phase of our manuscript, a preprint publication on a large proximity biotinylation study was disclosed, where the authors tracked protein dynamics at the vicinity of the APEX2-tagged CD19 from 10 s to 2 h after BCR activation (Susa et al., 2023 preprint). A deeper comparison between the datasets might provide further insights in BCR activation dynamics.

Our dataset also contained a significant fraction of proteins linked to cellular machineries that are generally not associated with the plasma membrane, such as ribosomes, regulation of translation and RNA transport (Fig. 2E). Despite various controls included in our study, some background binding to the streptavidin beads, or biotinylation by the non-localized pool of APEX2 that did not yet reach the cell membrane, is possible. However, disputing the possibility of being just unspecific background, many of these proteins showed marked intensity and qualified as B cell lipid raft-resident proteins or showed significant enrichment upon activation. Notably, two independent studies in T cell hybridoma and prostate cancer cells have suggested a set of ribosomal and nuclear proteins to undergo S-acylation and discovered their targeting to the lipid rafts (Martin and Cravatt, 2009; Yang et al., 2010). Also, increased phosphorylation of eIF3 complex proteins has been observed upon antigen stimulation of B cells (Matsumoto et al., 2009), further advocating that some translational regulators could be early targets of BCR signaling. Membrane localization might serve a regulatory role for these transcriptional and translational regulators, and BCR activation, with its gathering to the lipid raft domains, could, either directly or indirectly, induce the release of this reserve. The possible unexpected relationships, suggested by our data, between BCR signaling, plasma membrane and proteins playing a role in translational regulation, RNA transport and nuclear transport are attractive topics for future studies.

As we know from previous studies, a large portion of the antigen-BCR complexes are internalized soon after BCR activation, and the complexes are rapidly targeted to antigen processing compartments (Hernández-Pérez et al., 2020). Accordingly, many of the proteins identified with a marked dynamic response to IgM signaling were linked to different branches of intracellular vesicle trafficking or cytoskeletal reorganization. We validated the activation-induced translocation of endomembrane-associated protein Golga3 towards the plasma membrane microscopically and found changes in the vesicular appearance of Golga3. In the literature, Golga3 has been proposed to recruit cytoplasmic dynein, a minus-end microtubule motor protein, to the Golgi and to be responsible for the positioning of the Golgi close to the centrosome (Yadav et al., 2012). Comparably, Golga3 could be involved in the centripetal movement of internalized antigen vesicles in B cells. As part of a parallel study, we also followed up on another endomembrane trafficking-linked hit from our study, a Q-SNARE Vti1b (vesicle transport through interaction with t-SNAREs homolog 1B), which showed a very strong fold-change in our dataset, being the most enriched protein at 15 min time point (Fig. 5A). We characterized its enrichment to the sites of BCR signaling, further supporting the potential of our dataset (Music et al., 2022).

Interestingly, our dataset revealed various proteins linked to SUMOylation pathways. We validated the enrichment of SUMO1 at the sites of BCR activation both upon activation by soluble antigen as well as bead-bound antigen, mimicking immunological synapse formation (Fig. 6). Furthermore, by utilizing an inhibitor for SUMOylation, TAK981, we showed that intact state and dynamics of global protein SUMOylation at the time of BCR triggering contributes to the formation of the B cell immunological synapse and is required for proper phosphorylation of AKT and ERK1/2, the prominent kinases downstream of BCR activation (Fig. 7). The experiments using an antibody recognizing the phosphorylated consensus sequences of AKT substrates, suggested a reduction in AKT downstream activity upon surface-bound, immune synapse-like, antigen activation. Pronounced downregulation of pAKT and pERK1/2 in TAK981-treated normal non-activated WT B cells also suggests that SUMOylation dynamics could be necessary for tonic BCR signaling and may contribute to the previously reported more pronounced depletion of B cells rather than T lymphocytes in mice subjected to multiple exposures of tolerated doses of TAK981 (Lightcap et al., 2021). Although they exhibited clear enrichment of SUMO-conjugated proteins at the sites of BCR activation, A20 D1.3 B cells, however, seemed functionally less sensitive to SUMOylation inhibition. This indicated a somewhat different wiring of the signaling cascades in the primary B cells versus A20 lymphoma B cells.

Altogether, our results draw an important picture of the overall proteome at the B cell plasma membrane and provide both a comprehensive view and unprecedented information on the protein dynamics responding to BCR engagement. In addition to reporting on antigen receptor signaling, our work describes the lipid raft microenvironment in lymphocytes. As lipid rafts have been identified as hotspots for various membrane receptors and signal transduction machineries (Mollinedo and Gajate, 2020; Varshney et al., 2016), our approach can serve as an easily adaptable platform also for studies of other signaling systems.

## MATERIALS AND METHODS

### Design and cloning of raft-APEX2

pcDNA3-mito-APEX (Rhee et al., 2013; Addgene plasmid #42607, deposited by Alice Ting), was used as a template to create and PCR amplify V5 (GKPIPNPLLGLDST) epitope-tagged APEX2 cDNA. mCherry with an N-terminal seven amino acid sequence (MGCVCSS) encoding the acylation sequence and APEX2 were then cloned into pcDNA™4/TO plasmid with zeocin selection (Invitrogen V1020-20). This plasmid is available in Addgene (#205089).

### Cells

A mouse A20 and human Raji B cell lines stably expressing a hen egg lysozyme (HEL)-specific IgM BCR (D1.3) (Aluvihare et al., 1997) were a kind gift from Prof. Facundo Batista (The Ragon Institute of MGH, MIT and Harvard, USA). A20 D1.3 cells were maintained in complete RPMI [cRPMI; RPMI 1640 with 2.05 mM L-glutamine supplemented with 10% fetal calf serum (FCS), 4 mM L-glutamine, 50 μM β-mercaptoethanol (#125472500, Acros Organics), 10 mM HEPES and 100 U/ml penicillin/streptomycin (Thermo Fisher Scientific)]. Raji D1.3 s were maintained in Raji cRPMI (RPMI 1640 with 2.05 mM L-glutamine supplemented with 10% FCS, 4 mM L-glutamine and 100 U/ml penicillin/streptomycin). The cell lines have been regularly (several times per year) checked for their identity by surface labeling of the transgenic BCR and kept mycoplasma free. Primary B cells were isolated from spleens of 2–3-month-old female or male C57Bl/6N mice, by negative selection with the EasySep™ Mouse B Cell Isolation Kit (#19854, STEMCELL Technologies). All animal experiments were performed according to approved guidelines from the Finnish Act on Animal Experimentation.

### Generation of the raft-APEX2-expressing stable cell line

Raft-APEX2-pcDNA™4/Zeo/TO plasmid was transfected into the A20 D1.3 cells line as previously described (Šuštar et al., 2018). In brief, 4×10^6^ cells were resuspended in 180 µl of 2S transfection buffer (5 mM KCl, 15 mM MgCl_2_, 15 mM HEPES, 50 mM sodium succinate, 180 mM Na_2_HPO_4_/ NaH_2_PO_4_ pH 7.2) containing 10 µg of plasmid DNA and electroporated using AMAXA electroporation machine (program X-005, Biosystem) in 0.2 cm gap electroporation cuvettes. Cells were then transferred to 4 ml of cRPMI containing an extra 10% FCS to recover overnight. Next, one single cell per well was sorted using Sony SH800 Cell Sorter into 96-well flat-bottom plates containing 100 µl of cRPMI containing 20% FCS. Cells were left to recover in cRPMI supplemented with an extra 10% FCS for 48 h before adding Zeocin (600 µg/ml final concentration). Clones expressing raft-APEX2 were selected and expanded few weeks after sorting. The expression of raft-APEX2 was verified with flow cytometry (see ‘Flow cytometry’ section below) analysis for mCherry and V5 (#R960-25, Life Technologies), and functional biotinylation was carried out as detailed below in the ‘Proximity biotinylation’ section and detected by means of Alexa Fluor^®^ (AF)633 streptavidin (1:1000; Life Technologies, S-21375). The expression level of D1.3 IgM was determined by flow cytometry using biotinylated anti-IgM (10 µg/ml; Southern Biotech, 1021-08) and AF633 streptavidin (1:1000; Life Technologies, S-21375).

### Proximity biotinylation

1×10^7^ A20 D1.3 raft-APEX2 cells in 5 ml of cRPMI were treated with 500 µM biotin-phenol (BP) (Iris-Biotech, CAS no. 41994-02-9) for 45 min and activated with 0 or 10 µg/ml of goat anti-mouse IgM F(ab′)_2_ fragments (Jackson ImmunoResearch 115-006-020) for 5, 10 or 15 min. 1 mM H_2_O_2_ (Sigma-Aldrich, cat. no. H1009-100ML) was added for 1 min and then quenched with 2× quenching solution (20 mM sodium ascorbate, 10 mM Trolox (Sigma-Aldrich, cat. no. 238813-1G) and 20 mM sodium azide solution in PBS). Cells were repeatedly washed four times with 1× quenching solution. Non-biotinylated control samples were prepared similarly but without anti-IgM and H_2_O_2._ Background control samples were prepared similarly but without BP and anti-IgM. To validate biotinylation for each experiment, we used flow cytometry, where cells were fixed, permeabilized and stained with streptavidin 633 (Thermo Fisher Scientific). Experiments for all conditions were performed in triplicate.

Samples were lysed with a modified RIPA buffer [50 mM Tris-HCl 150 mM NaCl, 0.1% SDS, 2% Octyl glucoside (Sigma-Aldrich, 29836-26-8), 0.5% sodium deoxycholate and 1% Triton X-100, pH 7.5] with 1× protease phosphatase inhibitor mini-tablet (1 tablet/10 ml, Thermo Fisher Scientific, cat. no. A32961). Lysate concentrations were measured using Pierce 660 nm protein assay (Thermo Fisher Scientific, cat. no. 22660), aliquoted into 360 µg of total protein/aliquot, snap-frozen and stored at −80°C.

### Streptavidin pulldown of biotinylated proteins

Whole-cell lysate (350 µg) was diluted in an additional 500 µl of RIPA buffer (50 mM Tris-HCl, 150 mM NaCl, 0.1% SDS, 0.5% sodium deoxycholate and 1% Triton X-100, pH 7.5, 1× protease phosphatase inhibitor mini-tablet) was incubated with 30 μl (0.3 mg) of streptavidin magnetic beads (Pierce, cat. no. 88817) for 1 h at RT on rotation. Beads were washed several times with 1 ml volumes for 5 min each, on ice, as follows: twice with RIPA buffer, twice with 1 M KCl, twice with 0.1 M Na_2_CO_3_, once with 4 M urea in 10 mM Tris-HCl pH 8.0, once with 50 µM biotin, 4 M urea in 10 mM Tris-HCl pH 8.0, and three times with RIPA buffer. Biotinylated proteins were eluted by boiling the beads in 30 μl of 3× SDS loading buffer (188 mM Tris-HCl pH 6.8, 3% SDS, 30% glycerol, 0.01% Bromophenol Blue) supplemented with 2 mM biotin and 20 mM DTT for 10 min.

### In-gel digestion

Eluted samples were run on 10% SDS-PAGE, and the gel was stained with SimplyBlue SafeStain (Thermo Fisher Scientific, cat. no. LC6065). For the digestion, a protocol adapted from Shevchenko et al. was used (Shevchenko et al., 2007). Each gel lane was cut into four pieces that were washed twice with 200 µl of 0.04 M NH_4_HCO_3_/50% acetonitrile (ACN) and dehydrated with 200 µl 100% ACN. Then, gel pieces were rehydrated in 200 µl of 20 mM DTT and dehydrated again as above. Gel pieces were then rehydrated with 100 μl 55 mM iodoacetamide for 20 min in the dark at room temperature, washed twice with 100 µl 100 mM NH_4_HCO_3_ and dehydrated as above. Finally, 30 µl of 0.02 µg/µl of trypsin (Promega V5111) solution was added to the gel pieces for 20 min followed by the addition of 60 μl solution containing 40 mM NH_4_HCO_3_/10% ACN to completely cover the gel pieces, and the samples were incubated at 37°C for 18 h. Peptides were extracted using 90 µl of ACN followed by 150 µl of 50% ACN/5% HCOOH at 37°C for 15 min.

### Mass spectrometry analysis

Data were collected by LC-ESI-MS/MS using a nanoflow HPLC system (Easy–nLC1200, Thermo Fisher Scientific) coupled to the Orbitrap Fusion Lumos mass spectrometer (Thermo Fisher Scientific, Bremen, Germany) equipped with a nano-electrospray ionization source. Peptides were first loaded on a trapping column and subsequently separated inline on a 15 cm C18 column. A linear 20 min gradient from 8 to 39% was used to elute peptides. MS data was acquired using Thermo Xcalibur 3.1 software (Thermo Fisher Scientific). A data-dependent acquisition method comprising an Orbitrap MS survey scan of mass range 300–2000 *m*/*z* followed by HCD fragmentation was used.

### Protein identification

The raw MS data were processed using MaxQuant software version 1.6.0.1 (Cox and Mann, 2008). MS/MS spectra were searched against mouse UniProt [reviewed (Swiss-Prot), released September 2019] using the Andromeda search engine (Cox et al., 2011). The following configuration was used for MaxQuant search: digestion set to trypsin, maximum number of missed cleavages allowed set to 2, fixed modification set to carbamidomethyl and variable modifications set to N-terminal acetylation and methionine oxidation. Peptide and protein false discovery rate was set to 0.01. Match between runs was enabled. MaxLFQ, which enables the determination of relative intensity values of proteins and normalizes proteins intensity between samples, was enabled but not used in downstream analysis (Cox et al., 2014). After MaxQuant run, 2526 proteins were identified, from which contaminants and reverse hits were removed. For further analysis, only the proteins identified with at least two unique peptides were considered as identified (1677 proteins). The identified proteins were then classified using both KEGG pathway analysis (Kanehisa et al., 2016) and GO classification (Ashburner et al., 2000). The GO group of ‘B cell activation’ was curated by adding BCR heavy and light chains as well as SH3KBP1. These data have been deposited to the ProteomeXchange Consortium via the PRIDE (Perez-Riverol et al., 2019) partner repository with the dataset identifier PXD025111.

### Proteomics and differential expression analysis

Normalization and differential expression analysis were undertaken using NormalyzerDE tools in Bioconductor (Willforss et al., 2019). Quantile normalization was selected as the best normalization method following a comparison of various normalization methods in NormalyzerDE (data not shown). Prior to normalization and differential expression analysis, identified proteins with missing values ≥7 out of 18 conditions were filtered out. For the remaining proteins, missing value imputation was undertaken using k-Nearest Neighbor (kNN) imputation. Differential expression analysis was undertaken using NormalyzerDE with statistical comparison method set to limma, logTrans set to FALSE, leastRepCount set to 1 and sigThresType set to FDR (Benjamini-Hochberg corrected *P*-values).

To identify likely raft-resident proteins, a strategy adapted from Paek et al. (2017) was used. We first selected proteins with ≥1.5 log_2_ fold-change in the non-activated biotinylated sample compared to control samples (sample without H_2_O_2_ triggered biotinylation). Then, proteins that showed a log_2_ foldchange ≥1 in the surrogate antigen-stimulated samples compared to non-activated samples were filtered out. The list of the proposed raft-resident proteins was compared with previously published mammalian lipid raft proteins available in the RaftProt database (https://raftprot.org/; Mohamed et al., 2019).

### Bioinformatics analysis

All downstream analysis were carried out with R (https://www.r-project.org/). Enhancedvolcano, which was used to generate volcano plots (https://bioconductor.org/packages/release/bioc/html/EnhancedVolcano.html). Enrichment analysis was done using the R clusterProfiler package (Yu et al., 2012). An UpSet plot was constructed to show the intersection between conditions. A Venn diagram was constructed, using DeepVenn, to depict the intersection of proteins significantly enriched upon BCR cross-linking at different time points. clValid, an R package in CRAN was used to determine the number of k-clusters prior to k-means analysis (Brock et al., 2008). The ComplexHeatmap R package was then used for k-means clustering of the differentially expressed proteins into k-clusters (Gu et al., 2016).

### Western blotting

Raft-APEX2 A20 D1.3 cells, or non-transfected A20 D1.3, were starved for 20 min in serum-free medium and incubated with either 10 µg/ml of goat anti-mouse IgM F(ab′)_2_ fragments (Jackson ImmunoResearch 115-006-020) for 10 min or 0.1, 1, 2, 5 and 10 mM H_2_O_2_ for 1 min. After which cells were lysed with 2× SDS PAGE loading buffer, sonicated and subjected to SDS PAGE. The blots were processed as described in Sarapulov et al. (2020) and probed with streptavidin–HRP (Life Technologies, #S-911).

For determining the effects on BCR signaling, the blots were probed for anti-phosphotyrosine (pTyr) antibody (1:1000; Merck Millipore, #05-321), or anti-pSyk (Y319)/pZap-70 (Y352) (1:1000; Cell Signaling Technologies, #2701) and anti-Syk (1:1000, D3Z1E) (Cell Signaling Technologies, #13198S) at +4°C, overnight. Blots were stripped with 25 mM glycine-HCl buffer, 1% SDS, pH ∼2.5, for 15 min, blocked, and reprobed with anti-β-actin antibody (HRP) (1:20,000; AC-15, Abcam, #ab49900). For quantification, images were background subtracted and integrated densities of the lanes (pTyr) or specific bands (p-Syk and Syk) were normalized to corresponding total β-actin densities. Highest intensity signal was defined as 100% and the highest background (empty lane) intensity signal as 0%. Quantification was performed with Fiji ImageJ (NIH). TAK981-treated samples, H_2_O_2_ titration and total Syk and Syk phosphorylation were analyzed by repeated measures two-way ANOVA with Šídák multiple comparisons test, one-way ANOVA with Šídák multiple comparisons test, and paired two-tailed *t*-test, respectively, in GraphPad Prism 9.

For the analysis of BCR signaling in TAK981-treated B cells, A20 D1.3 or primary splenic B cells were transferred to plain RPMI, incubated with 25 µM TAK981 or DMSO as a control for 30 min, and then stimulated with soluble or pre-adsorbed plate-bound (both at 10 µg/ml) goat anti-mouse IgM F(ab′)_2_ fragments (Jackson ImmunoResearch 115-006-020) for 15 min in the presence of TAK981 inhibitor and lysed by the addition of 5× SDS lysis buffer to the final concentration of 2% SDS and subjected to immunoblotting as above. Briefly, the blots were probed with antibodies against phosphorylated forms of proteins, stripped and reprobed with antibodies against total corresponding proteins. Following antibodies, all at 1:1000 dilution, were used: anti-pSyk (Y319) and pZap-70 (Y352) (#2701), anti-Syk (#13198), anti-pAkt (S473) (#4058), anti-Akt (#2938), anti-pERK1/2 (p44/p42; T202/Y204) (#9101), and anti-ERK1/2 (#9102), and pAkt-substrate (#9614), all from Cell Signaling Technology. Inhibition of SUMOylation was confirmed with anti-Sumo1 antibody (Y299) (ab32058, Abcam). Images were quantified as above and phospho-protein densities normalized to the corresponding total protein densities. Integrated densities of pAkt-substrate signal were quantified in ImageJ from images after histogram normalization using ‘Enhance contrast...’ with 0.001% saturated pixels.

Images of the original uncropped blots are shown in the Fig. S7.

### Analysis of membrane detergent resistance by flow cytometry

A20D1.3 cells were electroporated with raft-APEX2, caveolin-1-RFP as a raft marker, or GFP fused to influenza virus hemagglutinin transmembrane domain (TDM) (Nikolaus et al., 2010) as a non-raft marker. The assay was performed according to Gombos et al. (2004). In short, at 24 h after transfection, every condition was divided into two tubes. Cells (10^6^/ml in imaging buffer; PBS with 10% FCS) were kept on ice, and Triton X-100 was added to a final concentration of 0.1% to one of the tubes, whereas the other tube was left untreated, and fluorescence was immediately recorded in a flow cytometer. The parameter of detergent resistance was calculated as DRI=(FL_det_-FLBg_det_)/(FL_max_-FLBg), where FL_det_ stands for fluorescence of the cells treated with detergent for 5 min, FLBg_det_ for autofluorescence of the detergent–treated cells, FL_max_ for fluorescence of labeled untreated cells (proportional to the protein expression level), FLBg for autofluorescence (background) of the unlabeled cells. The experiment was repeated at least six times for each marker.

### AiryScan confocal microscopy to analyze raft-APEX2 localization

Glass-bottom microscopy chambers (MatTek) were coated with 4 µg/ml fibronectin in PBS for 1 h at room temperature and washed with PBS. Similar to what was undertaken for the mass spectrometry samples, cells were incubated in 500 µM BP in complete medium at 37°C for 45 min. The cells were then let to settle on the microscopy chambers at 37°C for 15 min and incubated with 1 mM H_2_O_2_ together with 4% paraformaldehyde and 0.1% glutaraldehyde to immediately fix the sample, washed and allowed to continue to fix for a further 10 min. Next, cells were washed, blocked in blocking buffer (0.5% BSA+0.5% goat serum) at room temperature for 1 h, labeled with Atto488-labeled WGA, washed, permeabilized with 0.1% Triton X-100, at room temperature for 5 min, blocked again, and labeled with streptavidin–AF633 (1:2000) and DAPI at room temperature for 1 h. After washing, the samples were mounted in Vectashield. For visualization of raft-APEX2 upon BCR activation, after settling, 1 µg/ml of HEL antigen was added on ice, then incubated at 37°C for 5 min and fixed for processing as above with the exception of staining with Atto488-labeled goat anti-mouse IgM F(ab′)_2_ fragments (Jackson ImmunoResearch 115-006-020) instead of WGA–Atto488. Images were acquired using a laser scanning confocal microscope LSM880 (Zeiss) with an Airyscan detector (32-channel Airyscan Hexagonal element) equipped with 405 (Diode), 488 (Argon) and 633 (HeNe) laser lines and an oil-immersion 63× Zeiss Plan-Apochromat objective. Images were acquired using the standard super-resolution mode (Zen Black 2.3). The profile intensity analysis was done in Fiji ImageJ (NIH) (Schindelin et al., 2012).

### Immunofluorescence sample preparation

Unless otherwise stated, 12-well slides (Thermo Fisher Scientific, ER-202-CE24) were coated with 4 µg/ml fibronectin (non-activatory ligand) in PBS for 20 min at room temperature. A20 D1.3 or primary B cells isolated from C57BL/6 mice spleens were seeded on the fibronectin-coated wells and incubated at 37°C for at least 15 min to allow adhesion. Then, cells were fixed with 4% PFA for 10 min at room temperature, and blocked and permeabilized for 20 min at room temperature (5% donkey serum with 0.3% Triton X-100 in PBS). After blocking, samples were stained with primary antibodies for 1 h at room temperature or 4°C overnight in staining buffer (1% BSA and 0.3% Triton X-100 in PBS), followed by washes with PBS and incubation with the fluorescently labeled secondary antibodies for 30 min at room temperature in PBS. For immunostaining, anti-Sumo1 (Y299, Abcam, ab32058) was used at a dilution 1:500, anti-PCM1 (Santa Cruz Biotechnologies, sc-398365 AF647) at 1:400, donkey anti-mouse IgM F(ab′)_2_–RRx (Jackson ImmunoResearch, #715-296-020) at 1:500 to stain the IgM BCR, and anti-phosphotyrosine (4G10, Merck Millipore, 05-321) at 1:400. Secondary antibodies anti-rabbit IgG conjugated to AF488 (Thermo Fisher Scientific, A-21206) and anti-mouse IgG2b conjugated to AF633 (Thermo Fisher Scientific, A-21146) were used at 1:500. For actin staining, phalloidin 555 (Acti-stain 555 Cytoskeleton, #PHDH1-A) at 1:400 dilution was used. Samples were mounted using FluoroMount-G-containing DAPI (Thermo Fisher Scientific, #00495952). Images were acquired on a 3i CSU-W1 Marianas spinning disk confocal microscope (Intelligent Imaging Innovations) equipped with a 63× Zeiss Plan-Apochromat objective (NA 1.4) and a Hamamatsu sCMOS Orca Flash4.0 camera (2048×2048 pixels, 1×1 binning).

For experiments where activation with soluble antigen was needed, A20 D1.3 or primary B cells were labeled on ice for 10 min with 10 µg/ml of AF647-conjugated donkey anti-mouse IgM (#715-605-140, Jackson ImmunoResearch) or RRx-conjugated goat anti-mouse IgM (#115-295-205, Jackson ImmunoResearch), washed with PBS to remove excess unbound antigen and resuspended in imaging buffer (PBS with 10% FCS). Cells were then seeded on the fibronectin-coated slides for 5 to 30 min to allow activation and samples were processed as described above. For experiments where activation with surface-bound antigen was used, 12-well slides were coated with 10 µg/ml F(ab’)₂ fragment goat anti-mouse IgM (Jackson ImmunoResearch, 115-006-020) in PBS for 1 h at room temperature or fibronectin as a control, as described above. Cells were seeded on the coated slides, activated for 5–15 min, and processed as described above. The profile intensity analysis was done in Fiji ImageJ (NIH) (Schindelin et al., 2012).

For experiments where cells were activated with antigen-coated beads, 5 µm Streptavidin beads (Bangs Laboratories, #CP01N/10984) were coated with 10 µg/ml of biotinylated goat anti-mouse IgM at 37°C for 30 min (shaking at 1000 rpm) and washed in 2% BSA in PBS. Uncoated beads were used as a negative control. Cells were mixed with the beads (1:1) and plated on the fibronectin-coated wells for 30 min (+37°C, 5% CO_2_) to trigger activation. Samples were then processed and imaged as described above.

In experiments where the SUMO1 inhibitor was used, A20 D1.3 cells were suspended in imaging buffer and pretreated with the SUMOylation inhibitor TAK981 (MedChemExpress, HY-111789) at a 25 µM final concentration or DMSO as a control in the incubator (5% CO_2_, +37°C). After that they were seeded in the 12-well slides coated with 10 µg/ml F(ab’)₂ fragment goat anti-mouse IgM (Jackson ImmunoResearch, 115-006-020) in PBS for 1 h at room temperature and activated for 15 or 5 min in the incubator, being treated with the inhibitor for 45 min in total, and processed as above. Samples were imaged as above and, using Fiji ImageJ, the cell area was thesholded, based on the phalloidin channel, for the analysis of area and signal intensity. Data were analyzed by a paired two-tailed *t*-test on the mean values of each experiment.

### Immunofluorescence sample preparation, acquisition and image analysis for Golga3

Eight-well polymer coverslips (µ-Slide 8, high-well, IBIDI 80806) were coated with CellTak substrate (CellTak, Corning®, purchased from Sigma-Aldrich DLW354242) according to the manufacturer's recommendations. In short, 80 µl of 56 µg/ml CellTak in H_2_O was applied in each well. CellTak was topped with 120 µl of 0.1 M NaHCO_3_ pH 8.0. to activate the reaction. The slides were incubated for 1–2 h at room temperature, washed once with H_2_O, dried and stored at +4°C. 150,000 Raji D1.3 cells that were either non activated or activated with 5 µg/ml of HEL (Sigma-Aldrich, 10837059001) or 10 µg/ml AF488 or AF647 labeled F(ab’)₂ fragments of donkey anti-mouse IgM (Jackson ImmunoResearch 715-546-020 and 715-606-020), were placed in 300 µl of imaging buffer (10% FCS in PBS) on coverslip wells and left in the incubator (5% CO_2_, +37°C) for 15 min. The cells were fixed with 50:50 methanol-acetone at −20°C for 20 min, permeabilized with acetone for 5 min at −20°C and blocked (5% donkey serum in PBS) for 1–2 h at room temperature. The cells were stained with primary antibodies in PBS supplemented with 5% BSA overnight in +4°C, and processed further as above.

For immunostainings, anti-α-tubulin AF488 or -647 (DM1A, Merc Millipore, Sigma-Aldrich 16-232 / 05-829-AF647) was used at the dilution of 1:150 and anti-Golga3 (Sigma-Aldrich HPA040044) was used at 1:150. Secondary anti-mouse IgG1 conjugated to AF488 or AF647 (Jackson ImmunoResearch 115-545-205 and 115-605-205) and anti-rabbit IgG conjugated to AF555 (Invitrogen A-31572) were used at 1:500. Images were deconvoluted with Huygens Essential version 16.10 (Scientific Volume Imaging, The Netherlands, http://svi.nl), using automated algorithms and thresholded optimally, yet consistently. Particle analysis was performed with Huygens Essential. In Golga3 particle analysis, intensity values of <5% of the maximum were considered as background. In Golga3 particle analysis, the tubulin channel was used to define cell outlines in 3D that were then shrank by 0.27 µm, corresponding to 1 pixel along the *z*-axis. Intensities inside the 3D selection were cleared, resulting in the remaining hollow sphere comprising the cell periphery only. Peripheral Golga3 particles were then automatically analyzed. Colocalization between Golga3 and antigen was analyzed with Huygens Essential, also from the peripheral spheres, using optimized, automatic thresholding.

### BCR internalization and flow cytometry

To study the BCR internalization upon activation with surrogate antigen, Raft-APEX2 A20 D1.3 cells, or non-transfected A20 D1.3 cells, were stained for 5 min on ice with 10 μg/ml of biotinylated anti-IgM (Southern Biotech, 1021-08) and washed with imaging buffer. Cells (10^5^/well, 96-wp) were then incubated at 37°C and 5% CO_2_ for 60, 45, 30, 15 and 5 min. As a control (time 0), samples were always kept on ice. After incubation, cells were stained with AF633–streptavidin (1:1000; Life Technologies, S-21375) in PBS on ice for 20 min. Samples were then washed with cold PBS and analyzed. The internalization rate for the biotinylated anti-IgM samples was calculated as described previously (Hernández-Pérez and Mattila, 2022). A BD LSR Fortessa analyser equipped with four lasers (405, 488, 561, and 640 nm) was used. Data were analyzed using FlowJo v10 (Tree Star).

### Statistics and illustrations

Graphs and statistics were prepared with GraphPad Prism (GraphPad Software, La Jolla, CA, USA). Statistical significances were calculated using an unpaired or paired two-tailed Student's *t*-test assuming a normal distribution of the data, or repeated measures two-way ANOVA. Statistical values are denoted as: *P<0.05, **P<0.01, ***P<0.001, ****P<0.0001. Illustrations were created with BioRender. Figure formatting was undertaken in Inkscape v. 092.2.

## Supplementary Material

10.1242/joces.261119_sup1 Supplementary information

## References

[JCS261119C1] Aluvihare, V. R., Khamlichi, A. A., Williams, G. T., Adorini, L. and Neuberger, M. S. (1997). Acceleration of intracellular targeting of antigen by the B-cell antigen receptor: importance depends on the nature of the antigen-antibody interaction. *EMBO J.* 16, 3553-3562. 10.1093/emboj/16.12.3553 9218797 PMC1169980

[JCS261119C2] Aman, M. J. and Ravichandran, K. S. (2000). A requirement for lipid rafts in B cell receptor induced Ca(2+) flux. *Curr. Biol.* 10, 393-396. 10.1016/S0960-9822(00)00415-2 10753749

[JCS261119C3] Ashburner, M., Ball, C. A., Blake, J. A., Botstein, D., Butler, H., Cherry, J. M., Davis, A. P., Dolinski, K., Eppig, J. T., Harris, M. A., et al. (2000). Gene ontology: tool for the unification of biology. *Nat. Genetics* 25, 25-29. 10.1038/75556 10802651 PMC3037419

[JCS261119C4] Bareja, A., Hodgkinson, C. P., Soderblom, E., Waitt, G. and Dzau, V. J. (2018). The proximity-labeling technique BioID identifies sorting nexin 6 as a member of the insulin-like growth factor 1 (IGF1)–IGF1 receptor pathway. *J. Biol. Chem.* 293, 6449-6459. 10.1074/jbc.RA118.002406 29530981 PMC5925803

[JCS261119C5] Bécart, S., Canonigo Balancio, A. J., Charvet, C., Feau, S., Sedwick, C. E. and Altman, A. (2008). Tyrosine-phosphorylation-dependent translocation of the SLAT protein to the immunological synapse is required for NFAT transcription factor activation. *Immunity* 29, 704-719. 10.1016/j.immuni.2008.08.015 18976935 PMC2825161

[JCS261119C6] Bi, K., Tanaka, Y., Coudronniere, N., Sugie, K., Hong, S., Van Stipdonk, M. J. and Altman, A. (2001). Antigen-induced translocation of PKC-theta to membrane rafts is required for T cell activation. *Nat. Immunol.* 2, 556-563. 10.1038/88765 11376344

[JCS261119C8] Bosch, J. A., Chen, C. and Perrimon, N. (2021). Proximity–dependent labeling methods for proteomic profiling in living cells: an update. *WIREs Dev. Biol.* 10, e392. 10.1002/wdev.392 PMC8142282 32909689

[JCS261119C9] Brock, G., Pihur, V., Datta, S. and Datta, S. (2008). clValid: an R package for cluster validation. *J. Stat. Softw.* 25, 1-22. 10.18637/jss.v025.i04

[JCS261119C10] Brunet, A., Bonni, A., Zigmond, M. J., Lin, M. Z., Juo, P., Hu, L. S., Anderson, M. J., Arden, K. C. and Greenberg, M. E. (1999). Akt promotes cell survival by phosphorylating and inhibiting a Forkhead transcription factor. *Cell* 96, 857-868. 10.1016/S0092-8674(00)80595-4 10102273

[JCS261119C11] Chang, H.-M. and Yeh, E. T. H. (2020). SUMO: from bench to bedside. *Physiol. Rev.* 100, 1599-1619. 10.1152/physrev.00025.2019 32666886 PMC7717128

[JCS261119C12] Chen, C.-L. and Perrimon, N. (2017). Proximity-dependent labeling methods for proteomic profiling in living cells. *Wiley Interdiscip. Rev. Dev. Biol.* 6, e272. 10.1002/wdev.272 PMC5553119 28387482

[JCS261119C13] Cheng, P. C., Dykstra, M. L., Mitchell, R. N. and Pierce, S. K. (1999). A role for lipid rafts in B cell antigen receptor signaling and antigen targeting. *J. Exp. Med.* 190, 1549-1560. 10.1084/jem.190.11.1549 10587346 PMC2195743

[JCS261119C14] Courtois, G. and Fauvarque, M.-O. (2018). The many roles of ubiquitin in NF-κB signaling. *Biomedicines* 6, 43. 10.3390/biomedicines6020043 29642643 PMC6027159

[JCS261119C15] Cox, J. and Mann, M. (2008). MaxQuant enables high peptide identification rates, individualized p.p.b.-range mass accuracies and proteome-wide protein quantification. *Nat. Biotechnol.* 26, 1367-1372. 10.1038/nbt.1511 19029910

[JCS261119C16] Cox, J., Neuhauser, N., Michalski, A., Scheltema, R. A., Olsen, J. V. and Mann, M. (2011). Andromeda: a peptide search engine integrated into the MaxQuant environment. *J. Proteome Res.* 10, 1794-1805. 10.1021/pr101065j 21254760

[JCS261119C17] Cox, J., Hein, M. Y., Luber, C. A., Paron, I., Nagaraj, N. and Mann, M. (2014). Accurate proteome-wide label-free quantification by delayed normalization and maximal peptide ratio extraction, termed MaxLFQ. *Mol. Cell. Proteomics* 13, 2513-2526. 10.1074/mcp.M113.031591 24942700 PMC4159666

[JCS261119C18] Cunha, D. M., Hernández-Pérez, S. and Mattila, P. K. (2023a). Isolation of the B cell immune synapse for proteomic analysis. *Methods Mol. Biol.* 2654, 393-408. 10.1007/978-1-0716-3135-5_25 37106196

[JCS261119C19] Cunha, D. M., Hernández-Pérez, S., Awoniyi, L. O., Carisey, A. F., Jacquemet, G. and Mattila, P. K. (2023b). Proteomic profiling of isolated immune synapses from primary mouse B cells. *bioRxiv* 2023.02.23.529674. 10.1101/2023.02.23.529674

[JCS261119C20] Depoil, D., Fleire, S., Treanor, B. L., Weber, M., Harwood, N. E., Marchbank, K. L., Tybulewicz, V. L. J. and Batista, F. D. (2008). CD19 is essential for B cell activation by promoting B cell receptor-antigen microcluster formation in response to membrane-bound ligand. *Nat. Immunol.* 9, 63-72. 10.1038/ni1547 18059271

[JCS261119C21] Doerr, A. (2018). Proximity labeling with TurboID. *Nat. Methods* 15, 764. 10.1038/s41592-018-0158-0 30275580

[JCS261119C22] Dumin, E., Bendikov, I., Foltyn, V. N., Misumi, Y., Ikehara, Y., Kartvelishvily, E. and Wolosker, H. (2006). Modulation of D-serine levels via ubiquitin-dependent proteasomal degradation of serine racemase. *J. Biol. Chem.* 281, 20291-20302. 10.1074/jbc.M601971200 16714286

[JCS261119C23] Gombos, I., Bacsó, Z., Detre, C., Nagy, H., Goda, K., Andrásfalvy, M., Szabó, G. and Matkó, J. (2004). Cholesterol sensitivity of detergent resistance: a rapid flow cytometric test for detecting constitutive or induced raft association of membrane proteins. *Cytom. Part J. Int. Soc. Anal. Cytol.* 61, 117-126. 10.1002/cyto.a.20080 15382146

[JCS261119C24] Gu, Z., Eils, R. and Schlesner, M. (2016). Complex heatmaps reveal patterns and correlations in multidimensional genomic data. *Bioinforma.* 32, 2847-2849. 10.1093/bioinformatics/btw313 27207943

[JCS261119C25] Gupta, N. and Defranco, A. L. (2003). Visualizing lipid raft dynamics and early signaling events during antigen receptor-mediated B-lymphocyte activation. *Mol. Biol. Cell* 14, 432-444. 10.1091/mbc.02-05-0078 12589045 PMC149983

[JCS261119C26] Gupta, N., Wollscheid, B., Watts, J. D., Scheer, B., Aebersold, R. and Defranco, A. L. (2006). Quantitative proteomic analysis of B cell lipid rafts reveals that ezrin regulates antigen receptor-mediated lipid raft dynamics. *Nat. Immunol.* 7, 625-633. 10.1038/ni1337 16648854

[JCS261119C27] Hao, J.-J., Carey, G. B. and Zhan, X. (2004). Syk-mediated tyrosine phosphorylation is required for the association of hematopoietic lineage cell-specific protein 1 with lipid rafts and B cell antigen receptor signalosome complex. *J. Biol. Chem.* 279, 33413-33420. 10.1074/jbc.M313564200 15166239

[JCS261119C28] Hartigan, J. A. and Wong, M. A. (1979). A K-means clustering algorithm. *J. R. Stat. Soc. Ser. C Appl. Stat.* 28, 100-108.

[JCS261119C29] Harwood, N. E. and Batista, F. D. (2010). Early events in B cell activation. *Annu. Rev. Immunol.* 28, 185-210. 10.1146/annurev-immunol-030409-101216 20192804

[JCS261119C30] He, Y., Yang, Z., Zhao, C., Xiao, Z., Gong, Y., Li, Y.-Y., Chen, Y., Du, Y., Feng, D., Li, Y. et al. (2021). T-cell receptor (TCR) signaling promotes the assembly of RanBP2/RanGAP1-SUMO1/Ubc9 nuclear pore subcomplex via PKC-θ-mediated phosphorylation of RanGAP1. *ELife* 10, e67123. 10.7554/eLife.67123 34110283 PMC8225385

[JCS261119C31] Hernández-Pérez, S. and Mattila, P. K. (2022). A specific hybridisation internalisation probe (SHIP) enables precise live-cell and super-resolution imaging of internalized cargo. *Sci. Rep.* 12, 620. 10.1038/s41598-021-04544-6 35022457 PMC8755761

[JCS261119C32] Hernández-Pérez, S., Vainio, M., Kuokkanen, E., Šuštar, V., Petrov, P., Forstén, S., Paavola, V., Rajala, J., Awoniyi, L. O., Sarapulov, A. V. et al. (2020). B cells rapidly target antigen and surface-derived MHCII into peripheral degradative compartments. *J. Cell Sci.* 133, jcs235192. 10.1242/jcs.235192 31780582

[JCS261119C33] Hicks, S. W. and Machamer, C. E. (2005). Isoform-specific interaction of golgin-160 with the Golgi-associated protein PIST. *J. Biol. Chem.* 280, 28944-28951. 10.1074/jbc.M504937200 15951434

[JCS261119C34] Hicks, S. W., Horn, T. A., Mccaffery, J. M., Zuckerman, D. M. and Machamer, C. E. (2006). Golgin-160 promotes cell surface expression of the beta-1 adrenergic receptor. *Traffic* 7, 1666-1677. 10.1111/j.1600-0854.2006.00504.x 17118120

[JCS261119C35] Hou, P., Araujo, E., Zhao, T., Zhang, M., Massenburg, D., Veselits, M., Doyle, C., Dinner, A. R. and Clark, M. R. (2006). B cell antigen receptor signaling and internalization are mutually exclusive events. *PLoS Biol.* 4, 1147-1158. 10.1371/journal.pbio.0040200 PMC1470458 16719564

[JCS261119C36] Huang, D. W., Sherman, B. T. and Lempicki, R. A. (2009a). Bioinformatics enrichment tools: paths toward the comprehensive functional analysis of large gene lists. *Nucleic Acids Res.* 37, 1-13. 10.1093/nar/gkn923 19033363 PMC2615629

[JCS261119C37] Huang, D. W., Sherman, B. T. and Lempicki, R. A. (2009b). Systematic and integrative analysis of large gene lists using DAVID bioinformatics resources. *Nat. Protoc.* 4, 44-57. 10.1038/nprot.2008.211 19131956

[JCS261119C38] Huff, J. (2015). The Airyscan detector from ZEISS: confocal imaging with improved signal-to-noise ratio and super-resolution. *Nat. Methods* 12, i-ii. 10.1038/nmeth.f.388

[JCS261119C39] Hung, V., Zou, P., Rhee, H.-W., Udeshi, N. D., Cracan, V., Svinkina, T., Carr, S. A., Mootha, V. K. and Ting, A. Y. (2014). Proteomic mapping of the human mitochondrial intermembrane space in live cells via ratiometric APEX tagging. *Mol. Cell* 55, 332-341. 10.1016/j.molcel.2014.06.003 25002142 PMC4743503

[JCS261119C40] Hung, V., Udeshi, N. D., Lam, S. S., Loh, K. H., Cox, K. J., Pedram, K., Carr, S. A. and Ting, A. Y. (2016). Spatially resolved proteomic mapping in living cells with the engineered peroxidase APEX2. *Nat. Protoc.* 11, 456-475. 10.1038/nprot.2016.018 26866790 PMC4863649

[JCS261119C41] Jiao, X., Sherman, B. T., Huang, D. W., Stephens, R., Baseler, M. W., Lane, H. C. and Lempicki, R. A. (2012). DAVID-WS: a stateful web service to facilitate gene/protein list analysis. *Bioinformatics* 28, 1805-1806. 10.1093/bioinformatics/bts251 22543366 PMC3381967

[JCS261119C42] Kanehisa, M., Sato, Y., Kawashima, M., Furumichi, M. and Tanabe, M. (2016). KEGG as a reference resource for gene and protein annotation. *Nucleic Acids Res.* 44, D457-D462. 10.1093/nar/gkv1070 26476454 PMC4702792

[JCS261119C43] Kuokkanen, E., Šuštar, V. and Mattila, P. K. (2015). Molecular control of B cell activation and immunological synapse formation. *Traffic Cph. Den.* 16, 311-326. 10.1111/tra.12257 25639463

[JCS261119C44] Kwak, K., Akkaya, M. and Pierce, S. K. (2019). B cell signaling in context. *Nat. Immunol.* 20, 963-969. 10.1038/s41590-019-0427-9 31285625

[JCS261119C45] Lam, S. S., Martell, J. D., Kamer, K. J., Deerinck, T. J., Ellisman, M. H., Mootha, V. K. and Ting, A. Y. (2015). Directed evolution of APEX2 for electron microscopy and proximity labeling. *Nat. Methods* 12, 51-54. 10.1038/nmeth.3179 25419960 PMC4296904

[JCS261119C46] Langston, S. P., Grossman, S., England, D., Afroze, R., Bence, N., Bowman, D., Bump, N., Chau, R., Chuang, B.-C., Claiborne, C., et al. (2021). Discovery of TAK-981, a first-in-class inhibitor of SUMO-activating enzyme for the treatment of cancer. *J. Med. Chem.* 64, 2501-2520. 10.1021/acs.jmedchem.0c01491 33631934

[JCS261119C47] Li, X. W., Rees, J. S., Xue, P., Zhang, H., Hamaia, S. W., Sanderson, B., Funk, P. E., Farndale, R. W., Perrett, S., Jackson, A. P. et al. (2014). New insights into the DT40 B cell receptor cluster using a proteomic proximity labeling assay. *J. Biol. Chem.* 289, 14434-14447. 10.1074/jbc.M113.529578 24706754 PMC4031500

[JCS261119C48] Liem, R. K. H. (2016). Cytoskeletal integrators: the spectrin superfamily. *Cold Spring Harbor Perspect. Biol.* 8, a018259. 10.1101/cshperspect.a018259 PMC5046693 27698030

[JCS261119C49] Lightcap, E. S., Yu, P., Grossman, S., Song, K., Khattar, M., Xega, K., He, X., Gavin, J. M., Imaichi, H., Garnsey, J. J., et al. (2021). A small-molecule SUMOylation inhibitor activates antitumor immune responses and potentiates immune therapies in preclinical models. *Sci. Transl. Med.* 13, eaba7791. 10.1126/scitranslmed.aba7791 34524860 PMC9719791

[JCS261119C50] Maag, R. S., Mancini, M., Rosen, A. and Machamer, C. E. (2005). Caspase-resistant golgin-160 disrupts apoptosis induced by secretory pathway stress and ligation of death receptors. *Mol. Biol. Cell* 16, 3019-3027. 10.1091/mbc.e04-11-0971 15829563 PMC1142444

[JCS261119C51] Martin, B. R. and Cravatt, B. F. (2009). Large-scale profiling of protein palmitoylation in mammalian cells. *Nat. Methods* 6, 135-138. 10.1038/nmeth.1293 19137006 PMC2775068

[JCS261119C52] Martinez-Outschoorn, U. E., Sotgia, F. and Lisanti, M. P. (2015). Caveolae and signalling in cancer. *Nature Reviews Cancer* 15, 225-237. 10.1038/nrc3915 25801618

[JCS261119C53] Matsumoto, M., Oyamada, K., Takahashi, H., Sato, T., Hatakeyama, S. and Nakayama, K. I. (2009). Large-scale proteomic analysis of tyrosine-phosphorylation induced by T-cell receptor or B-cell receptor activation reveals new signaling pathways. *Proteomics* 9, 3549-3563. 10.1002/pmic.200900011 19609962

[JCS261119C54] Mattila, P. K., Feest, C., Depoil, D., Treanor, B., Montaner, B., Otipoby, K. L., Carter, R., Justement, L. B., Bruckbauer, A. and Batista, F. D. (2013). The actin and tetraspanin networks organize receptor nanoclusters to regulate B cell receptor-mediated signaling. *Immunity* 38, 461-474. 10.1016/j.immuni.2012.11.019 23499492

[JCS261119C55] Mattila, P. K., Batista, F. D. and Treanor, B. (2016). Dynamics of the actin cytoskeleton mediates receptor cross talk: an emerging concept in tuning receptor signaling. *J. Cell Biol.* 212, 267-280. 10.1083/jcb.201504137 26833785 PMC4748574

[JCS261119C56] Mick, D. U., Rodrigues, R. B., Leib, R. D., Adams, C. M., Chien, A. S., Gygi, S. P. and Nachury, M. V. (2015). Proteomics of primary cilia by proximity labeling. *Dev. Cell* 35, 497-512. 10.1016/j.devcel.2015.10.015 26585297 PMC4662609

[JCS261119C57] Mielenz, D., Vettermann, C., Hampel, M., Lang, C., Avramidou, A., Karas, M. and Jäck, H.-M. (2005). Lipid rafts associate with intracellular B cell receptors and exhibit a B cell stage-specific protein composition. *J. Immunol.* 174, 3508-3517. 10.4049/jimmunol.174.6.3508 15749887

[JCS261119C58] Mohamed, A., Shah, A. D., Chen, D. and Hill, M. M. (2019). RaftProt V2: understanding membrane microdomain function through lipid raft proteomes. *Nucleic Acids Res.* 47, D459-D463. 10.1093/nar/gky948 30329070 PMC6323919

[JCS261119C59] Mollinedo, F. and Gajate, C. (2020). Lipid rafts as signaling hubs in cancer cell survival/death and invasion: implications in tumor progression and therapy. *J. Lipid Res.* 61, 611-635. 10.1194/jlr.TR119000439 33715811 PMC7193951

[JCS261119C60] Music, A., Tejeda-González, B., Cunha, D. M., Fischer Von Mollard, G., Hernández-Pérez, S. and Mattila, P. K. (2022). The SNARE protein Vti1b is recruited to the sites of BCR activation but is redundant for antigen internalisation, processing and presentation. *Front. Cell Dev. Biol.* 10, 987148. 10.3389/fcell.2022.987148 36111340 PMC9468668

[JCS261119C61] Negishi, H., Ohba, Y., Yanai, H., Takaoka, A., Honma, K., Yui, K., Matsuyama, T. and Taniguchi, T. (2005). Negative regulation of Toll-like-receptor signaling by IRF-4. *Proc. Natl. Acad. Sci. USA* 102, 15989-15994. 10.1073/pnas.0508327102 16236719 PMC1257749

[JCS261119C62] Nikolaus, J., Scolari, S., Bayraktarov, E., Jungnick, N., Engel, S., Plazzo, A. P., Stöckl, M., Volkmer, R., Veit, M. and Herrmann, A. (2010). Hemagglutinin of influenza virus partitions into the nonraft domain of model membranes. *Biophys. J.* 99, 489-498. 10.1016/j.bpj.2010.04.027 20643067 PMC2905074

[JCS261119C63] Nishiyama, A., Ryo, A., Ban, T., Sato, G. R., Nishiyama, A., Akiyama, A., Takasuna, M., Umehara, M., Suzuki, S., Kimura, A. et al. (2016). Lyn kinase suppresses the transcriptional activity of IRF5 in the TLR-MyD88 pathway to restrain the development of autoimmunity article Lyn kinase suppresses the transcriptional activity of IRF5 in the TLR-MyD88 pathway to restrain the development of auto. *Immunity* 45, 319-332. 10.1016/j.immuni.2016.07.015 27521268

[JCS261119C64] Okamura, M., Inose, H. and Masuda, S. (2015). RNA export through the NPC in Eukaryotes. *Genes* 6, 124-149. 10.3390/genes6010124 25802992 PMC4377836

[JCS261119C65] Paek, J., Kalocsay, M., Staus, D. P., Wingler, L., Pascolutti, R., Paulo, J. A., Gygi, S. P. and Kruse, A. C. (2017). Multidimensional tracking of GPCR signaling via peroxidase-catalyzed proximity labeling. *Cell* 169, 338-349.e11. 10.1016/j.cell.2017.03.028 28388415 PMC5514552

[JCS261119C66] Perez-Riverol, Y., Csordas, A., Bai, J., Bernal-Llinares, M., Hewapathirana, S., Kundu, D. J., Inuganti, A., Griss, J., Mayer, G., Eisenacher, M., et al. (2019). The PRIDE database and related tools and resources in 2019: improving support for quantification data. *Nucleic Acids Res.* 47, D442-D450. 10.1093/nar/gky1106 30395289 PMC6323896

[JCS261119C67] Qin, W., Cho, K. F., Cavanagh, P. E. and Ting, A. Y. (2021). Deciphering molecular interactions by proximity labeling. *Nat. Methods* 18, 133-143. 10.1038/s41592-020-01010-5 33432242 PMC10548357

[JCS261119C68] Randall, K. L., Lambe, T., Goodnow, C. C. and Cornall, R. J. (2010). The essential role of DOCK8 in humoral immunity. *Dis. Markers* 29, 141-150. 10.1155/2010/143612 21178273 PMC3835290

[JCS261119C69] Razinia, Z., Mäkelä, T., Ylänne, J. and Calderwood, D. A. (2012). Filamins in mechanosensing and signaling. *Annu. Rev. Biophys.* 41, 227-246. 10.1146/annurev-biophys-050511-102252 22404683 PMC5508560

[JCS261119C70] Reth, M. (2002). Hydrogen peroxide as second messenger in lymphocyte activation. *Nat. Immunol.* 3, 1129-1134. 10.1038/ni1202-1129 12447370

[JCS261119C71] Rhee, H.-W., Zou, P., Udeshi, N. D., Martell, J. D., Mootha, V. K., Carr, S. A. and Ting, A. Y. (2013). Proteomic mapping of mitochondria in living cells via spatially restricted enzymatic tagging. *Science* 339, 1328-1331. 10.1126/science.1230593 23371551 PMC3916822

[JCS261119C72] Roux, K. J., Kim, D. I., Raida, M. and Burke, B. (2012). A promiscuous biotin ligase fusion protein identifies proximal and interacting proteins in mammalian cells. *J. Cell Biol.* 196, 801-810. 10.1083/jcb.201112098 22412018 PMC3308701

[JCS261119C73] Saeki, K., Miura, Y., Aki, D., Kurosaki, T. and Yoshimura, A. (2003). The B cell-specific major raft protein, Raftlin, is necessary for the integrity of lipid raft and BCR signal transduction. *EMBO J.* 22, 3015-3026. 10.1093/emboj/cdg293 12805216 PMC162145

[JCS261119C74] Saito, K., Scharenberg, A. M. and Kinet, J. P. (2001). Interaction between the Btk PH domain and phosphatidylinositol-3,4,5-trisphosphate directly regulates Btk. *J. Biol. Chem.* 276, 16201-16206. 10.1074/jbc.M100873200 11279148

[JCS261119C75] Samavarchi-Tehrani, P., Samson, R. and Gingras, A. C. (2020). Proximity dependent biotinylation: key enzymes and adaptation to proteomics approaches. *Mol. Cell. Proteomics* 19, 757-773. 10.1074/mcp.R120.001941 32127388 PMC7196579

[JCS261119C76] Sarapulov, A. V., Petrov, P., Hernández-Pérez, S., Šuštar, V., Kuokkanen, E., Cords, L., Samuel, R. V. M., Vainio, M. and Mattila, P. K. (2020). Missing-in-metastasis/metastasis suppressor 1 regulates B cell receptor signaling, B cell metabolic potential, and T cell-independent immune responses. *Front. Immunol.* 11, 599. 10.3389/fimmu.2020.00599 32373113 PMC7176992

[JCS261119C77] Satpathy, S., Wagner, S. A., Beli, P., Gupta, R., Kristiansen, T. A., Malinova, D., Francavilla, C., Tolar, P., Hostager, B. S. and Choudhary, C. (2015). Systems–wide analysis of BCR signalosomes and downstream phosphorylation and ubiquitylation. *Mol. Syst. Biol.* 11, 810. 10.15252/msb.20145880 26038114 PMC4501846

[JCS261119C78] Schindelin, J., Arganda-Carreras, I., Frise, E., Kaynig, V., Longair, M., Pietzsch, T., Preibisch, S., Rueden, C., Schmid, B., Cardona, A. et al. (2012). Fiji: an open-source platform for biological-image analysis. *Nat. Methods* 9, 676-682. 10.1038/nmeth.2019 22743772 PMC3855844

[JCS261119C79] Sezgin, E., Levental, I., Mayor, S. and Eggeling, C. (2017). The mystery of membrane organization: composition, regulation and roles of lipid rafts. *Nat. Rev. Mol. Cell Biol.* 18, 361-374. 10.1038/nrm.2017.16 28356571 PMC5500228

[JCS261119C80] Shevchenko, A., Tomas, H., Havliš, J., Olsen, J. V. and Mann, M. (2007). In-gel digestion for mass spectrometric characterization of proteins and proteomes. *Nat. Protoc.* 1, 2856-2860. 10.1038/nprot.2006.468 17406544

[JCS261119C81] Sohn, H. W., Tolar, P., Jin, T. and Pierce, S. K. (2006). Fluorescence resonance energy transfer in living cells reveals dynamic membrane changes in the initiation of B cell signaling. *Proc. Natl. Acad. Sci. USA* 103, 8143-8148. 10.1073/pnas.0509858103 16690746 PMC1472443

[JCS261119C82] Sohn, H. W., Tolar, P. and Pierce, S. K. (2008). Membrane heterogeneities in the formation of B cell receptor–Lyn kinase microclusters and the immune synapse. *J. Cell Biol.* 182, 367-379. 10.1083/jcb.200802007 18644892 PMC2483512

[JCS261119C83] Stone, M. B., Shelby, S. A., Nńñez, M. F., Wisser, K. and Veatch, S. L. (2017). Protein sorting by lipid phase-like domains supports emergent signaling function in b lymphocyte plasma membranes. *ELife* 6, e19891. 10.7554/eLife.19891 28145867 PMC5373823

[JCS261119C84] Susa, K. J., Bradshaw, G. A., Eisert, R. J., Schilling, C. M., Kalocsay, M., Blacklow, S. C. and Kruse, A. C. (2023). A spatiotemporal map of co-receptor signaling networks underlying B cell activation. *bioRxiv* 2023.03.17.533227. 10.1101/2023.03.17.533227 PMC11256977 38850533

[JCS261119C85] Šuštar, V., Vainio, M. and Mattila, P. K. (2018). Visualization and quantitative analysis of the actin cytoskeleton upon B cell activation. *Methods Mol. Biol.* 1707, 243-257. 10.1007/978-1-4939-7474-0_18 29388113

[JCS261119C86] Varshney, P., Yadav, V. and Saini, N. (2016). Lipid rafts in immune signalling: current progress and future perspective. *Immunology* 149, 13-24. 10.1111/imm.12617 27153983 PMC4981613

[JCS261119C87] Vilen, B. J., Nakamura, T. and Cambier, J. C. (1999). Antigen-stimulated dissociation of BCR mlg from Ig-α/Ig-β: implications for receptor desensitization. *Immunity* 10, 239-248. 10.1016/S1074-7613(00)80024-2 10072076 PMC3931429

[JCS261119C89] Wienands, J., Larbolette, O. and Reth, M. (1996). Evidence for a preformed transducer complex organized by the B cell antigen receptor. *Proc. Natl. Acad. Sci. USA* 93, 7865-7870. 10.1073/pnas.93.15.7865 8755568 PMC38840

[JCS261119C90] Willforss, J., Chawade, A. and Levander, F. (2019). NormalyzerDE: online tool for improved normalization of omics expression data and high-sensitivity differential expression analysis. *J. Proteome Res.* 18, 732-740. 10.1021/acs.jproteome.8b00523 30277078

[JCS261119C91] Williams, D., Hicks, S. W., Machamer, C. E. and Pessin, J. E. (2006). Golgin-160 is required for the Golgi membrane sorting of the insulin-responsive glucose transporter GLUT4 in adipocytes. *Mol. Biol. Cell* 17, 5346-5355. 10.1091/mbc.e06-05-0386 17050738 PMC1679696

[JCS261119C92] Wong, L. E., Bhatt, A., Erdmann, P. S., Hou, Z., Maier, J., Pirkuliyeva, S., Engelke, M., Becker, S., Plitzko, J. and Griesinger, C. (2020). Tripartite phase separation of two signal effectors with vesicles priming B cell responsiveness. *Nat. Commun.* 11, 848. 10.1038/s41467-020-14544-1 32051419 PMC7016142

[JCS261119C93] Xu, Y., Harder, K. W., Huntington, N. D., Hibbs, M. L. and Tarlinton, D. M. (2005). Lyn tyrosine kinase: accentuating the positive and the negative. *Immunity* 22, 9-18. 15664155 10.1016/j.immuni.2004.12.004

[JCS261119C94] Yadav, S., Puthenveedu, M. A. and Linstedt, A. D. (2012). Golgin160 recruits the dynein motor to position the golgi apparatus. *Dev. Cell* 23, 153-165. 10.1016/j.devcel.2012.05.023 22814606 PMC3417773

[JCS261119C95] Yang, J. and Reth, M. (2010). The dissociation activation model of B cell antigen receptor triggering. *FEBS Lett.* 584, 4872-4877. 10.1016/j.febslet.2010.09.045 20920502

[JCS261119C96] Yang, W., Di Vizio, D., Kirchner, M., Steen, H. and Freeman, M. R. (2010). Proteome scale characterization of human S-acylated proteins in lipid raft-enriched and non-raft membranes. *Mol. Cell. Proteomics* 9, 54-70. 10.1074/mcp.M800448-MCP200 19801377 PMC2808267

[JCS261119C97] Yasuda, K., Kosugi, A., Hayashi, F., Saitoh, S., Nagafuku, M., Mori, Y., Ogata, M. and Hamaoka, T. (2000). Serine 6 of Lck tyrosine kinase: a critical site for Lck myristoylation, membrane localization, and function in T Lymphocytes. *J. Immunol* 165, 3226-3231. 10.4049/jimmunol.165.6.3226 10975838

[JCS261119C98] Yu, G., Wang, L.-G., Han, Y. and He, Q.-Y. (2012). clusterProfiler: an R package for comparing biological themes among gene clusters. *Omics J. Integr. Biol.* 16, 284-287. 10.1089/omi.2011.0118 PMC3339379 22455463

