# Supplementary material for: B cell receptor-induced protein dynamics and the emerging role of SUMOylation revealed by proximity proteomics
Source: J Cell Sci. 2023 Aug 8;136(15):jcs261119. doi: 10.1242/jcs.261119 (PMC10445728; doi:10.1242/jcs.261119)
Supplement: Supplementary information [file joces-136-261119-s1.pdf]

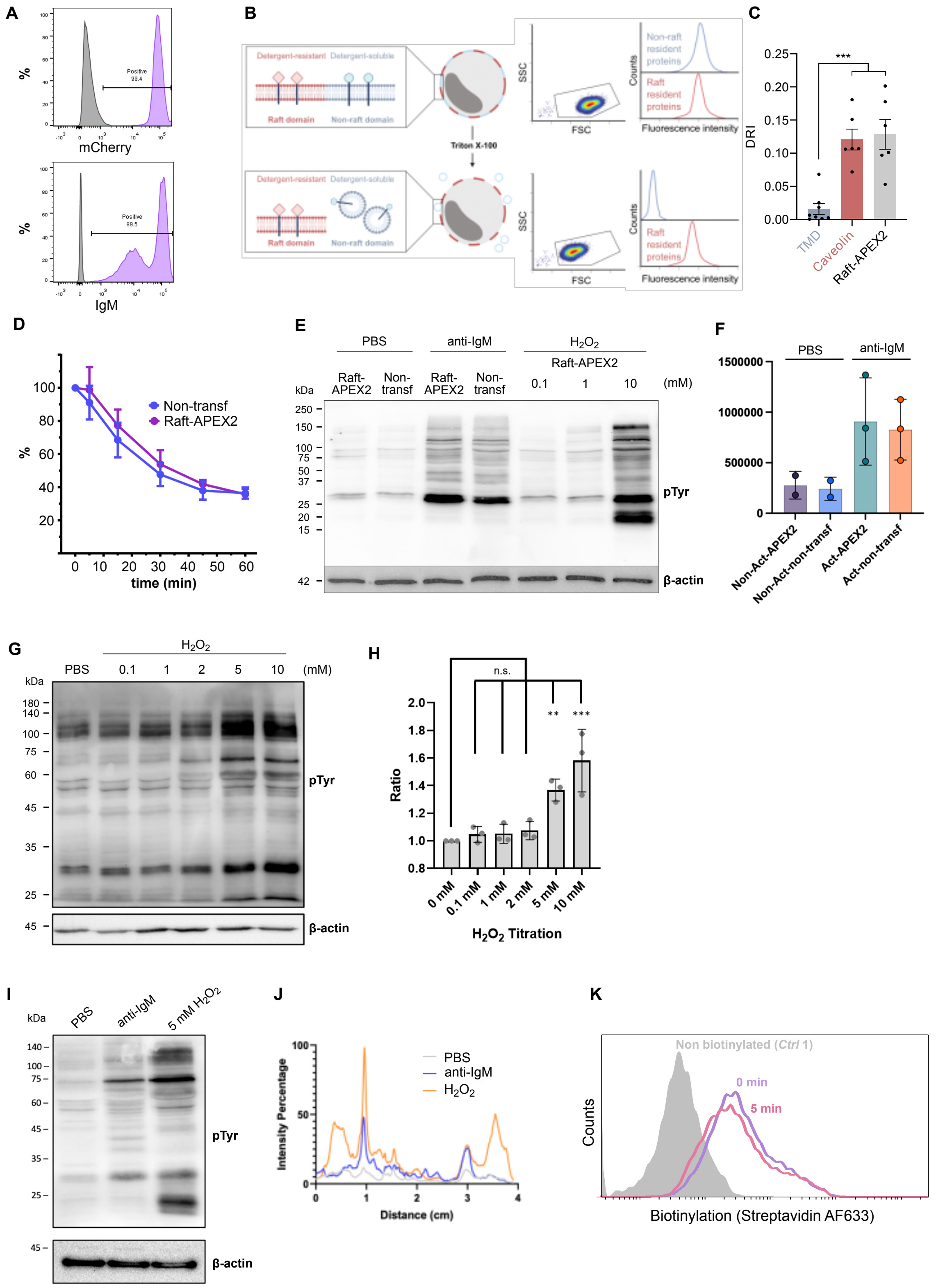

Fig. S1. Raft-APEX2 construct locates in the detergent-resistant membrane domains. Related to Fig. 1. **A)** Raft-APEX2 expressing A20 D1.3 cells used in the proteomic experiments were analysed for the expression levels of Raft-APEX2, by mCherry fluorescence (upper blot), and IgM BCR by fluorescently labelling the IgM (lower blot) using flow cytometry (n=3). **B)** A schematic representation of flow cytometry assay to detect lipid raft association of membrane proteins by analysis of detergent resistance. Upon treatment with TritonX- 100 detergent, the proteins within lipid rafts are retained, whereas non-raft domains are dissolved. **C)** A20 D1.3 B cells were transfected with raft-APEX2, lipid raft marker (caveolin-1-RFP) or non-raft marker (TMD-GFP). The fluorescence of the markers was measured before, and after subjecting the samples to 0.1% Triton X-100 and

Journal of Cell Science • Supplementary information

the detergent resistance index was calculated as described in Materials and Methods (n = 6-8 independent experiments; unpaired t-test, *** < 0.001). **D)** BCR internalization assay by flow cytometry. A20 D1.3 cells transfected or not with Raft-APEX2 were stimulated with 10 µg/ml biotinylated anti-IgM F(ab')$_2$ fragments for 0, 5, 15, 30, 45, or 60 min, and the IgM remaining on the cell surface was detected with AF633-labelled streptavidin. Paired t-test, mean +/-SEM of three experiments. **E)** The original immunoblot that is shown rearranged in the main Figure 1 D and E. Raft-APEX2 expressing A20 D1.3 B cells and the parental A20 D1.3 (non-transfected) cells were treated with 0, 0.1, 1 and 10 mM H_2O_2, or 10 µg/ml anti-IgM F(ab')$_2$ fragments. Cells were lysed and subjected to Western blotting. The membranes were probed with HRP-anti phospho-Tyrosine antibodies and anti-β-actin as a loading control. **F)** Quantification of the pTyr-response of Raft-APEX2 expressing, or not, A20 D1.3 cells to anti-IgM F(ab')$_2$, from (E). T-test, mean +/-SEM of three experiments. **G)** An immunoblot like in (C), using Raft-APEX2 expressing A20 D1.3 B cells treated with 0, 0.1, 1, 2, 5 and 10 mM H_2O_2 for 1 min. **H)** Quantification of (G). One-way ANOVA, mean +/-SEM of three experiments. ** p < 0.01, *** p < 0.001. **I)** A pTyr-immunoblot of A20 D1.3 cells treated, or not, with 5 mM H_2O_2 for 1 min or 10 µg/ml anti-IgM F(ab')$_2$ for 10 min. **J)** Line plot comparison of the H_2O_2 and anti-IgM F(ab')$_2$ activated lanes in (I) (n=3, representative plot shown). **K)** Raft-APEX2 A20 D1.3 B cells were supplemented with biotin-phenol, activated (red line) or not with (violet line) F(ab')$_2$ fragments of anti-IgM antibodies for 5 min, and the biotinylation was triggered or not (grey line) by adding 1 mM H_2O_2 for 1 min. Cells were fixed with 4% PFA, permeabilised, stained with AF633-labelled streptavidin and analyzed with flow cytometry. n=3, representative plot shown.

Journal of Cell Science • Supplementary information

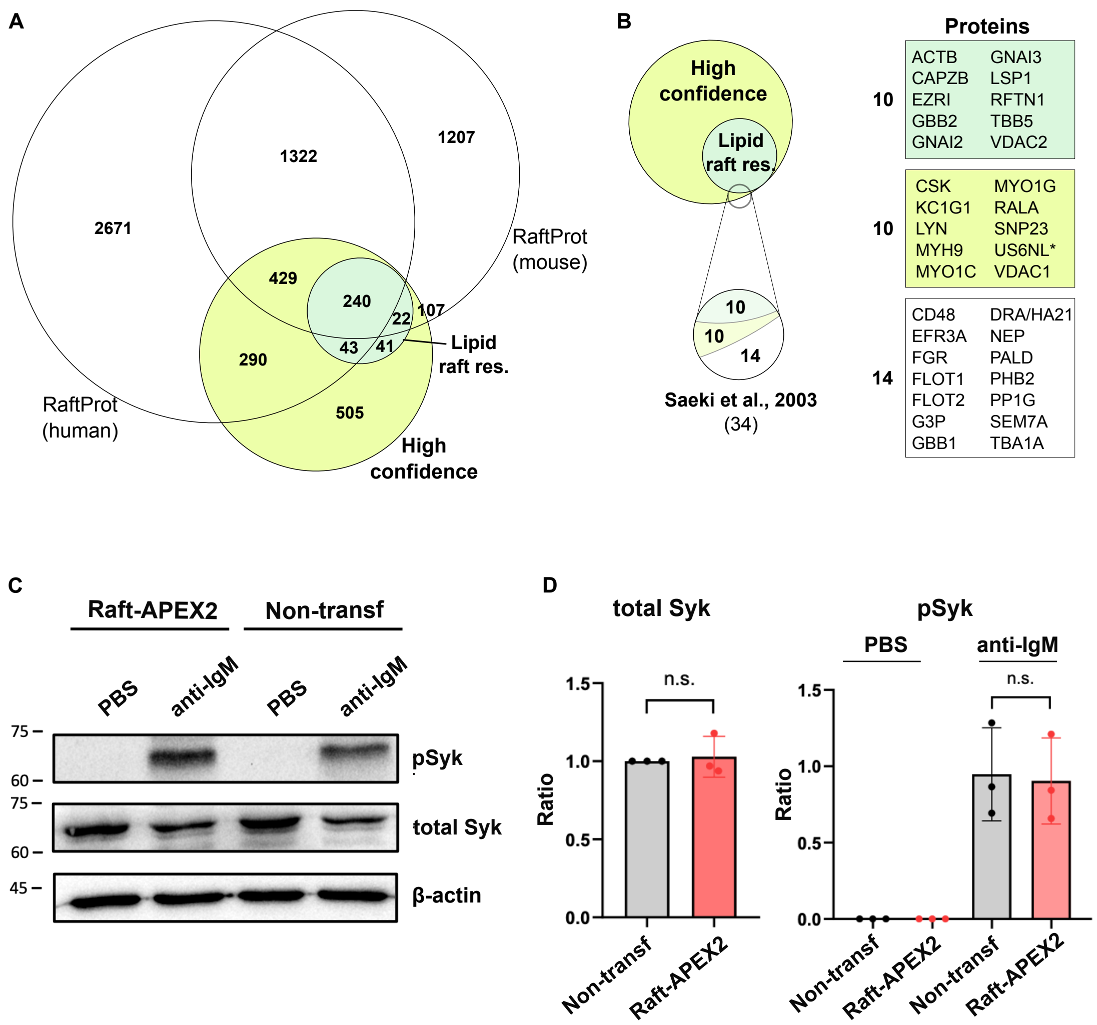

Fig. S2. Lipid raft-resident proteins in B cells. Related to Fig. 3. **A)** A Venn diagram showing the intersection between the data obtained in this study (Fig. 2C) to both mouse and human raft proteins in the RaftProt database. **B)** A Venn diagram showing the intersection between the data obtained in this study and the lipid raft proteins identified in Raji B cells by Saeki et al. (Saeki et al., 2003). **C)** A20 D1.3 cells transfected or not with Raft-APEX2 were stimulated, or not, with 10 µg/ml anti-IgM F(ab')$_2$ fragments for 10 min and immunoblotted for total Syk, phosphorylated Syk and β-actin for loading control (n=3). **D)** Quantification of (C). Paired t-test, data is shown as mean ± SEM, * $p < 0.05$.

Journal of Cell Science • Supplementary information

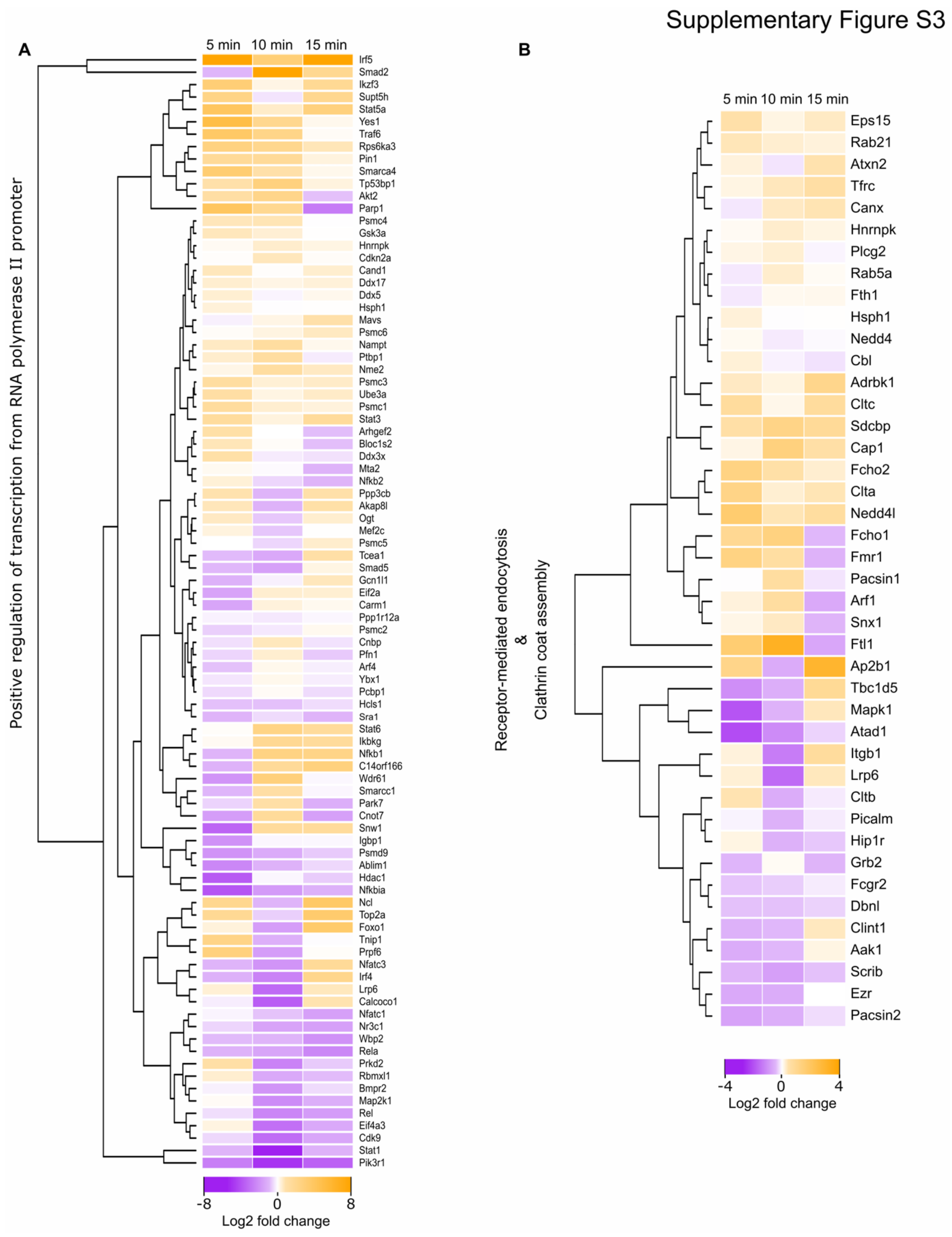

Fig. S3. Dynamics of proteins linked to transcription and endocytosis. A) The proteomic dataset (described in Fig 2C) was analyzed to generate a heatmap of the intensity changes of proteins within the GO term “positive regulation of transcription from RNA polymerase II promoter”. **B)** Heatmap of the intensity changes of proteins within the GO terms “receptor-mediated endocytosis” and “clathrin coat assembly”.

Supplementary Figure S4

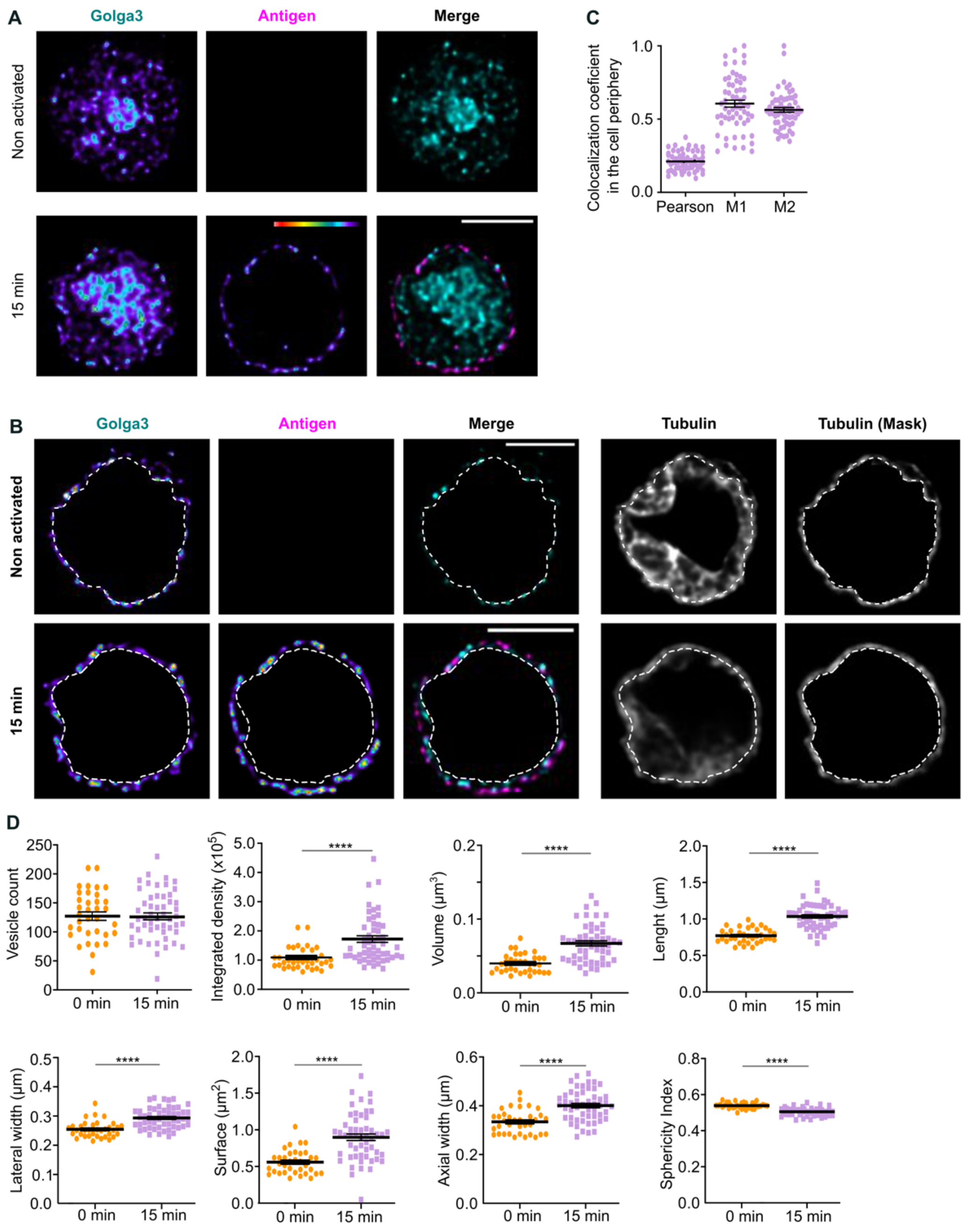

Fig. S4. Translocation of Golga3 to the cell periphery upon BCR activation and colocalization with antigen. A) Human Raji D1.3 B cells were let to adhere on CellTak-coated microscopy coverslips, activated (lower panel) or not (upper panel) with 10 µg/ml Alexa Fluor®-labelled F(ab')$_2$ fragments of anti-IgM antibodies (antigen, pseudocolour in single-channel image and magenta in merge) for 15 min, fixed and permeabilised and subjected to immunofluorescence analysis with anti-Golga3 (cyan) antibodies. Cells were imaged with spinning disc confocal microscopy. **B)** A 3D mask (dotted line) was generated from the cytosolic tubulin signal, and the inside region of the mask was cleared from signal in all channels. Single confocal planes from deconvoluted images of representative cells (same as in A), showing the filtering, are shown. Scale bar 5 µm. The resulting peripheral Golga3 signal was processed for colocalization analysis with antigen (AF647-anti-IgM F(ab')$_2$) (C) as well as 3D particle analysis (D) were carried out with Huygens. **C-D)** The colocalization of antigen and Golga3 (C)

Journal of Cell Science • Supplementary information

as well as the Golga3 vesicles (D) were analyzed from images deconvoluted at the antigen and Golga3 channels, exclusively from the cell periphery, with the filtering illustrated in B. C) The levels of colocalization were measured with Pearson's correlation coefficiency (0.21) and Manders' overlap coefficiency (M1: 0.61, M2: 0.56). D) The intensity, volume and length of the peripheral Golga3 vesicles are shown, as well as the vesicle count per cell, lateral and axial width (µm), sphericity index (1=full sphere) and surface area (μm^2). Data is shown as mean ± SEM from ³ 35 cells from three independent experiments, analysed by t-test. ****: $p < 0.0001$

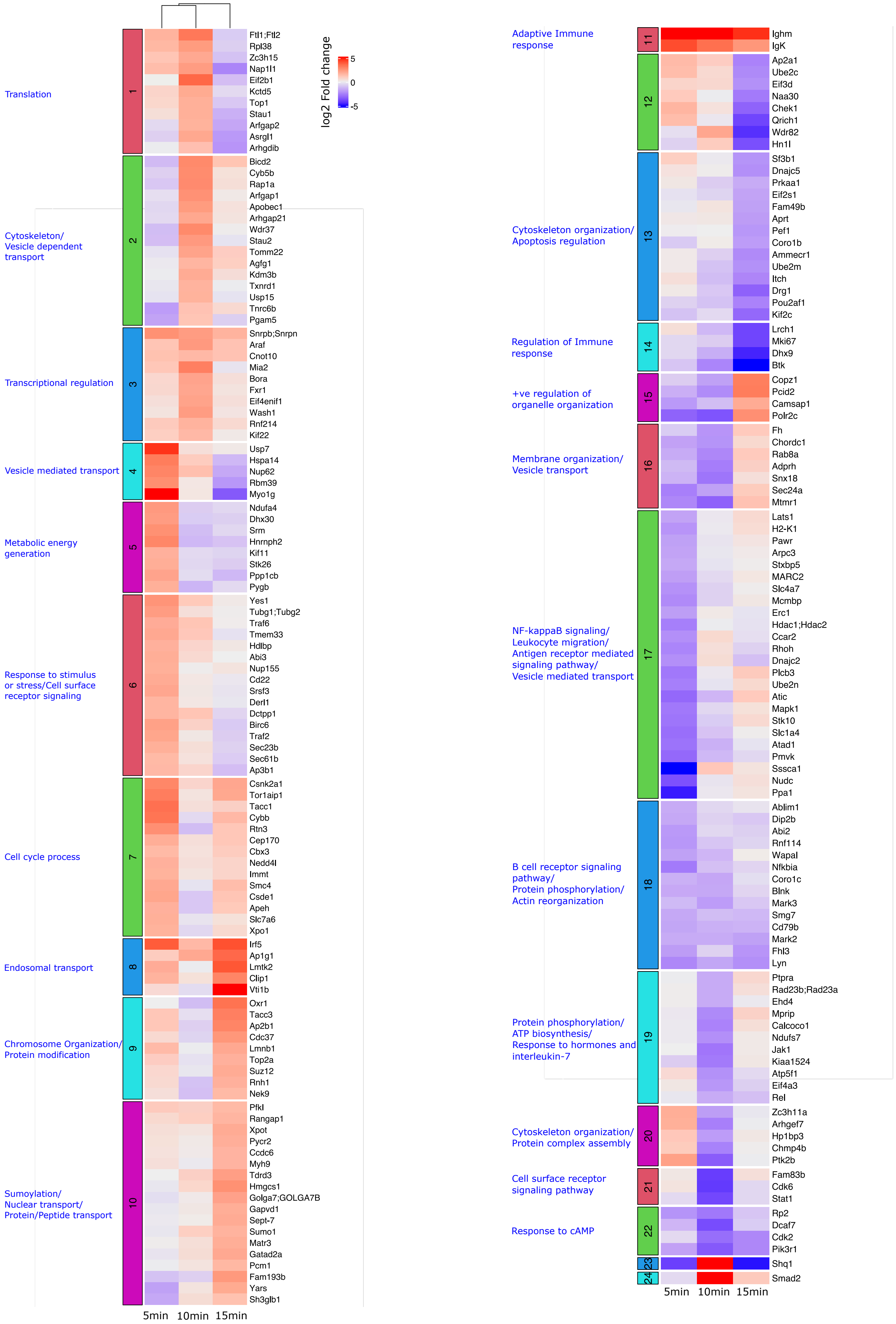

Journal of Cell Science • Supplementary information

Fig. S5. Protein clustering analysis. Heat map showing k-means clustering of fold-changes of combined differentially expressed proteins in any of the time points, in our dataset (Fig 2C). The purple bands indicate low expression levels while the red bands indicate high expression levels. The optimal number of clusters, k, was determined to be 24 prior to performing k-means clustering. The associated gene ontology (GO), biological processes (BP) of the proteins in each cluster were then determined using DAVID.

Supplementary Figure S6

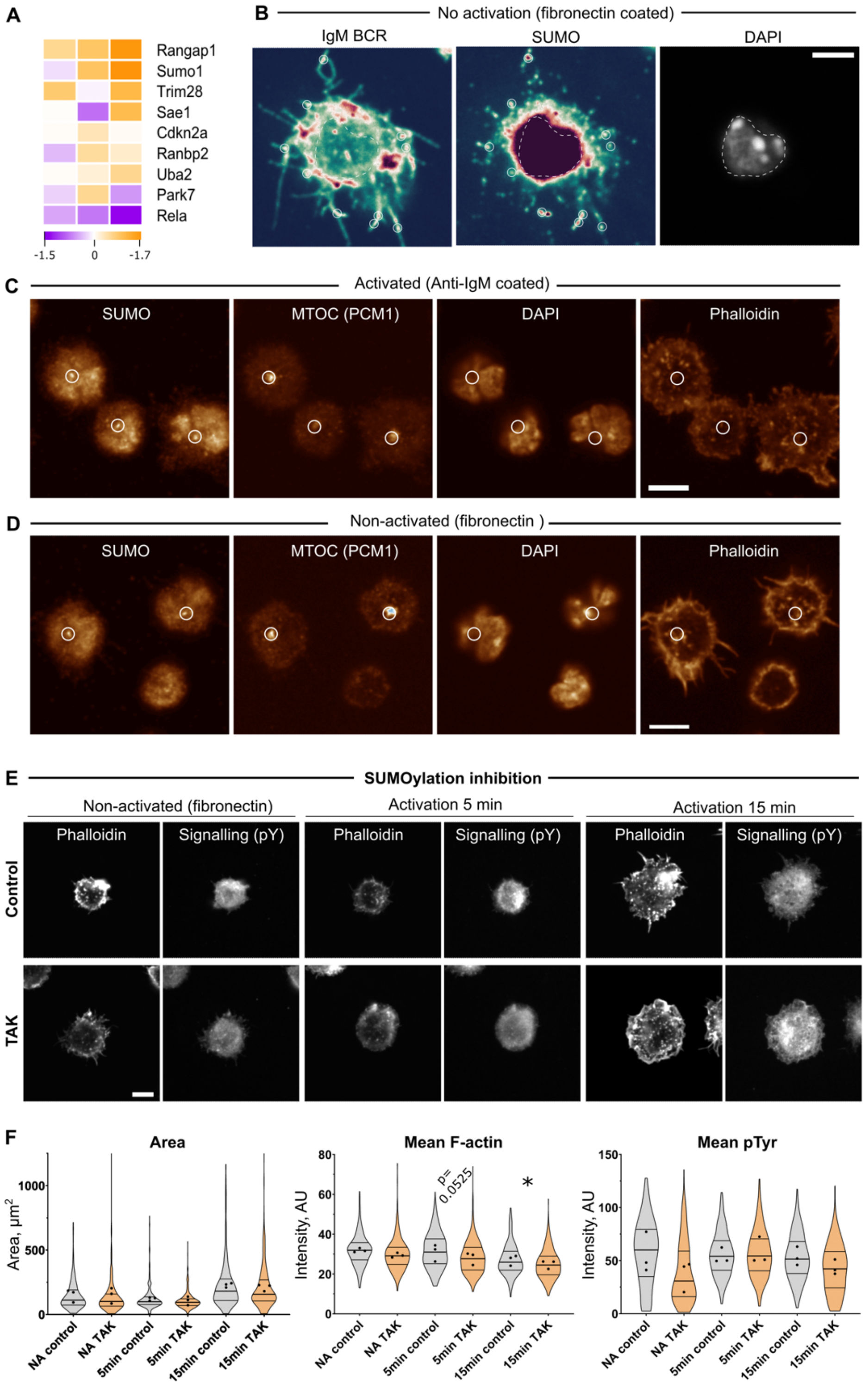

Fig. S6. The vicinity of the MTOC is enriched in SUMOylation. Related to Fig. 6 and 7. **A)** Heatmap showing the expression level of proteins associated with SUMOylation in our dataset (Fig. 2C) based on gene ontology (biological process). The purple bands indicate low expression levels while the yellow/orange bands indicate high expression levels. **B)** Representative spinning dick confocal microscopy images of non-activated A20 D1.3 cells illustrating the colocalization of the BCR and SUMO (indicated by white circles in the two first panels) and the nucleus (dotted outline in the three panels). Cells were plated on fibronectin, fixed and stained using Donkey anti-mouse IgM F(ab´)$_2$, anti-Sumo1 antibody and DAPI. **C-D)** Representative images of activated (**C**) and non-activated (**D**) A20 D1.3 cells showing the colocalization of SUMO and MTOC (indicated by the white circle). Cells were plated on fibronectin or anti-IgM F(ab´)$_2$ coated surface, fixed and stained with anti-Sumo1 antibody, Anti-PCM1 antibody, DAPI and phalloidin for F-actin. The experiments in (B-D) were performed

Journal of Cell Science • Supplementary information

3 times. ≈10-30 cells/condition/experiment were imaged, and representative examples shown. **E-F)** A20 D1.3 cells were treated with 25 μM TAK-981, to inhibit SUMOylation, or DMSO as control. Cells were plated on fibronectin for non-activatory conditions or on anti-IgM F(ab´)$_2$ for activation for 5 or 15 min, fixed and stained with anti-pTyr antibody and phalloidin for F-actin (E). **F)** Quantification of TAK-981 inhibited and control cells in (D). Area of the cell spreading was analyzed by thresholding the phalloidin channel at the cell-surface interface. F-actin and pTyr mean intensity values were measured in the thresholded area. All the samples were imaged with spinning disc confocal microscopy, individual slices are shown. Data is shown as violin plots and mean ± SEM of all cells overlaid with individual mean values of three independent experiments (≈70-100 cells per experiment). Paired t-test on mean values of each experiment (n=3) was performed. *: $p<0.05$. Scale bars = 5μm.

Journal of Cell Science • Supplementary information

Supplementary Fig S7, data integrity, 1/16

Figure 7A

primary B cells
Sumo1

1.	0 min	soluble	DMSO	plain RPMI
2.	0 min	soluble	TAK981 (25 uM)	plain RPMI
3.	15 min	soluble	DMSO	plain RPMI
4.	15 min	soluble	TAK981 (25 uM)	plain RPMI
5.	0 min	surface	DMSO	plain RPMI
6.	0 min	surface	TAK981 (25 uM)	plain RPMI
7.	15 min	surface	DMSO	plain RPMI
8.	15 min	surface	TAK981 (25 uM)	plain RPMI

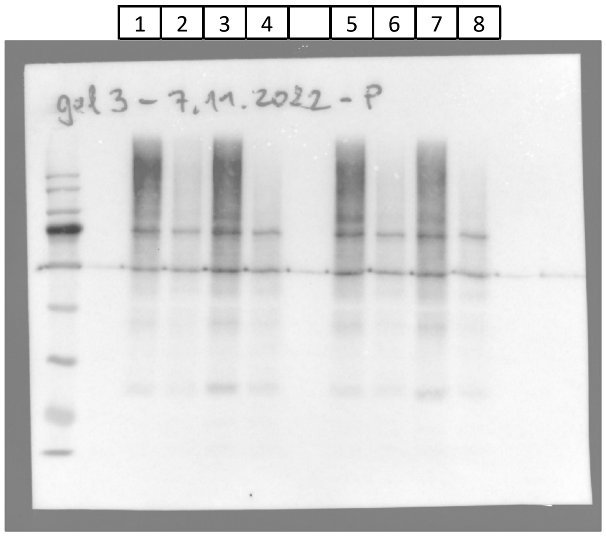

Sumo1

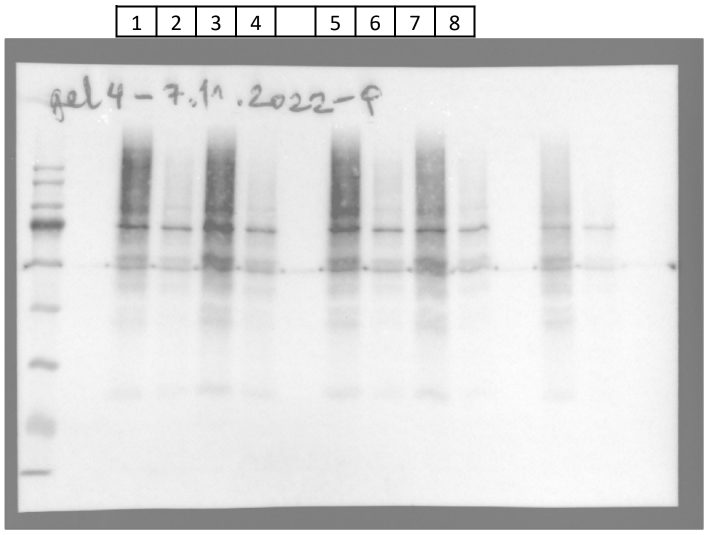

Sumo1

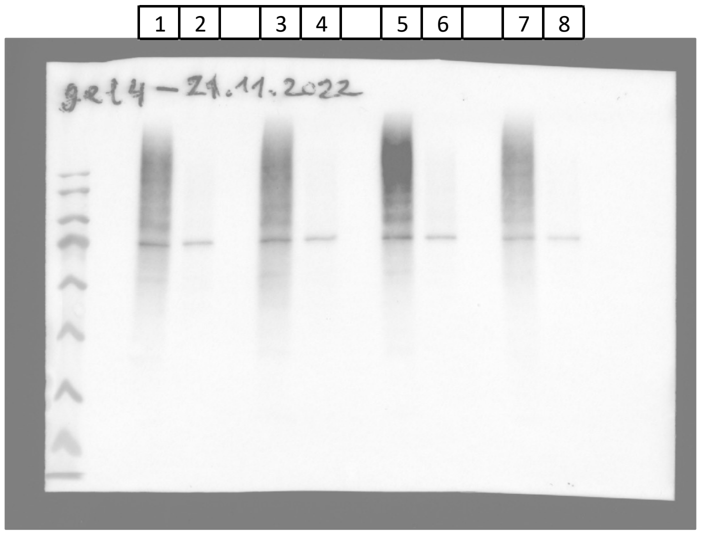

Sumo1

Journal of Cell Science • Supplementary information

Supplementary Fig S7, data integrity, 2/16

Figure 7A

A20 D1.3 B cell line
Sumo1

1.	0 min	soluble	DMSO	plain RPMI
2.	0 min	soluble	TAK981 (25 uM)	plain RPMI
3.	15 min	soluble	DMSO	plain RPMI
4.	15 min	soluble	TAK981 (25 uM)	plain RPMI
5.	0 min	surface	DMSO	plain RPMI
6.	0 min	surface	TAK981 (25 uM)	plain RPMI
7.	15 min	surface	DMSO	plain RPMI
8.	15 min	surface	TAK981 (25 uM)	plain RPMI

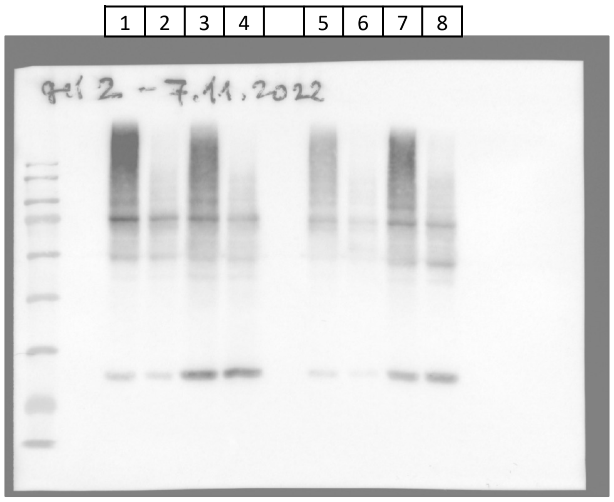

Sumo1

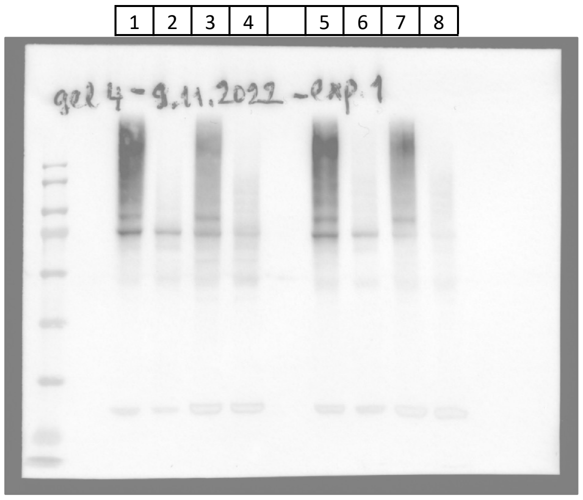

Sumo1

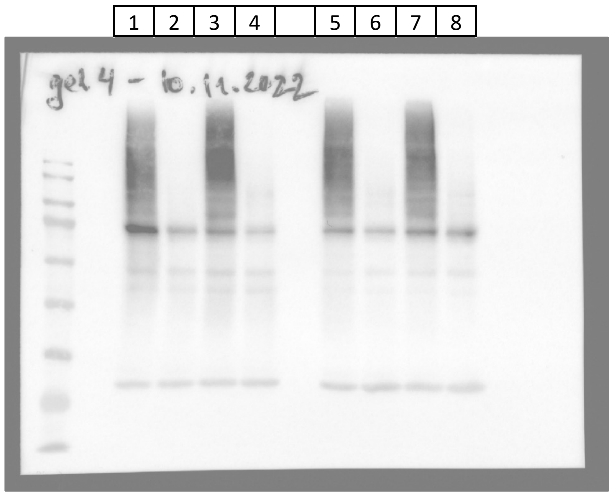

Sumo1

Journal of Cell Science • Supplementary information

<u>*Supplementary Fig S7, data integrity, 3/16*</u>

Figure 7B, C

primary B cells
pAkt S473; Akt

1.	0 min	soluble	DMSO	plain RPMI
2.	0 min	soluble	TAK981 (25 uM)	plain RPMI
3.	15 min	soluble	DMSO	plain RPMI
4.	15 min	soluble	TAK981 (25 uM)	plain RPMI
5.	0 min	surface	DMSO	plain RPMI
6.	0 min	surface	TAK981 (25 uM)	plain RPMI
7.	15 min	surface	DMSO	plain RPMI
8.	15 min	surface	TAK981 (25 uM)	plain RPMI

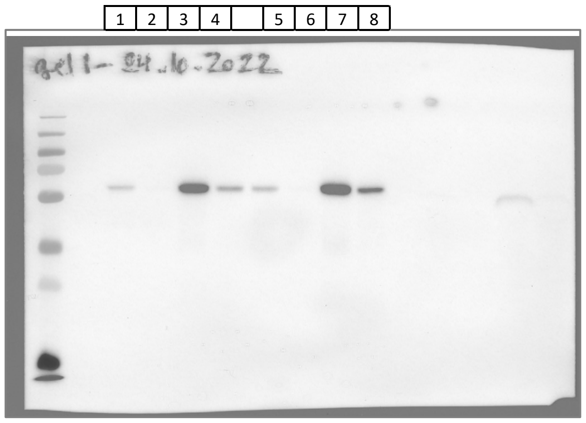

pAkt S473

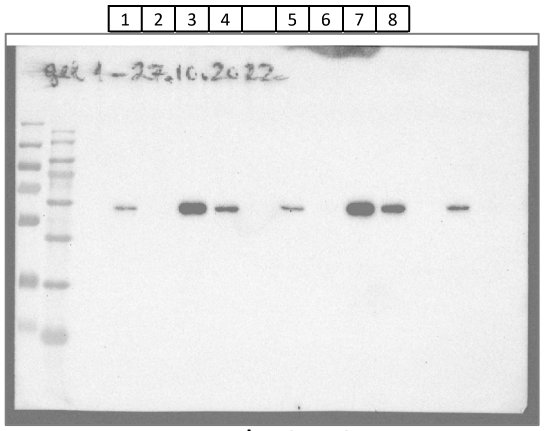

pAkt S473

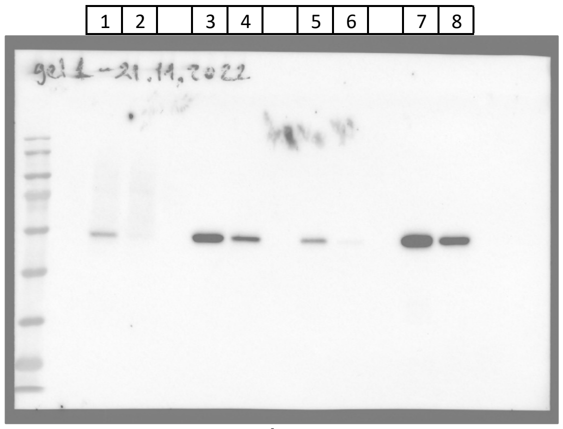

pAkt S473

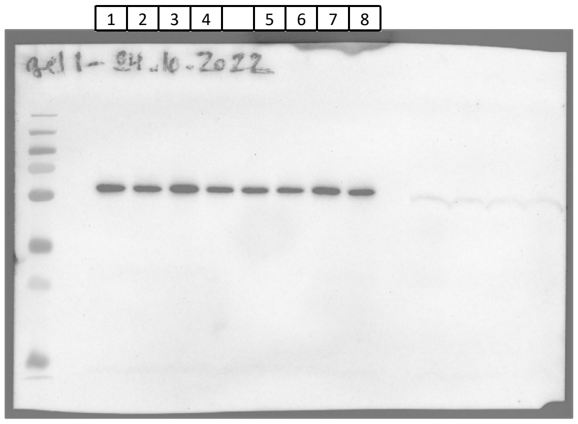

total Akt

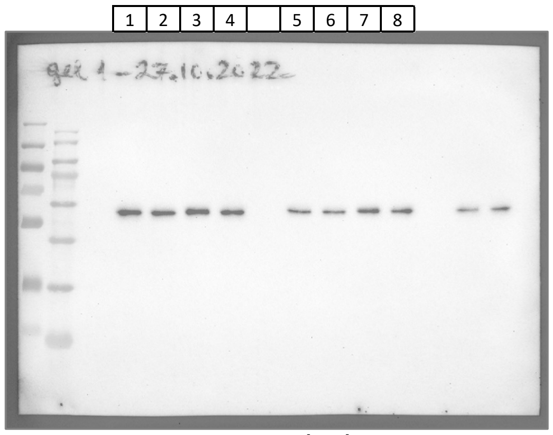

total Akt

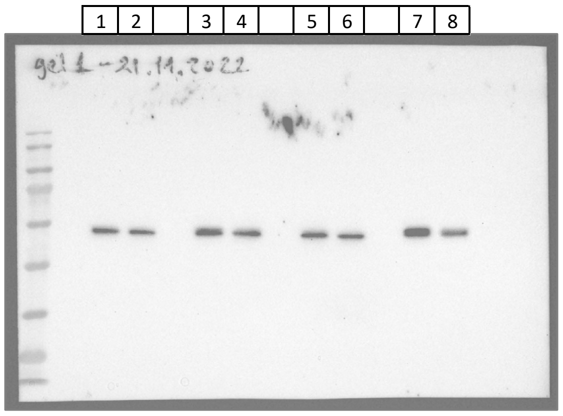

total Akt

Journal of Cell Science • Supplementary information

Supplementary Fig S7, data integrity, 4/16

Figure 7B, C

primary B cells

pMAPK1/2; MAPK1/2

1.	0 min	soluble	DMSO	plain RPMI
2.	0 min	soluble	TAK981 (25 uM)	plain RPMI
3.	15 min	soluble	DMSO	plain RPMI
4.	15 min	soluble	TAK981 (25 uM)	plain RPMI
5.	0 min	surface	DMSO	plain RPMI
6.	0 min	surface	TAK981 (25 uM)	plain RPMI
7.	15 min	surface	DMSO	plain RPMI
8.	15 min	surface	TAK981 (25 uM)	plain RPMI

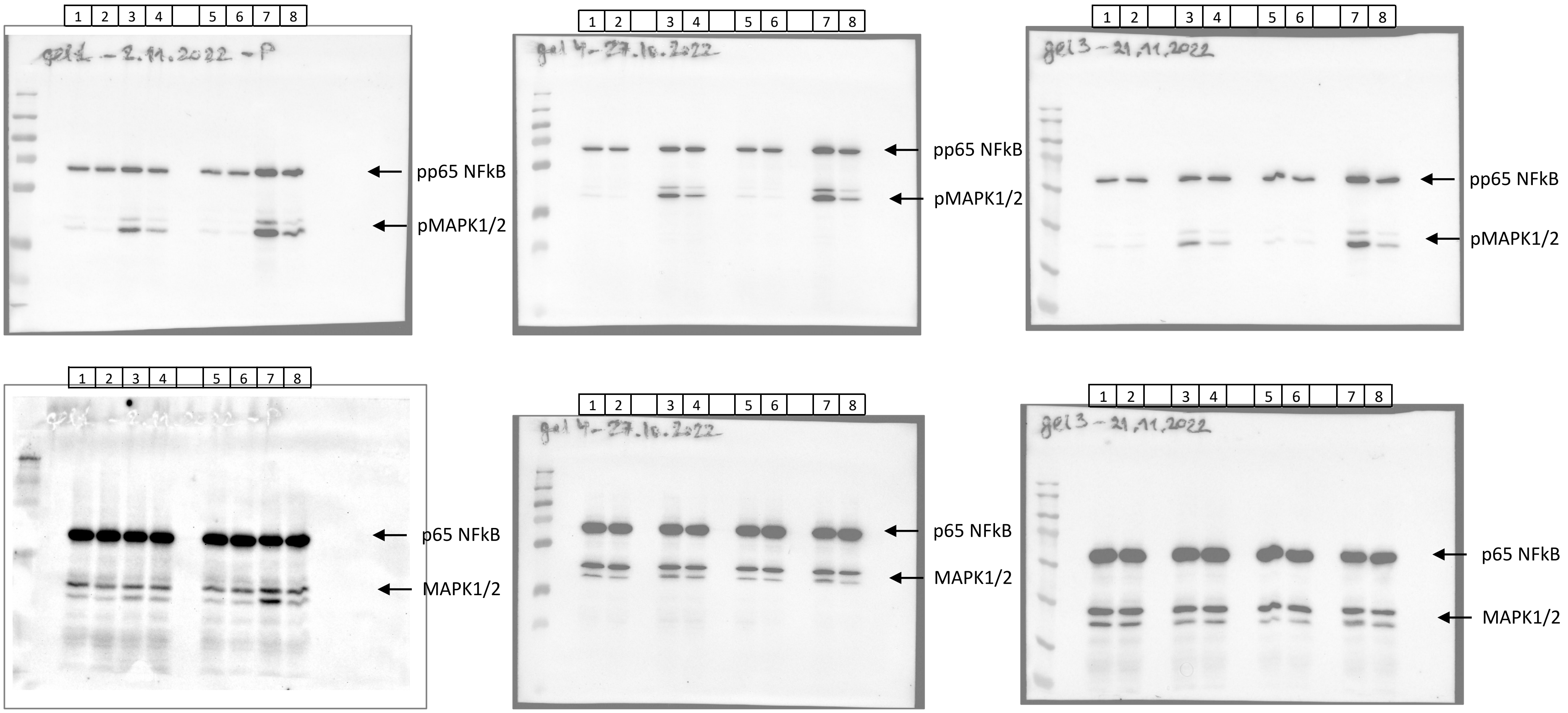

Journal of Cell Science • Supplementary information

Supplementary Fig S7, data integrity, 5/16

Figure 7B, C

primary B cells

pSyk; Syk

1.	0 min	soluble	DMSO	plain RPMI
2.	0 min	soluble	TAK981 (25 uM)	plain RPMI
3.	15 min	soluble	DMSO	plain RPMI
4.	15 min	soluble	TAK981 (25 uM)	plain RPMI
5.	0 min	surface	DMSO	plain RPMI
6.	0 min	surface	TAK981 (25 uM)	plain RPMI
7.	15 min	surface	DMSO	plain RPMI
8.	15 min	surface	TAK981 (25 uM)	plain RPMI

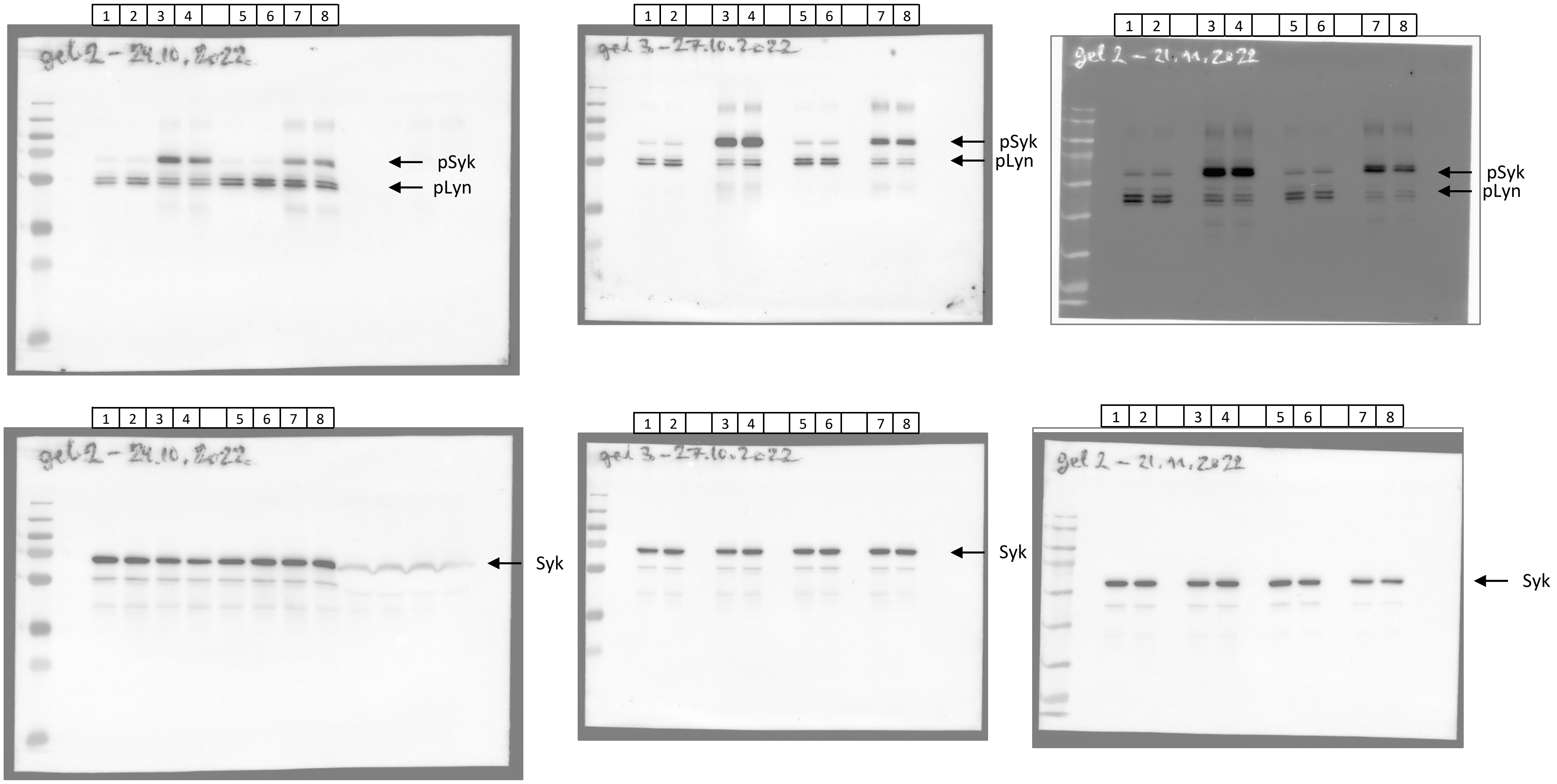

Journal of Cell Science • Supplementary information

Supplementary Fig S7, data integrity, 6/16

Figure 7B, C

A20 D1.3 B cell line

pAkt S473; Akt

1.	0 min	soluble	DMSO	plain RPMI
2.	0 min	soluble	TAK981 (25 uM)	plain RPMI
3.	15 min	soluble	DMSO	plain RPMI
4.	15 min	soluble	TAK981 (25 uM)	plain RPMI
5.	0 min	surface	DMSO	plain RPMI
6.	0 min	surface	TAK981 (25 uM)	plain RPMI
7.	15 min	surface	DMSO	plain RPMI
8.	15 min	surface	TAK981 (25 uM)	plain RPMI

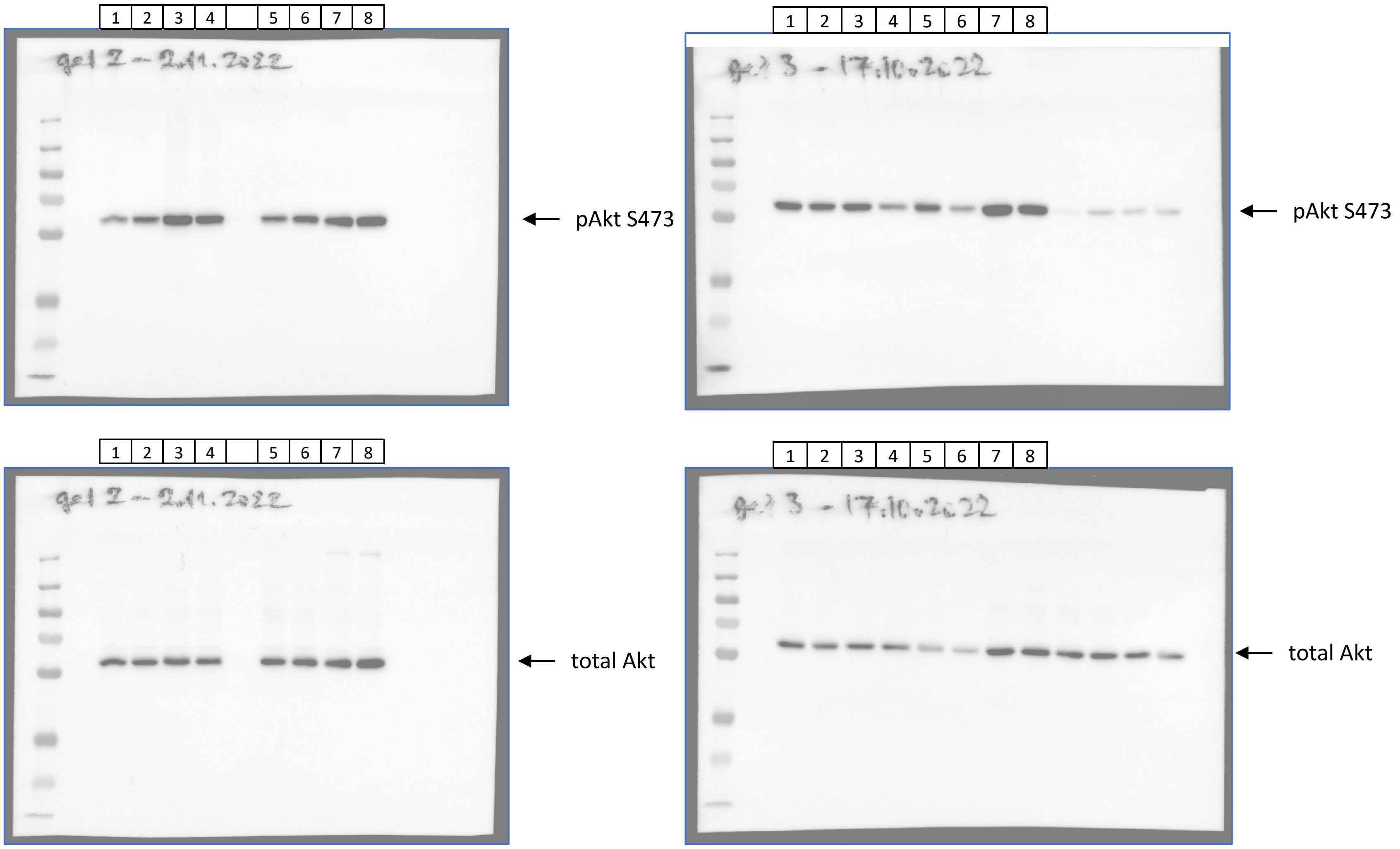

Journal of Cell Science • Supplementary information

Supplementary Fig S7, data integrity, 7/16

Figure 7B, C

A20 D1.3 B cell line

pAkt S473; Akt

1.	0 min	soluble	DMSO	plain RPMI
2.	0 min	soluble	TAK981 (25 uM)	plain RPMI
3.	15 min	soluble	DMSO	plain RPMI
4.	15 min	soluble	TAK981 (25 uM)	plain RPMI
5.	0 min	surface	DMSO	plain RPMI
6.	0 min	surface	TAK981 (25 uM)	plain RPMI
7.	15 min	surface	DMSO	plain RPMI
8.	15 min	surface	TAK981 (25 uM)	plain RPMI

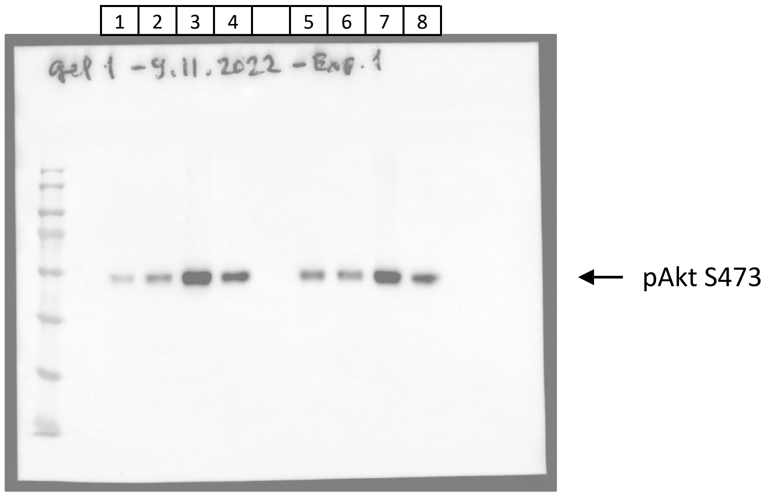

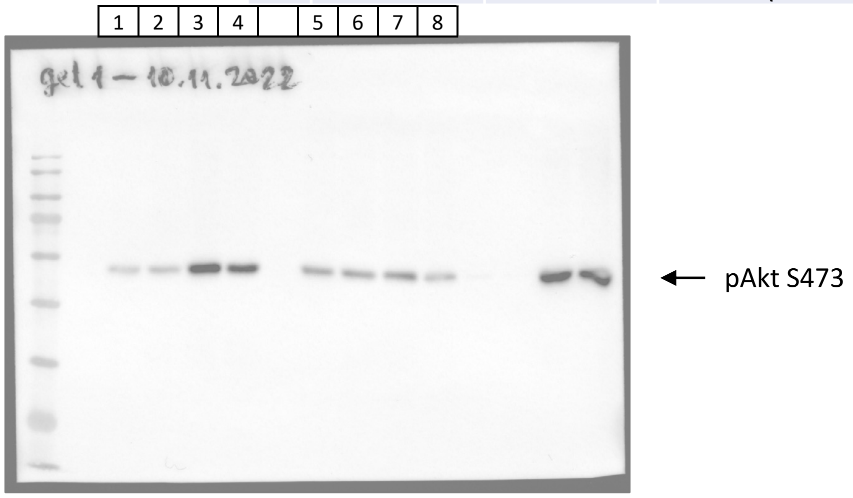

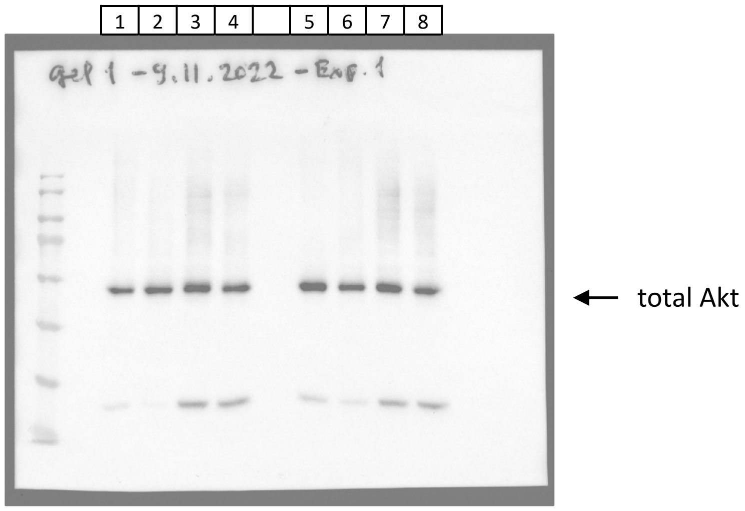

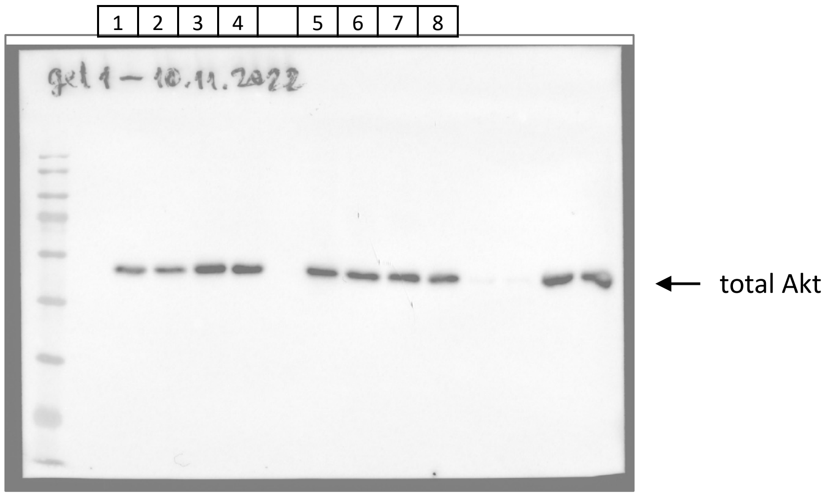

Journal of Cell Science • Supplementary information

Supplementary Fig S7, data integrity, 8/16

Figure 7B, C

A20 D1.3 B cell line

pMAPK1/2; MAPK1/2

1.	0 min	soluble	DMSO	plain RPMI
2.	0 min	soluble	TAK981 (25 uM)	plain RPMI
3.	15 min	soluble	DMSO	plain RPMI
4.	15 min	soluble	TAK981 (25 uM)	plain RPMI
5.	0 min	surface	DMSO	plain RPMI
6.	0 min	surface	TAK981 (25 uM)	plain RPMI
7.	15 min	surface	DMSO	plain RPMI
8.	15 min	surface	TAK981 (25 uM)	plain RPMI

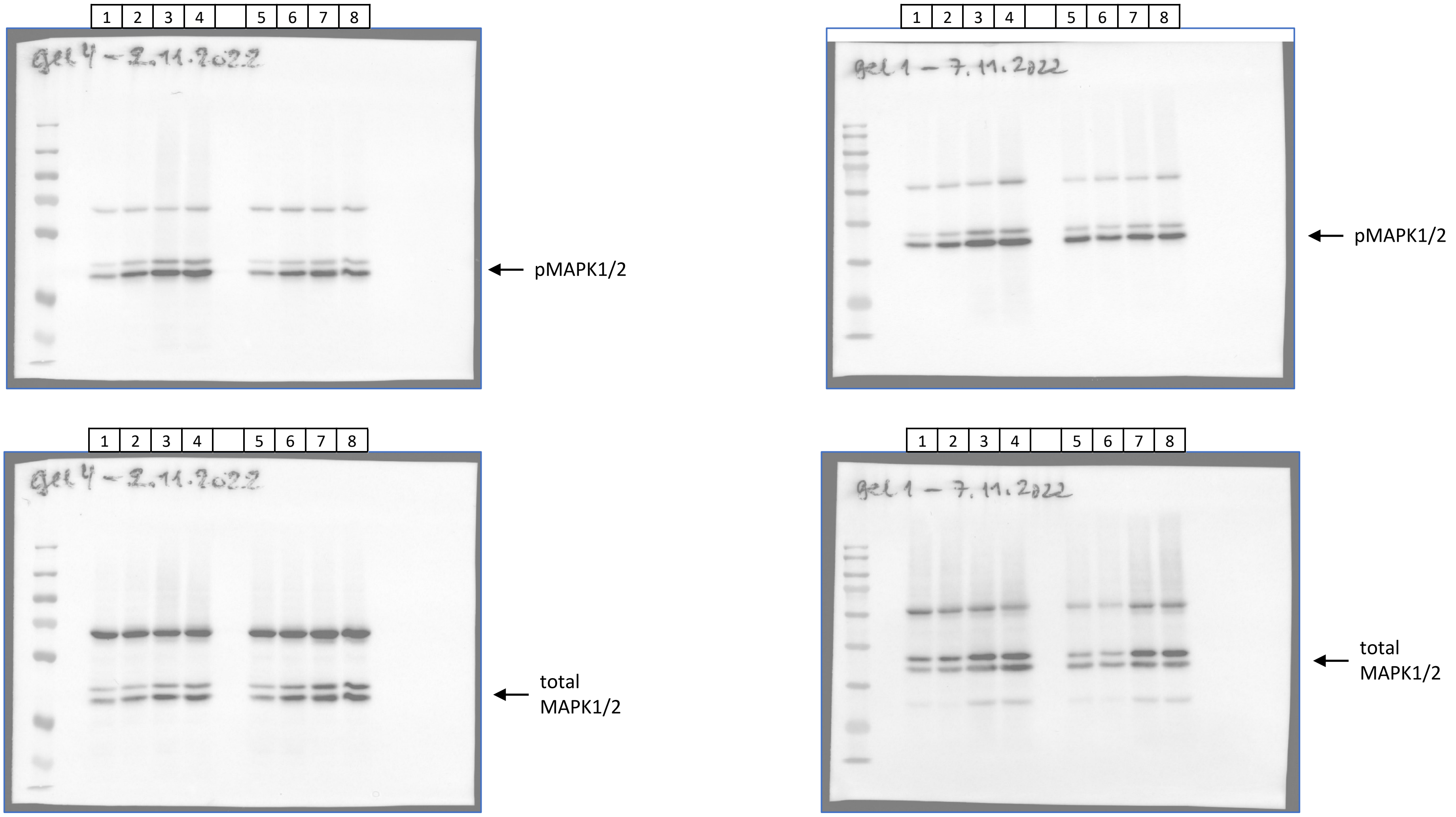

Journal of Cell Science • Supplementary information

Supplementary Fig S7, data integrity, 9/16

Figure 7B, C

A20 D1.3 B cell line

pMAPK1/2; MAPK1/2

1.	0 min	soluble	DMSO	plain RPMI
2.	0 min	soluble	TAK981 (25 uM)	plain RPMI
3.	15 min	soluble	DMSO	plain RPMI
4.	15 min	soluble	TAK981 (25 uM)	plain RPMI
5.	0 min	surface	DMSO	plain RPMI
6.	0 min	surface	TAK981 (25 uM)	plain RPMI
7.	15 min	surface	DMSO	plain RPMI
8.	15 min	surface	TAK981 (25 uM)	plain RPMI

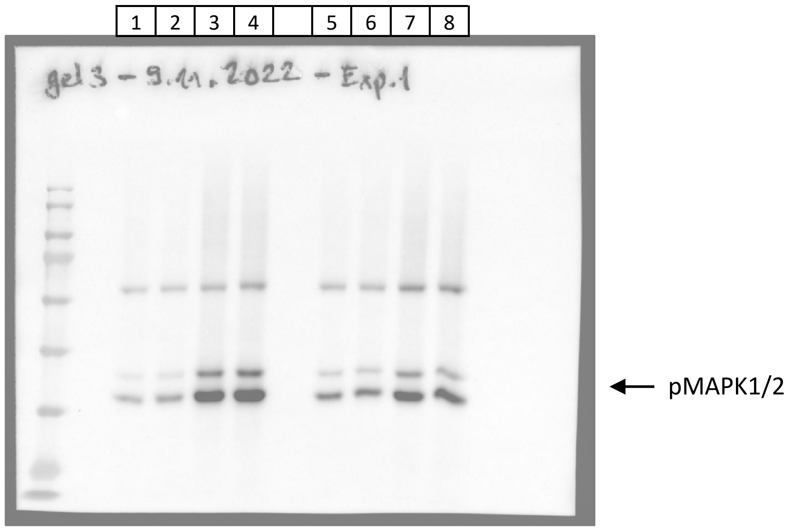

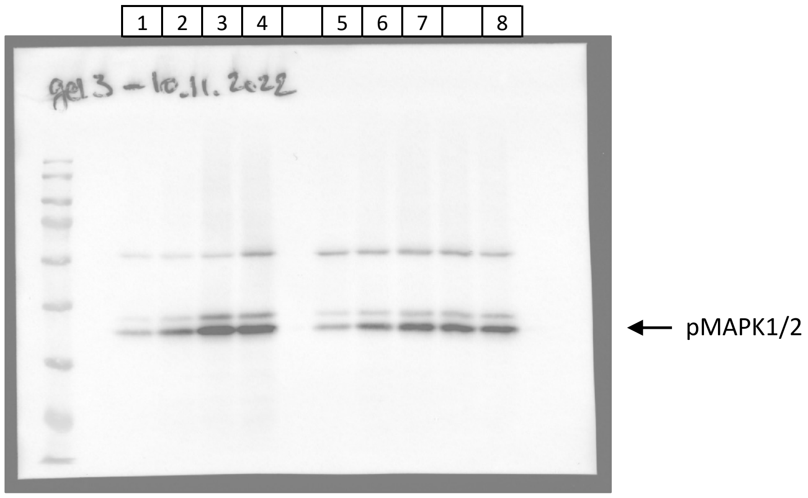

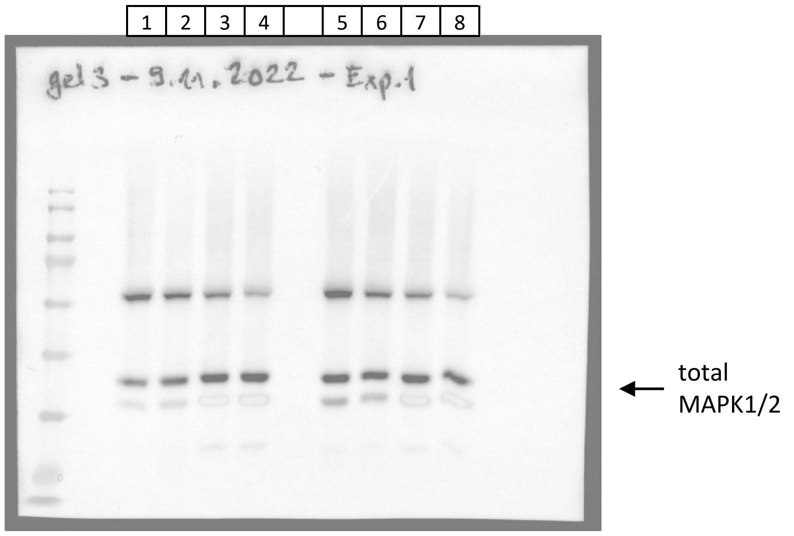

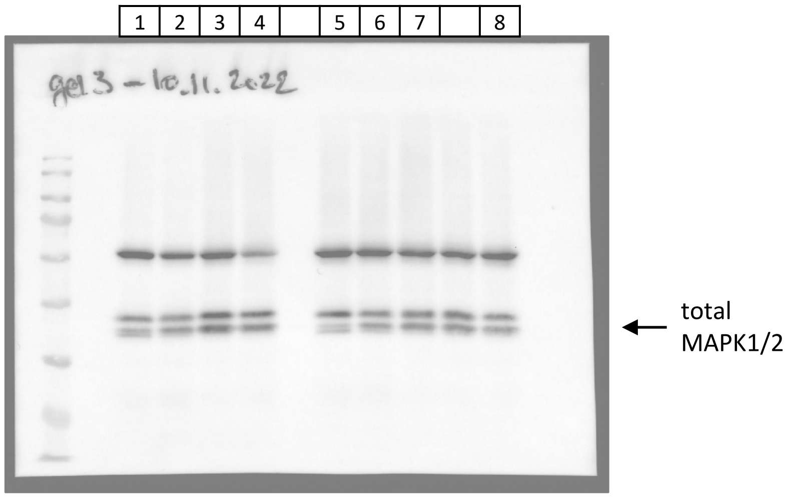

Journal of Cell Science • Supplementary information

Supplementary Fig S7, data integrity, 10/16

Figure 7B, C

A20 D1.3 B cell line

pSyk; Syk

1.	0 min	soluble	DMSO	plain RPMI
2.	0 min	soluble	TAK981 (25 uM)	plain RPMI
3.	15 min	soluble	DMSO	plain RPMI
4.	15 min	soluble	TAK981 (25 uM)	plain RPMI
5.	0 min	surface	DMSO	plain RPMI
6.	0 min	surface	TAK981 (25 uM)	plain RPMI
7.	15 min	surface	DMSO	plain RPMI
8.	15 min	surface	TAK981 (25 uM)	plain RPMI

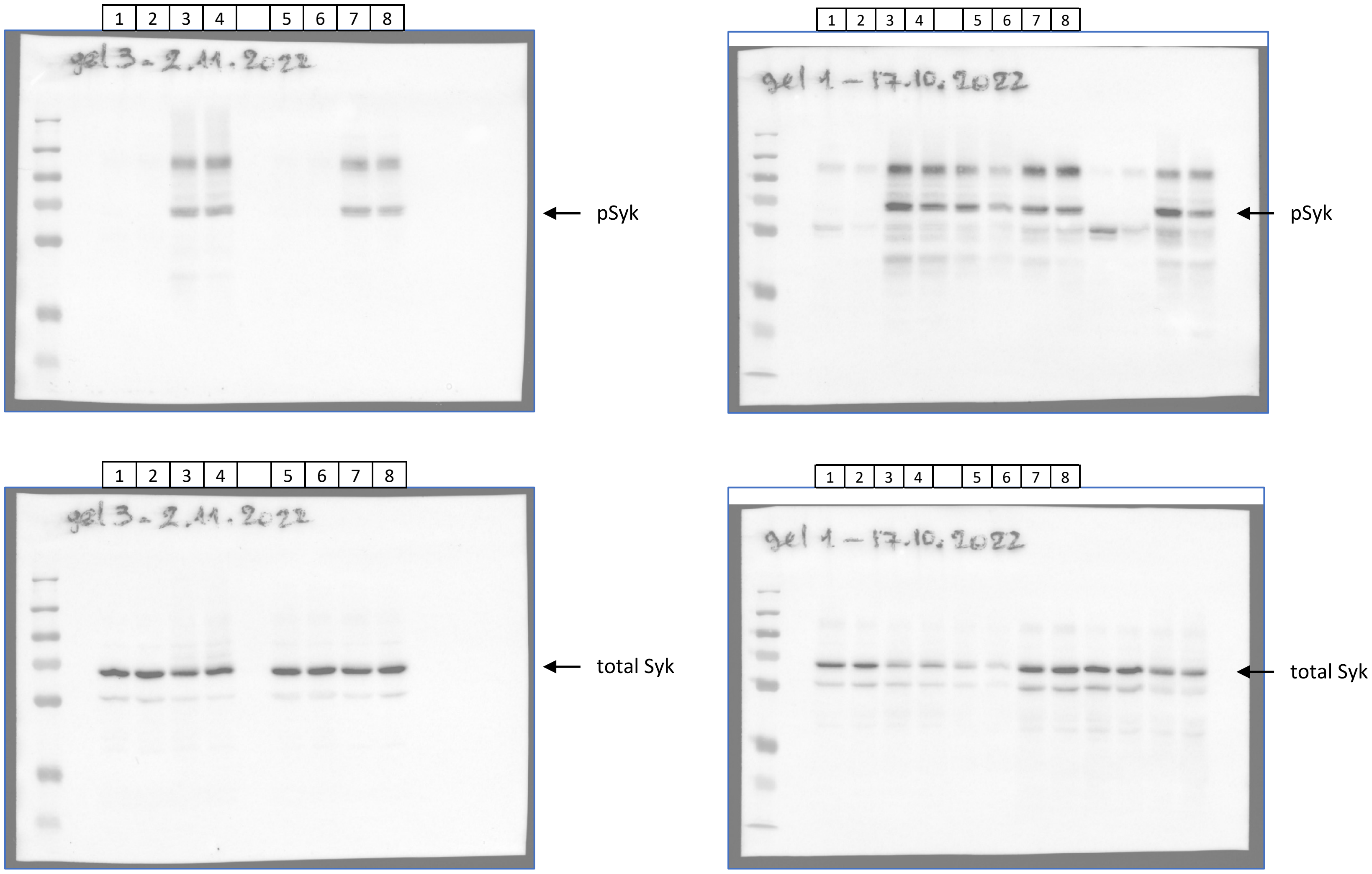

Journal of Cell Science • Supplementary information

Supplementary Fig S7, data integrity, 11/16

Figure 7B, C

A20 D1.3 B cell line

pSyk; Syk

1.	0 min	soluble	DMSO	plain RPMI
2.	0 min	soluble	TAK981 (25 uM)	plain RPMI
3.	15 min	soluble	DMSO	plain RPMI
4.	15 min	soluble	TAK981 (25 uM)	plain RPMI
5.	0 min	surface	DMSO	plain RPMI
6.	0 min	surface	TAK981 (25 uM)	plain RPMI
7.	15 min	surface	DMSO	plain RPMI
8.	15 min	surface	TAK981 (25 uM)	plain RPMI

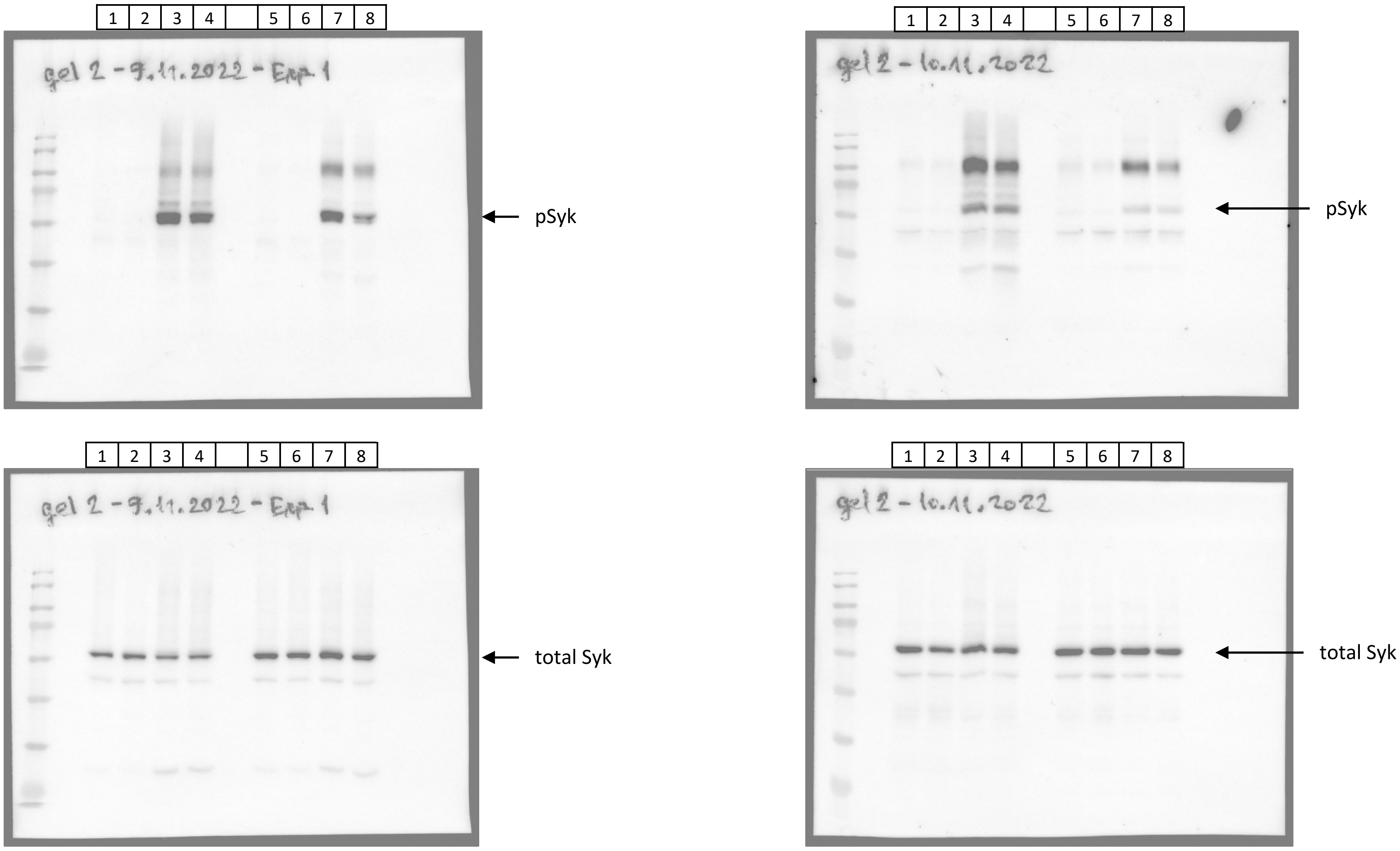

Journal of Cell Science • Supplementary information

Supplementary Fig S7, data integrity, 12/16

Figure 7D, E

primary B cells

pAkt substrate

1.	0 min	soluble	DMSO	plain RPMI
2.	0 min	soluble	TAK981 (25 uM)	plain RPMI
3.	15 min	soluble	DMSO	plain RPMI
4.	15 min	soluble	TAK981 (25 uM)	plain RPMI
5.	0 min	surface	DMSO	plain RPMI
6.	0 min	surface	TAK981 (25 uM)	plain RPMI
7.	15 min	surface	DMSO	plain RPMI
8.	15 min	surface	TAK981 (25 uM)	plain RPMI

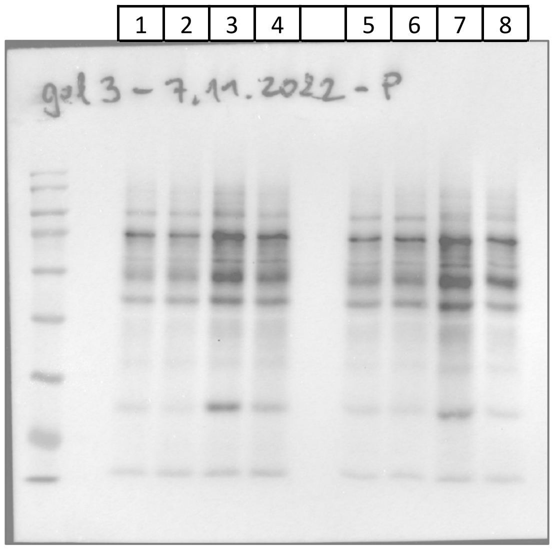

pAkt substrate

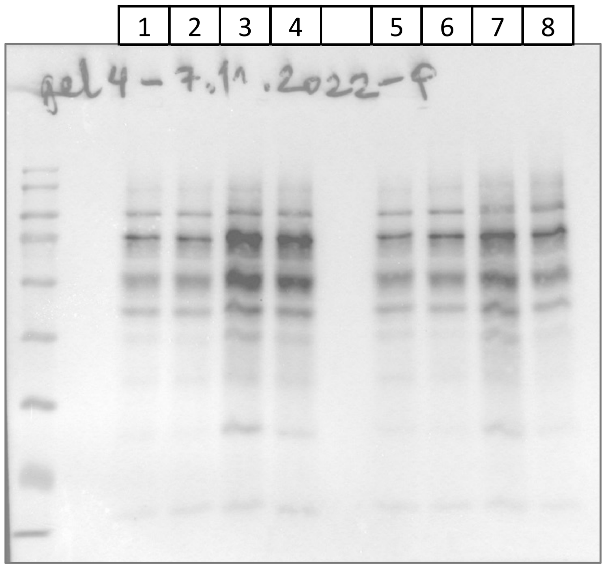

pAkt substrate

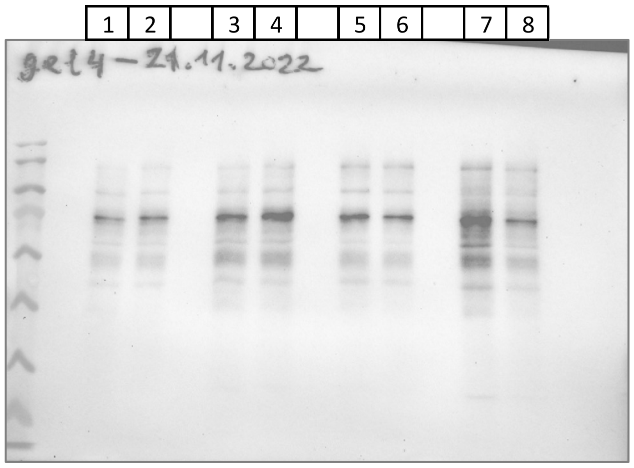

pAkt substrate

Journal of Cell Science • Supplementary information

Supplementary Fig S7, data integrity, 13/16

Figure S1C

pTyr levels
in raft-APEX2 transfected and non-transfected cells

1.	non-activated	APEX2
2.	non-activated	non-transfected
3.	activated	APEX2
4.	activated	non-transfected

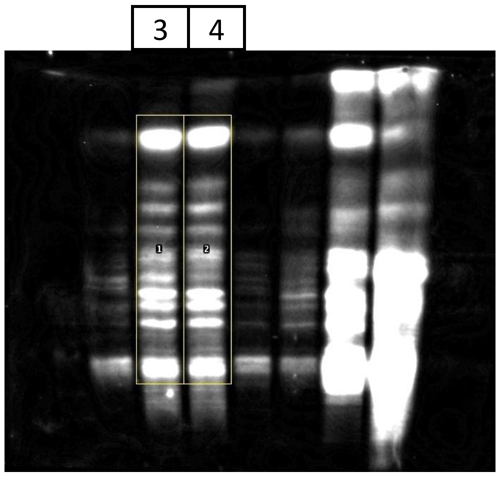

pTyr

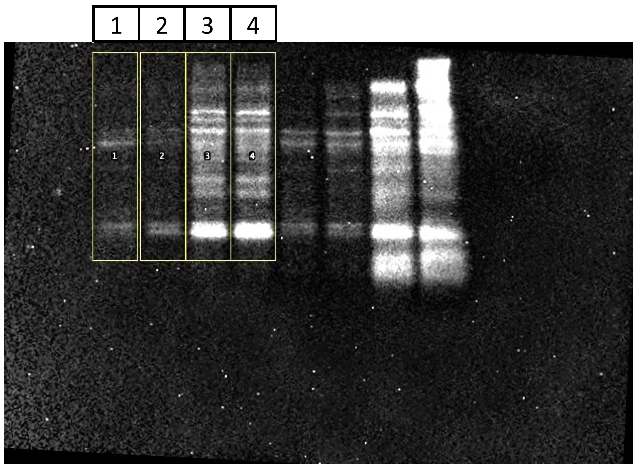

pTyr

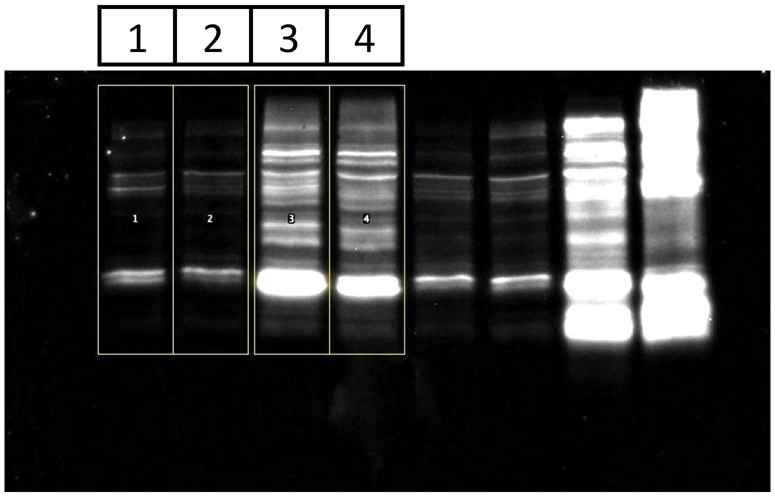

pTyr

Journal of Cell Science • Supplementary information

Supplementary Fig S7, data integrity, 14/16

Figure S1G-H pTyr levels, H_2O_2 titration

1. PBS
2. 0.1 mM H2O2
3. 1 mM H2O2
4. 2 mM H2O2
5. 5 mM H2O2
6. 10 mM H2O2

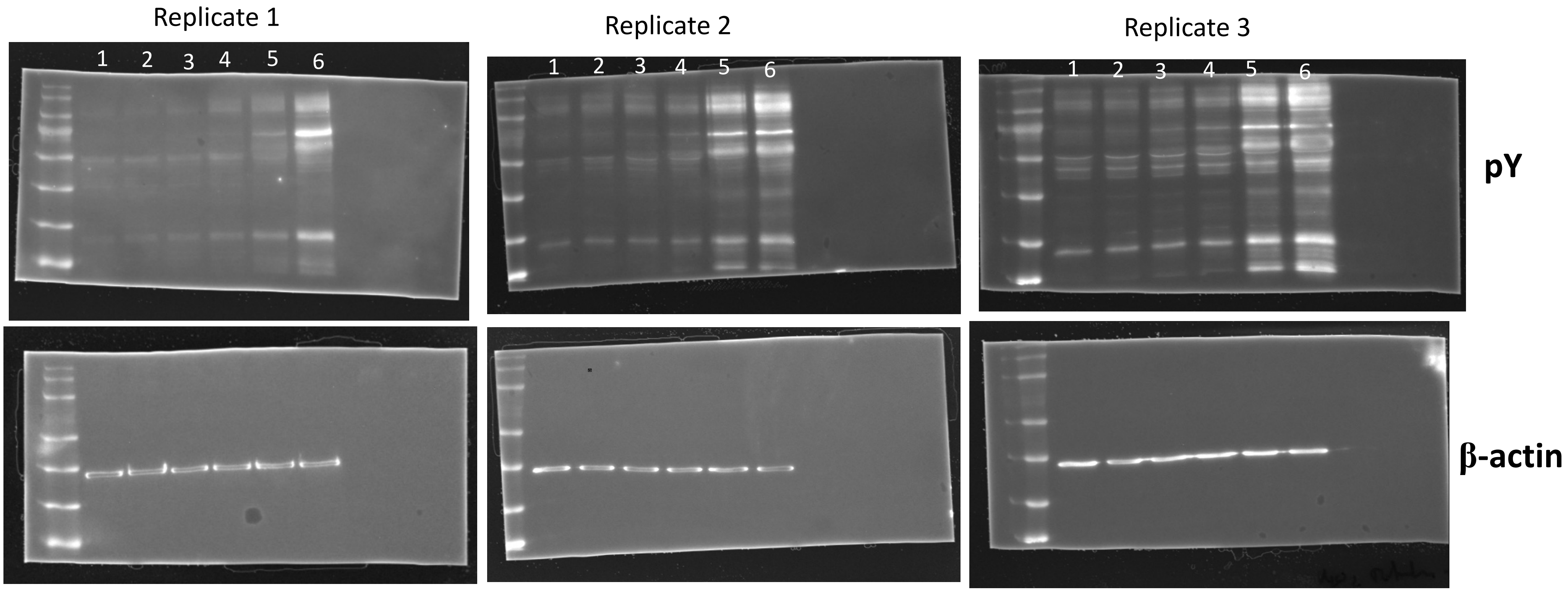

Supplementary Fig S7, data integrity, 15/16

Figure S1I-J pTyr levels, H_2O_2 vs a-IgM activation in raft-APEX2 cells

1. PBS
2. 10 ug/ml of F(ab')2 IgM – 10 min
3. 5 mM H2O2 – 1 min

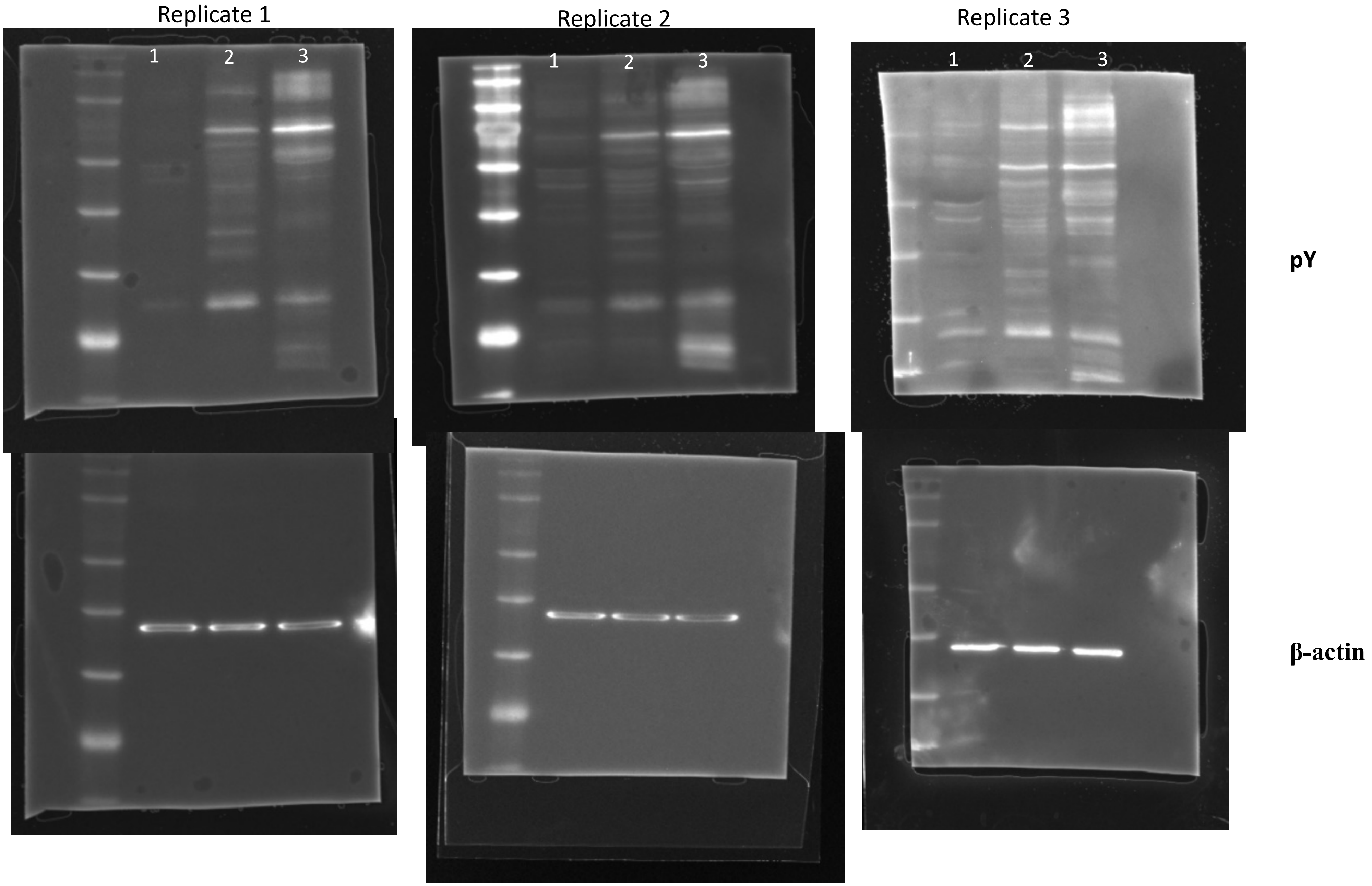

Journal of Cell Science • Supplementary information

Supplementary Fig S7, data integrity, 16/16

Figure S2C-D

Total Syk and pSyk levels
in raft-APEX2 transfected and non-transfected cells

1: Unstimulated raft-APEX2
2: $F(ab')_2$ IgM raft-APEX2
3: Unstimulated non-transf
4: $F(ab')_2$ IgM non-transf

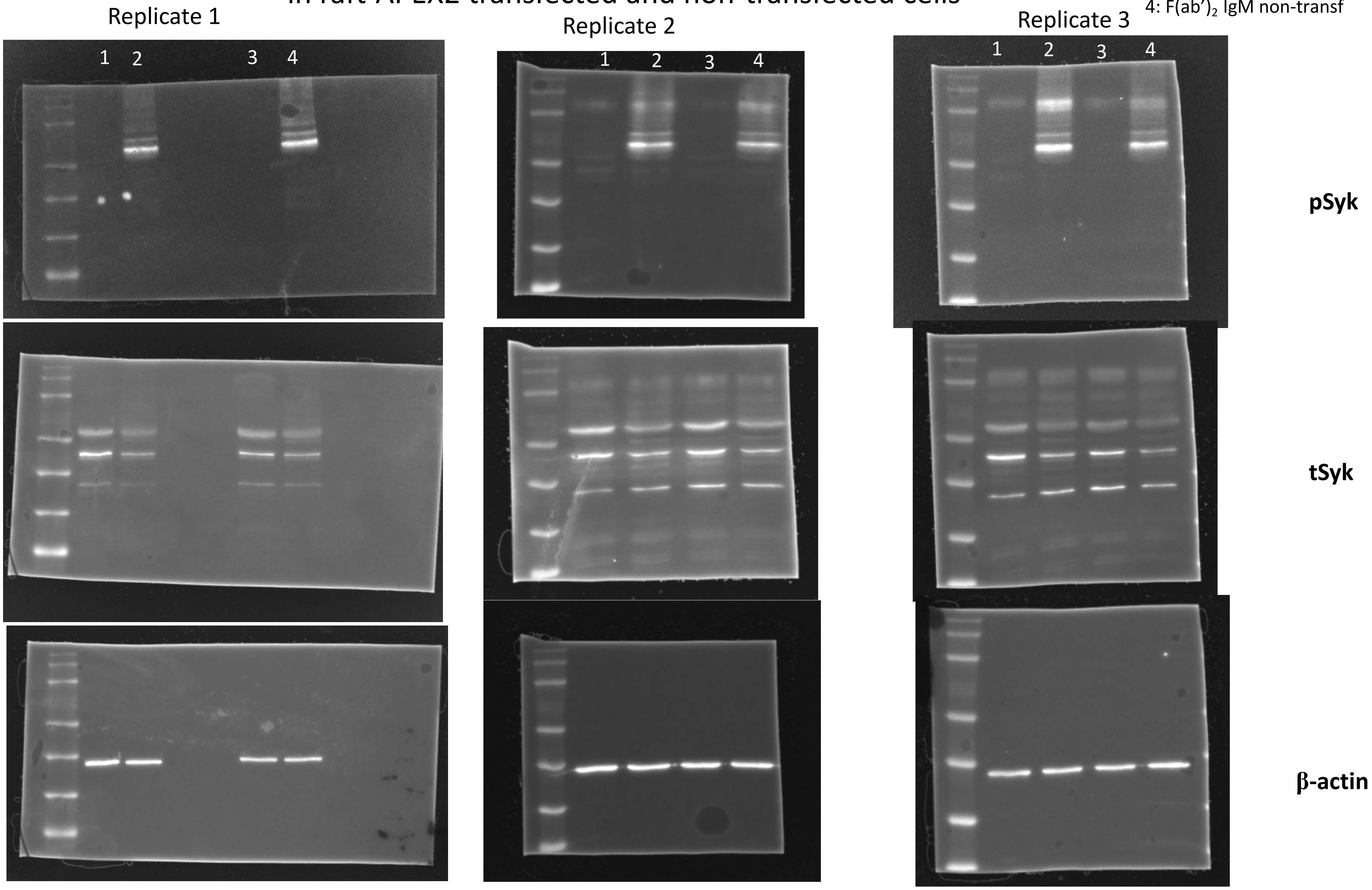

Journal of Cell Science • Supplementary information

Table S1. Mass spectrometry data from raft-APEX2 expressing A20 B cells. A) MaxQuant output data **B)** Proteins identified with high confidence in the whole data set. **C)** List of proteins exclusively identified in resting cells. **D)** Proteins exclusively identified in cells activated with anti-IgM surrogate antigen.

Click here to download Table S1

Table S2. List of proteins defined as lipid raft-resident in B cells.

Click here to download Table S2

Table S3. Differential expression analysis comparing resting and activated B cells. A) List of proteins identified that were used for the differential expression analysis. **B)** List of proteins that show significant enrichment (adjusted p-values ≤ 0.05) at any given conditions and have $\log_2$ fold change ≥ 1.5.

Click here to download Table S3

Table S4. Identified proteins linked to B cell activation and endocytosis. A) List of proteins identified within the GO term "B cell activation processes" (GO:0042113). **B)** List of proteins identified that within the GO term "Endocytosis processes" (GO:0006897).

Click here to download Table S4
