# Supplementary material for: B cell receptor-induced protein dynamics and the emerging role of SUMOylation revealed by proximity proteomics
Source: J Cell Sci. 2023 Aug 8;136(15):jcs261119. doi: 10.1242/jcs.261119 (PMC10445728; doi:10.1242/jcs.261119)
Supplement: Supplementary file 1 [file joces-136-261119_review_history.pdf]

B cell receptor-induced protein dynamics and the emerging role of SUMOylation revealed by proximity proteomics

Luqman O. Awoniyi, Diogo M. Cunha, Alexey V. Sarapulov, Sara Hernández-Pérez, Marika Runsala, Blanca Tejeda-González, Vid Šuštar, M. Özge Balci, Petar Petrov and Pieta K. Mattila

DOI: 10.1242/jcs.261119

Editor: Daniel Billadeau

Review timeline

Submission to Review Commons:	21 June 2021
Submission to Journal of Cell Science:	28 February 2023
Editorial decision:	21 March 2023
First revision received:	20 June 2023
Accepted:	3 July 2023

Reviewer 1

Evidence, reproducibility and clarity

This is an interesting study identifying membrane RAFT associated proteins in a mouse B cell line, before and after BCR stimulation, using a proximity biotinylation method. This method relies on the expression of an biotinylating enzyme APEX2 with a RAFT targeting domain that is activated by H2O2 addition. The system is well controlled by comparing transfected to non-transfected cells and by titrating H2O2 etc. The expected anti-IgM induced recruitment of the BCR to the RAFT domain is well documented in this system. The authors identify by proteome analysis over 1600 proteins in proximity to the membrane- targeted APEX2, most of which are constitutively labelled, i.e. do not change upon BCR stimulation. Only a minority of proteins (less than 100) changes dynamically between resting and stimulated state. Some results are surprising, as the authors discuss, as the known players of the BCR signalling pathway hardly change in their Raft association. Interesting is also the exclusion of signalling proteins such as Btk, BLNK and Ig-alpha/Ig-beta from BCR clusters upon activation. The strength of the system is that the APEX2 system causes a biotinylation with 1 min, which is an advantage to other systems and that the authors analyse 3 time points after BCR stimulation.
The data are thoroughly analysed and discussed. The following points should be addressed:

1. Why did the authors not use the HEL antigen to stimulate their cells, as the A20 line expresses a Ag-specific BCR? Would this not be more physiological than Fab2-anti IgM?
2. Many proteins of pathways like RNA transport, Spliceosome, mRNA surveillance, mismatch repair etc. are identified. Although the authors try to explain some of these data, they should also consider unspecific labelling or unspecific enrichment of these proteins which have nothing to do with raft association. This should be more openly discussed.
3. The authors follow up two proteins that dynamically change during activation, Golga3 and Vti1b, and demonstrate their membrane association upon activation. This is of course relevant. What is missing, is some genetic studies. CRISPR-mediated KO is not difficult to do in cell lines. Have the authors produced such mutants for these two genes and analysed possible phenotypes in BCR signalling or other aspects? This would certainly strengthen the study.

© 2023. Published by The Company of Biologists under the terms of the Creative Commons Attribution License (https://creativecommons.org/licenses/by/4.0/).

Significance

This is a unique approach to globally identify proteins associated with the BCR in Rafts upon BCR stimulation. Comparable studies with other methods have been published before for B cell lines. Gupta et al. used quantitative proteomics of isolated RAFT-associated proteins before and after BCR stimulation. They also found that the association of most proteins to RAFTs was not changed after BCR stimulation. Saeki et al. used a proteomic approach in another B cell line to identify RAFT associated proteins, but without comparing stimulated to unstimulated cells. The approach used here has the advantage of not selecting only membrane bound proteins, but equally identifiying cytosolic proteins in vicinity to the Raft as well. Furthermore, dynamic changes are better analysed than in the other two studies. Therefore, the findings are relevant and a good advance in the field.

Reviewer 2

Evidence, reproducibility and clarity

Summary

Awoniyi et al. utilizes APEX2-mediated proximity proteomics to investigate the protein composition of lipid rafts and their dynamics in the context of B cell receptor (BCR) signaling. The authors add a 7 amino acid guide sequence to an APEX2-mCherry construct to specifically target the fusion protein into lipid raft plasma membrane domains and thereby spatially label and identify contained proteins. While a larger number of lipid raft-related proteins were verified, the study focuses on a smaller subset that is proposed to be specifically related to BCR signaling. Unexpectedly, this approach suggests key players of BCR signaling to be excluded from lipid rafts in an inducible manner. Finally, two of the identified proteins, Golga3 and Vti1b, were further investigated by immunofluorescence microscopy. Since both proteins are shown to be recruited to the plasma membrane and to colocalize with antigen, the authors propose Golga3 and Vti1b as novel targets of BCR activation and drivers of subsequent antigen internalization.

Major Comments

- A major claim of this study is that the majority of BCR signaling proteins (including CD79a and CD79b as parts of the BCR as well as BLNK) get excluded from lipid rafts upon stimulation. Moreover, many components of the endocytosis/vesicle trafficking pathways have been identified and the authors raise interesting points regarding the BCR as signaling platform versus the BCR as antigen internalization complex. This is intriguing and could even be explored further (e.g. by presenting Figure S3 in the main manuscript). However, the claim that Vti1b and Golga3 (and possibly Kif20) play key roles in the endocytic processes underlying BCR/antigen endocytosis and subsequent processing needs further verification e.g. by gene targeting experiments. In its present form, the manuscript links these proteins to B cell activation but does not convincingly back up the implied functional relevance to antigen/BCR endocytosis and/or trafficking leading to antigen presentation via MHC II.
- It should be explained why the proteomic experiments were conducted using anti-IgM antibodies as opposed to the more physiological stimulation via HEL antigen, used for the microscopy studies.
- Even though it is the central approach, the number of figures derived from the APEX2 proteomic experiments is quite high and should be condensed. For example, Figures 4 and 5 could be merged.
- Figure 1D/E and Figure S1C seem to be the same pictures.
- In contrast to Golga3, Vti1b is not mentioned in Figure 4 and the authors should explain why this particular protein was chosen for further investigation among all others (as opposed to proteins enriched upon anti-IgM treatment).
- In Figures 6 and S4, the most apparent changes in Golga3 staining appear to be the increased (cytoplasmic and peripheral) vesicle size and intensity. For the analysis and quantitation of peripheral Golga3 staining, a tubulin-based masking algorithm was used to segment the image. This raises three concerns: 1) The tubulin staining that was used for masking appears to be

© 2023. Published by The Company of Biologists under the terms of the Creative Commons Attribution License (https://creativecommons.org/licenses/by/4.0/).

rather blurry and the expected microtubule network is barely visible.
2) More information is needed on how the masking algorithm treated Golga3 vesicles touching the mask border. Based on the images in S4, there seems to be substantial overlap between (visibly peripheral) Golga3 vesicles and tubulin, so this will likely have an impact on quantification results.
3) Authors should comment on the overall increase in Golga3 upon activation.

Minor Comments

- While the APEX2 construct is globally targeted into the lipid raft environment, the study uses this approach to investigate proteins that are in the proximity of the IgM-BCR. The authors mention in the discussion that there have been "challenges" to target the BCR directly. It may be beneficial to briefly discuss those problems the authors have been facing with.
- Figure 5B: It will be informative to show the BCR-induced (fold change) enrichment of Golga3 and Vti1b (and Kif20) in relation to IgM /kappa LC and the "classical" BCR signaling- related players.
- Figure 6AB: Please clearly indicate that unmasked images are displayed, but masked images were quantified for Golga3 staining.
- Figure 6CD: Since the quantification protocol of vesicle positioning involves nuclear staining, please depict respective DAPI stainings.
- Figure S1D: It should be indicated that AF633-streptavidin was used for the flow cytometry experiment in Figure S1D (x axis).

Significance

Overall, the presented study offers an interesting approach and provides a novel, unbiased view on BCR-mediated lipid raft dynamics. The method is appealing in its technicality and its presentation, and hence, might attract the attention of a larger community working on plasma membrane localization of signaling platforms. It proposes two candidate signaling proteins and verifies their BCR-dependent colocalization with lipid rafts and antigen. While Golga3 and Vti1b are novel and interesting target proteins in the context of BCR activation, a functional assessment of these proteins is not presented. Certainly, the article would greatly benefit from a follow-up investigation on the functional/physiological relevance of the proposed players. As it stands, the manuscript largely remains on the level of exploration.

Reviewer 3

Evidence, reproducibility and clarity

Review Commons recommends including the following components in referee reports:

1. Evidence, reproducibility and clarity

Summary:

Provide a short summary of the findings and key conclusions (including methodology and model system(s) where appropriate). Please place your comments about significance in section 2.

The manuscript by Awoniyi et al is an elegant study that addresses the protein composition of the lipid rafts upon BCR activation. The authors use an elegant system, employing the enzyme ascorbate peroxidase (APEX2), which in cellulo generates short-lived biotin radicals, that in turn randomly bind to proteins in their vicinity (10-20 nm) within 1 min. APEX2 is furthermore fused with the 7-amino acid sequence MGCVCSS, which allows its targeting to lipid rafts (Raft-APEX2) and with an mCherry marker. Using modern microscopy methods as well as quantitative mass-spectrometry proteomics, the authors provide a spatially and temporally resolved dynamic insight into the changes within the lipid raft and. are able to enrich multiple proteins in the lipid rafts previously not associated with BCR signaling. Furthermore, they identify Golga3 and Vti1b as proteins proximally responding to BCR activation possibly enabling vesicle transport.

© 2023. Published by The Company of Biologists under the terms of the Creative Commons Attribution License (https://creativecommons.org/licenses/by/4.0/).

The manuscript is generally well written, the study is well-conceived and well-controlled. Nevertheless, the authors may answer some important questions (see below)

Major comments:

• Are the key conclusions convincing?

Yes, the key conclusions of the study are convincing and based on elegant experiments

• Should the authors qualify some of their claims as preliminary or speculative, or remove them altogether?

• Would additional experiments be essential to support the claims of the paper? Request additional experiments only where necessary for the paper as it is, and do not ask authors to open new lines of experimentation.

• In Figure 2, the HEL-specific A20 B cells are stimulated with anti-IgM F(ab)'2. While, beyond a doubt, anti-IgM F(ab)'2 is a potent stimulus, which triggers BCR signaling activation, I am curious why the authors chose it over the HEL antigen.

• Describing figures 4 and 5, the authors state that they did not identify prominent BCR signaling pathway regulators. My major concern here is that the authors employ cancerous B cells for their analyses. The lipid raft composition and proteins recruited to the rafts in these cells may vary from those in primary wild-type B cells. While the authors do keep in mind that the signaling protein composition may vary between cell lines, it may vary even more between lymphoma B cell line and primary wild-type cells. Therefore, it may be beneficial to verify the expression of the "unexpected" proteins, such as Golga3, Kif20a, and Vtib1b in primary cells using immunofluorescence analyses similar to the ones presented in Fig 6.

• The authors mention in the discussion, that Syk was not identified in their data set. This is surprising as Syk has been attributed with an important role in the proximal BCR signalling (Kulathu et al, https://doi.org/10.1111/j.1600-065X.2009.00837.x)

• Would it be possible to detect Syk using the immunofluorescence technique from Figure 6?

• Additionally, as stated in the text, A20 cells express endogenous IgG2a. Have the authors tried to conduct similar experiments stimulating with anti-IgG antibodies instead of anti-IgM F(ab)'2?

• Have the authors tried to co-express the IgD-BCR to mimic mature peripheral B cells?

• Are the suggested experiments realistic in terms of time and resources? It would help if you could add an estimated cost and time investment for substantial experiments.

I cannot estimate the cost but if conducted, some experiments might take several months

• Are the data and the methods presented in such a way that they can be reproduced?
Yes
• Are the experiments adequately replicated and statistical analysis adequate? Yes

Minor comments:

• Specific experimental issues that are easily addressable.

• Are prior studies referenced appropriately? Yes

• Are the text and figures clear and accurate?

Yes, but, if possible, please provide the data instead of writing "data not shown"

© 2023. Published by The Company of Biologists under the terms of the Creative Commons Attribution License (https://creativecommons.org/licenses/by/4.0/).

• Do you have suggestions that would help the authors improve the presentation of their data and conclusions?

Fig. 1D and Supp. Fig. S1C: the authors state that after IgM cross-linking, the non- transfected and Raft-APEX2-transfected cells showed "indistinguishable" p-Tyr levels. From my perspective, the Raft-APEX2-transfected cells show higher levels of p-Tyr. It is possible to quantify it?

Some paragraph titles are very short and descriptive (e.g. Proteomic analysis, membrane-proximal proteome etc.). It could improve the reading if the paragraph titles consisted of respective key findings

Significance

2. Significance

• Describe the nature and significance of the advance (e.g. conceptual, technical, clinical) for the field.

• This study dissects in a spatio-temporal manner the early events upon BCR stimulation and the enrichment of various proteins in the vicinity of lipid rafts. While conceptually not novel, the study provides novel methodology to address this question. This is technically relevant and worth to be published after a major revision.

• Place the work in the context of the existing literature (provide references, where appropriate).

This study prominently overlooks a bulk of literature that supports the BCR dissociation activation model and does not comment that (reviewed in Maity et al, Volume 1853, Issue 4, April 2015, Pages 830-840)

• State what audience might be interested in and influenced by the reported findings.

The findings of this manuscript are specifically interesting for researchers who study early events of B cells activation, specifically the changes in the membrane composition and early BCR signalling

• Define your field of expertise with a few keywords to help the authors contextualize your point of view. Indicate if there are any parts of the paper that you do not have sufficient expertise to evaluate.

Immunology, Molecular and Cell Biology

Referee Cross-commenting**

I agree totally that "the article would greatly benefit from a follow-up investigation on the functional/physiological relevance of the proposed players", however only if this is easily done with the CRISP mediated knock out as mentioned by both reviewers. In addition it s interesting to see data on cells stimulated with the antigen instead of anti IgM fab'2.

Author response to reviewers' comments

[Please use this template only if the submitted manuscript should be considered by the affiliate journal as a full revision in response to the points raised by the reviewers.

If you wish to submit a preliminary revision with a revision plan, please use our "Revision Plan"

© 2023. Published by The Company of Biologists under the terms of the Creative Commons Attribution License (https://creativecommons.org/licenses/by/4.0/).

template. ***It is important to use the appropriate template to clearly inform the editors of your intentions.]***

1. General Statements [optional]

This section is optional. Insert here any general statements you wish to make about the goal of the study or about the reviews.

We find the reviewers' comments reasonable and fair. We see two main points of criticisms as:

1) *the choice of used antigen (surrogate antigen instead of a real epitope antigen).* While it would be interesting and informative to compare the widely used anti-IgM surrogate antigen to real epitope antigen, this is beyond the current manuscript. As also explained in our response to the *Reviewer 1, comment 1.*, the technical mass spectrometry analysis in this manuscript has been performed already couple of years ago. Since that time, our mass spectrometry analyser system has been updated and to allow reliable comparison to the epitope antigen data, also the old experiments should be repeated, which would ultimately lead to very high number of mass spectrometry samples and higher resources that we have been able to allocate. Unfortunately, we have not had the required resources to add such a sizable extension to our manuscript. **As the vast majority of BCR activation studies in the field rely on the use of surrogate antigen, our dataset is still of great value and reveals various novel players in B cell activation.**

2) *the lack of functional data on the identified hits.* We went on to further explore the phenotype of the SNARE Vti1b, which was one of the hits validated in this manuscript when it was reviewed for Review Commons. However, while the localization of Vti1b to the sites of BCR activation were confirmed, it was found functionally redundant (likely due to the homologous SNARE Vti1a). We considered that these negative results would not improve our current manuscript, we took off the Vti1b data (now published separately: Music et al, "The SNARE protein Vti1b is recruited to the sites of BCR activation but redundant for antigen internalisation, processing and presentation" *Frontiers in Cell and Developmental Biology,* 2022). To investigate other hits from our screen, we picked SUMO and now **show, for the first time, that there is notable SUMOylation occurring at the sites of BCR activation and show functional role of SUMOylation in BCR signalling in primary mouse B cells.**

2. Point-by-point description of the revisions

This section is mandatory. Please insert a point-by-point reply describing the revisions that were *<u>already carried out and included</u>* *in the transferred manuscript.*

Reviewer 1.

1. Why did the authors not use the HEL antigen to stimulate their cells, as the A20 line expresses a Ag-specific BCR? Would this not be more physiological than Fab2-anti IgM?

- This valid point was raised by all the reviewers. Our plan was, and still is, to use both anti-IgM and HEL in our APEX2-assay and use the resulting information to ask another outstanding question in the field: how BCR-delivered signals differ when using surrogate or real epitope- antigen? Unfortunately, at the time, due to practical reasons we were not able to perform the APEX2 experiments with a wider range of conditions, because of the share extent of these experiments and limited resources. Large amounts of cells for each condition and various controls for each experiment are required for these experiments, leading to practical challenges in commanding experiments as well as increasingly high costs of mass spectrometric analyses. The above reasons forced our decision to start with the less extensive setup and use only the surrogate antigen (anti-IgM) stimulation, as this type of activation is most widely used in the existing literature.
While analyzing this data, the Covid19 pandemic kicked in and we were forced to cease all of the experimental work on this project, and also lost critical personnel on this project, so the decision was made to stick with the IgM data only, as we could not plan further experiments. Since then also our proteomics facility has updated their machine setup, so it would not be possible to generate new data that we could directly combine with the existing dataset. **For a comparative analysis of**

© 2023. Published by The Company of Biologists under the terms of the Creative Commons Attribution License (https://creativecommons.org/licenses/by/4.0/).

HEL vs anti-IgM activation response, a completely new, large dataset would be required. We feel that this is beyond the scope of this manuscript, but agree that this kind of analysis would be very important to do in the future.

2. Many proteins of pathways like RNA transport, Spliceosome, mRNA surveillance, mismatch repair etc. are identified. Although the authors try to explain some of these data, they should also consider unspecific labelling or unspecific enrichment of these proteins which have nothing to do with raft association. This should be more openly discussed.

- This is an important point. In our setup, we used two controls: samples without H_2O_2 and samples without biotin-phenol to control for baseline activity of the APEX2 and endogenous biotinylation respectively. At the same time, these controls help to filter out proteins that bind to the streptavidin beads in an unspecific manner. However, we do not rule out a possibility of unspecific enrichment in the pull-down and, furthermore, there can be background biotinylation generated from a “nonlocalized” pool of APEX2 that would include the enzyme that is just being produced by the ribosome or is on the way to the plasma membrane. We have now added discussion on these possibilities in the text.

3. The authors follow up two proteins that dynamically change during activation, Golga3 and Vti1b, and demonstrate their membrane association upon activation. This is of course relevant. What is missing, is some genetic studies. CRISPR-mediated KO is not difficult to do in cell lines. Have the authors produced such mutants for these two genes and analysed possible phenotypes in BCR signalling or other aspects? This would certainly strengthen the study.

- Regarding the functional studies, we did continue to study the phenotype in B cells isolated from Vti1b-deficient mice. Unfortunately, we were not able to detect clear defects in B cell activation in Vti1b-deficient primary cells. We also found out that the anti-Vti1b antibodies were not reliable. For these reasons, we decided to remove the data on Vti1b from this manuscript. We were, however, able to validate the localization of Vti1b to the sites of BCR activation by a GFP-fusion construct and report our findings in another study (Music et al, "The SNARE protein Vti1b is recruited to the sites of BCR activation but is redundant for antigen internalisation, processing and presentation" *Frontiers in Cell and Developmental Biology*, 2022).

- Instead, we picked another hit from the dataset, SUMO, and have now added demonstration of SUMOylation occurring at various sites of BCR activation, both in a B cell line and in primary mouse B cells. Notably, using an pharmacological **inhibitor of SUMOylation**, we find that particularly in primary mouse B cells, **dynamic SUMOylation is required for BCR signalling through the Akt and MAPK axes.** Due to the high abundance and critical functions of SUMO in nucleus, genetic studies would be highly challenging.

Reviewer 2.

- *A major claim of this study is that the majority of BCR signaling proteins (including CD79a and CD79b as parts of the BCR as well as BLNK) get excluded from lipid rafts upon stimulation. Moreover, many components of the endocytosis/vesicle trafficking pathways have been identified and the authors raise interesting points regarding the BCR as signaling platform versus the BCR as antigen internalization complex. This is intriguing and could even be explored further (e.g. by presenting Figure S3 in the main manuscript). However, the claim that Vti1b and Golga3 (and possibly Kif20) play key roles in the endocytic processes underlying BCR/antigen endocytosis and subsequent processing needs further verification e.g. by gene targeting experiments. In its present form, the manuscript links these proteins to B cell activation but does not convincingly back up the implied functional relevance to antigen/BCR endocytosis and/or trafficking leading to antigen presentation via MHC II.*

- We agree that it is premature to state that Vti1b and Golga3 would play key roles in the processes underlying BCR/antigen endocytosis and stating that has not been our intention. We have, based on our immunofluorescence studies, speculated on a link between antigen-induced endosomal trafficking and Golga3. The data on Vti1b has been now removed from the current manuscript (please, see the answer to Reviewer 1, concern 3). Importantly, we have added

© 2023. Published by The Company of Biologists under the terms of the Creative Commons Attribution License (https://creativecommons.org/licenses/by/4.0/).

validation of a new hit, SUMO. In addition to validating the localization to the sites of BCR activation, we show that **dynamic SUMOylation is required for BCR signalling through the Akt and MAPK axes.**

- *It should be explained why the proteomic experiments were conducted using anti-IgM antibodies as opposed to the more physiological stimulation via HEL antigen, used for the microscopy studies.*

- This is a very valid point. Please, see the answer to Reviewer 1, concern 1.

- *Even though it is the central approach, the number of figures derived from the APEX2 proteomic experiments is quite high and should be condensed. For example, Figures 4 and 5 could be merged.*

- Our aim was to keep different types of analyses in separate images to add clarity. For example, Figure 4 presents the exclusively identified proteins while the Figure 5 presents the enrichment analysis and, thus, they show different hits. In the resubmission phase, we can surely condense/fuse some of the figures if deemed favorable.

- *Figure 1D/E and Figure S1C seem to be the same pictures.*

- We thank Reviewer 2 for this comment. Yes, figure 1D/E and Figure S1C are indeed the same picture, such that the figure S1C shows the whole, uncropped western blot while in figure 1D and E the lanes were cropped for clarity, as the same blot addresses two different experimental questions. We realized that this was not clear in the legend of figure S1C. This point is now clarified in the supplementary figure legend.

- *In contrast to Golga3, Vti1b is not mentioned in Figure 4 and the authors should explain why this particular protein was chosen for further investigation among all others (as opposed to proteins enriched upon anti-IgM treatment).*

- Figure 4 focuses on proteins exclusively identified in certain experimental conditions. Golga3 and Kif20A were the only two proteins identified exclusively in all anti-IgM activated conditions (5, 10 and 15min), as shown in Fig 4A. Vti1b, on the other hand, is among the 1143 proteins identified in all, including non-activated, conditions. However, we have now performed a major revision of the manuscript and removed Vti1b from the manuscript (please, see the answer to Reviewer 1, concern 3).

In Figures 6 and S4, the most apparent changes in Golga3 staining appear to be the increased (cytoplasmic and peripheral) vesicle size and intensity. For the analysis and quantitation of peripheral Golga3 staining, a tubulin-based masking algorithm was used to segment the image. This raises three concerns: 1) The tubulin staining that was used for masking appears to be rather blurry and the expected microtubule network is barely visible.
2) More information is needed on how the masking algorithm treated Golga3 vesicles touching the mask border. Based on the images in S4, there seems to be substantial overlap between (visibly peripheral) Golga3 vesicles and tubulin, so this will likely have an impact on quantification results.
3) Authors should comment on the overall increase in Golga3 upon activation.

- **Concern 1)** The tubulin staining remains blurry because the samples were prepared in conditions where the Golga3 antibody was working the best. The procedure contained methanol-Acetone fixation that is not optimal for microtubules (https://doi.org/10.1186/s13630-017-0045-9) causing some blurriness for the images. Also, one should note that Tubulin channel is not deconvoluted. However, despite of the blurriness, the segmentation based on the tubulin channel was generating a well-defined mask for the cells, which then, followed by defined subtraction, worked well for us to generate a “shell” of the peripheral regions of the cells for the quantitative analysis. **Concern 2)** The final mask of cell periphery is generated by a defined substraction from the preliminary mask created on tubulin signal. So, the vesicles overlapping with tubulin at the cell periphery do get counted in if they remain outside of the “cell periphery mask”. But if a vesicle sits on the mask border, the part of its signal that remains inside the mask would get lost. All the fluorescence signal inside of the “cell periphery mask” is deleted

© 2023. Published by The Company of Biologists under the terms of the Creative Commons Attribution License (https://creativecommons.org/licenses/by/4.0/).

form the analysis. **Concern 3)** Intriguingly, we see a large portion of Golga3 staining appearing dotted inside the nucleus. The amount of this staining in any particular confocal slice largely reflects the area covered by nucleus in that particular slice. So while it appears that the levels of Golga3 are higher in the activated cell in Fig 6, the difference is likely to rather indicate larger nuclear area in that particular slice. We do not know what function Golga3 has in the nucleus but assume that it is likely to be different from what is occurring in the cell periphery. This is an interesting observation that we feel, however, to be outside of the scope of this study.

- *While the APEX2 construct is globally targeted into the lipid raft environment, the study uses this approach to investigate proteins that are in the proximity of the IgM-BCR. The authors mention in the discussion that there have been "challenges" to target the BCR directly. It may be beneficial to briefly discuss those problems the authors have been facing with.*

- We have now added discussion on this to the text. In short, we first intended to fuse APEX2 to BCR via Iga or Igb, and also tried Lyn-APEX2, but were not able to successfully express these constructs in B cell lines. In contrary, the same APEX2-fusion constructs were successfully expressed in fibroblasts suggesting that the problem was specific to B cells.

Figure 5B: It will be informative to show the BCR-induced (fold change) enrichment of Golga3 and Vti1b (and Kif20) in relation to IgM /kappa LC and the "classical" BCR signaling- related players.

- Golga3 is not included in Fig5A or 5C because this figure describes enrichment analysis, which is only possible to perform to proteins identified in the majority of conditions. While Golga3 was not identified in any of the non-activated samples, enrichment or fold-change could not be calculated. The heat map in Fig 5C shows the enrichment profiles of the known BCR signaling proteins. In the manuscript, we have now added validation of SUMO enrichment at the sites of BCR activation and its functional role in BCR signaling. In the new Figure 6, we have now included such a heatmap to show dynamics of various SUMOylation-linked proteins.

- Figure 6AB: Please clearly indicate that unmasked images are displayed, but masked images were quantified for Golga3 staining.

- All the data on Golga3 is now Supplementary Figure S4, and we have now added clarifying text both to the colocalization graph and to the figure legend to clearly state this important point (Suppl Fig S4,B-D).

- *Figure 6CD: Since the quantification protocol of vesicle positioning involves nuclear staining, please depict respective DAPI stainings.*

- This part of the data (Vti1b) has been removed from the current manuscript.

- *Figure S1D: It should be indicated that AF633-streptavidin was used for the flow cytometry experiment in Figure S1D (x axis).*

- We have now indicated AF633-streptavidin in this flow cytometry data chart (now changed to Figure S1E).

Reviewer 3.

- *In Figure 2, the HEL-specific A20 B cells are stimulated with anti-IgM F(ab)'2. While, beyond a doubt, anti-IgM F(ab)'2 is a potent stimulus, which triggers BCR signaling activation, I am curious why the authors chose it over the HEL antigen.*

- We acknowledge this very valid point. Please, see the answer to Reviewer 1, concern 1.

Describing figures 4 and 5, the authors state that they did not identify prominent BCR signaling pathway regulators. My major concern here is that the authors employ cancerous B cells for their analyses. The lipid raft composition and proteins recruited to the rafts in these cells may

© 2023. Published by The Company of Biologists under the terms of the Creative Commons Attribution License (https://creativecommons.org/licenses/by/4.0/).

vary from those in primary wild-type B cells. While the authors do keep in mind that the signaling protein composition may vary between cell lines, it may vary even more between lymphoma B cell line and primary wild-type cells. Therefore, it may be beneficial to verify the expression of the "unexpected" proteins, such as Golga3, Kif20a, and Vtib1b in primary cells using immunofluorescence analyses similar to the ones presented in Fig 6.

- This is an important point. Unfortunately, we have not been able to get the anti-Golga3 antibodies to work in mouse cells and have not been able to find any functional antibody against Kif20a, so we were not able to add data on these proteins in in primary mouse B cells. The data on Vti1b has been omitted from the manuscript (please, see the answer to Reviewer 1, concern 3). Notably, we have now added demonstration of SUMOylation occurring at the sites of BCR activation, and demonstrated its functional role in BCR signalling in primary mouse B cells.

• *The authors mention in the discussion, that Syk was not identified in their data set. This is surprising as Syk has been attributed with an important role in the proximal BCR signalling (Kulathu et al, https://doi.org/10.1111/j.1600-065X.2009.00837.x)*

- This was indeed surprizing. But, notably, if a protein is not identified by mass spectrometry it does not simply mean that the protein is absent. A particular protein can fail detection due to various factors that can be linked to the mass spectrometry analysis itself or, in this case, it might also fail to get biotinylated due to steric obstruction of amino acids targeted by the biotin-phenol radicals or lack of suitable reactive moieties in the protein sequence. This is now better clarified in the text.

• *Would it be possible to detect Syk using the immunofluorescence technique from Figure 6?*
- Yes, we can routinely detect Syk and also phosphorylated Syk in our cell line, both by Western blot and by the immunofluorescence. A Western blot showing this can be added to the supplementary data if deemed useful.

• *Additionally, as stated in the text, A20 cells express endogenous IgG2a. Have the authors tried to conduct similar experiments stimulating with anti-IgG antibodies instead of anti-IgM F(ab)'2?*

- This we have not tried and while an interesting suggestion, we feel this is out of the scope of this manuscript (please, see also the answer to Reviewer 1, concern 1). We would also wish to note here that the APEX2 biotinylation experiments conducted in this study demand significant resources of time and financial budget, which is the reason why we had to limit the numbers of different conditions.

• *Have the authors tried to co-express the IgD-BCR to mimic mature peripheral B cells?*

- This is also an interesting suggestion for future studies. We have not tried it.

Fig. 1D and Supp. Fig. S1C: the authors state that after IgM cross-linking, the non- transfected and Raft-APEX2-transfected cells showed "indistinguishable" p-Tyr levels. From my perspective, the Raft-APEX2-transfected cells show higher levels of p-Tyr. It is possible to quantify it?

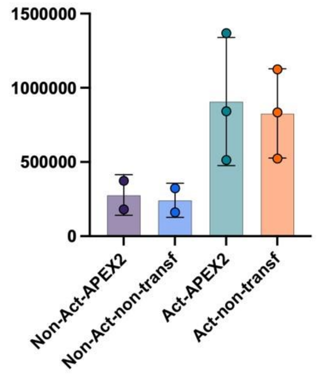

© 2023. Published by The Company of Biologists under the terms of the Creative Commons Attribution License (https://creativecommons.org/licenses/by/4.0/).

- Indeed, in that one Western blot it appears like the APEX2- expressing cells could signal slightly stronger. However, we have repeated this experiment 3 times and now also quantified the blots, and do not detect significant differences (see the graph on the right). In the manuscript, we used the Western blot to simply demonstrate that the expression of APEX2 does not impair BCR signalling. We did not include the quantification in the manuscript but can do that if so preferred.

Some paragraph titles are very short and descriptive (e.g. Proteomic analysis, membrane-proximal proteome etc.). It could improve the reading if the paragraph titles consisted of respective key findings.

- We thank the reviewer for this comment and have now reconsidered the paragraph titles and added more message to them.

- This study prominently overlooks a bulk of literature that supports the BCR dissociation activation model and does not comment that (reviewed in Maity et al, Volume 1853, Issue 4, April 2015, Pages 830-840)

- As the time points in our data are from 5 min onwards, and only report about the raft-environment, our data is not designed to probe for different activation triggering models in the required accuracy. However, we have now added discussion on the data in regards to BCR dissociation activation model in our revised manuscript.

First decision letter

MS ID#: JOCES/2023/261119

MS TITLE: BCR-induced protein dynamics and the emerging role of SUMOylation revealed by proximity proteomics

AUTHORS: Luqman O. Awoniyi, Alexey V. Sarapulov, Sara Hernández-Pérez, Marika Runsala, Diogo M. Cunha, Blanca Tejeda-González, Vid Šuštar, M. Özge Balci, Petar Petrov and Pieta K Mattila
ARTICLE TYPE: Research Article

We have now reached a decision on the above manuscript.

To see the reviewers' reports and a copy of this decision letter, please go to: https://submit-jcs.biologists.org and click on the 'Manuscripts with Decisions' queue in the Author Area. (Corresponding author only has access to reviews.)

As you will see, the reviewers gave favourable reports but raised some critical points that will require amendments to your manuscript. I hope that you will be able to carry these out because I would like to be able to accept your paper, depending on further comments from reviewers.

Please ensure that you clearly highlight all changes made in the revised manuscript. Please avoid using 'Tracked changes' in Word files as these are lost in PDF conversion.

I should be grateful if you would also provide a point-by-point response detailing how you have dealt with the points raised by the reviewers in the 'Response to Reviewers' box. Please attend to all of the reviewers' comments. If you do not agree with any of their criticisms or suggestions please explain clearly why this is so.

© 2023. Published by The Company of Biologists under the terms of the Creative Commons Attribution License (https://creativecommons.org/licenses/by/4.0/).

Reviewer 1

Advance summary and potential significance to field

This study provides a comprehensive and unbiased inventory of the dynamic lipid rafts protein composition of resting and stimulated B lymphocytes. Authors employ a seven amino acid sequence that, when attached to an APEX2-mCherry construct, targets the fusion protein into lipid raft domains. This allows for biotinylation of adjacent proteins, which subsequently can be purified and identified by quantitative mass spectrometry. The power of this elegant approach is demonstrated by the verification of known signal effectors of the B cell antigen receptor (BCR), and furthermore, by the identification of hitherto unknown signaling molecules as well as their modification by sumoylation. Intriguingly, most of the BCR signal machinery appears to be excluded (rather than included) from lipid rafts upon BCR ligation. Finally, this solid approach is complemented by functional studies on the role of the newly identified signaling elements e.g. Golga3 and the impact of protein sumoylation on BCR signaling in cultured and primary B cells. Technically, the experiments were carefully planned and conducted with due diligence. Also, the manuscript is well written. Altogether, the methodology and the results will be of interest to a wide readership of cell biologists and immunologists alike.

Comments for the author

I have only a few comments.
- I appreciate the careful titration of superoxide, a necessary component of the APEX biotinylation mapproach that is, however, problematic as it is known to trigger B cell signaling by itself. Did authors also try shorter treatment periods (i.e. less than 1 min)? Are the phospho-protein patterns detected upon superoxide treatment (shown in Fig.1 and SFig.1) similar or even identical to those observed upon BCR stimulation of A20 cells?
- Given that sumoylation is confined to sites of BCR activation, I wonder whether BCR components themselves undergo this modification, e.g. Ig-alpha and/or Ig-beta. This could be easily tested, and if turned out to be the case, mutant BCRs that cannot be modified could be expressed and tested for their signaling capability. This would be very interesting, yet the complete set of these experiments would go beyond the scope of this manuscript. However, the information of BCR sumoylation would be nice to have.
- The BCR signal gate keeper SLP65 (BLNK) was found to be a component of the lipid rafts proteome. SLP65 is reported to exist in vesicle-associated phase-separated droplets as a result of its association with CIN85. I thus wonder whether CIN85 was also found in the proximity proteome, and whether the identified trafficking molecules might be involved in the functional communication between the BCR and SLP65/CIN85 droplets (i.e. in the recruitment of vesicle-associated SLP65 to sites of BCR activation).

Reviewer 2

Advance summary and potential significance to field

The manuscript by Awoniyi et al provides some insight into early events of B cell activation by addressing the protein composition within the lipid rafts upon BCR activation. The authors employ the enzyme ascorbate peroxidase (APEX2), which in cellulo generates short-lived biotin radicals, that in turn randomly bind to proteins in their vicinity (10-20 nm) within 1 min. APEX2 is furthermore fused with with an mCherry marker and the 7-amino acid sequence MGCVCSS, allowing its localization to lipid raft domains (Raft-APEX2). Using quantitative “proximity” proteomics, the authors provide a spatially and temporally resolved dynamic insight into the changes within the lipid raft identifying various proteins in the lipid rafts previously not associated with BCR signaling. I have reviewed this manuscript when it was first uploaded to Review Commons (Reviewer 3). The authors somewhat hesitantly addressed most of my concerns. I have nothing to add to these comments other than “newly generated data should be included in the manuscript”.
However, during the revision process the authors have shifted their focus to SUMO one of the hits in their APEX database, which definitely improved the manuscript.

© 2023. Published by The Company of Biologists under the terms of the Creative Commons Attribution License (https://creativecommons.org/licenses/by/4.0/).

They for the first-time link SUMOylation to early B cell activation in primary mouse B cells, more precisely to BCR signalling through the PI3K/AKT and MAPK/ERK axes. This is interesting but there are still some open questions and comments:

Line 119: if possible, please show that not shown data Line 146: if possible, please show that not shown data

Line 148 and Fig 1D and S1C: the quantification of three blots, which is provided with the comments to reviewer 3 should be included in the manuscript as the lanes in the Western blot presented in 1D remain different. Without the quantification the description of the blot is misleading Line 496: The lack of Syk should be mentioned (with data) in the Results section as I had specifically asked for it during the first review. Syk is among the major signalling elements downstream of the BCR. The authors provide a congruent response as to why they do not detect Syk in their APEX experiments. However, if they have any experimental evidence in this regard, it should be shown.

Comments for the author

Line 119: if possible, please show that not shown data Line 146: if possible, please show that not shown data Line 148 and Fig 1D and S1C: the quantification of three blots, which is provided with the comments to reviewer 3 should be included in the manuscript as the lanes in the Western blot presented in 1D remain different. Without the quantification the description of the blot is misleading Line 496: The lack of Syk should be mentioned (with data) in the Results section as I had specifically asked for it during the first review. Syk is among the major signalling elements downstream of the BCR. The authors provide a congruent response as to why they do not detect Syk in their APEX experiments. However, if they have any experimental evidence in this regard, it should be shown.

First revision

<u>Author response to reviewers' comments</u>

June 18th, 2023

To facilitate the review, we include here a point-by-point response to the reviewer's questions and comments.

Reviewer 1. *Advance Summary and Potential Significance to Field*:

This study provides a comprehensive and unbiased inventory of the dynamic lipid rafts protein composition of resting and stimulated B lymphocytes. Authors employ a seven amino acid sequence that, when attached to an APEX2-mCherry construct, targets the fusion protein into lipid raft domains. This allows for biotinylation of adjacent proteins, which subsequently can be purified and identified by quantitative mass spectrometry. The power of this elegant approach is demonstrated by the verification of known signal effectors of the B cell antigen receptor (BCR), and furthermore, by the identification of hitherto unknown signaling molecules as well as their modification by sumoylation. Intriguingly, most of the BCR signal machinery appears to be excluded (rather than included) from lipid rafts upon BCR ligation. Finally, this solid approach is complemented by functional studies on the role of the newly identified signaling elements e.g. Golga3 and the impact of protein sumoylation on BCR signaling in cultured and primary B cells. Technically, the experiments were carefully planned and conducted with due diligence. Also, the manuscript is well written. Altogether, the methodology and the results will be of interest to a wide readership of cell biologists and immunologists alike.

Ø We thank the Reviewer for their positive comments. We have now revised the manuscript to address the comments from both of the reviewers. We would like to note also that, at the same

© 2023. Published by The Company of Biologists under the terms of the Creative Commons Attribution License (https://creativecommons.org/licenses/by/4.0/).

time, we had to shorten the manuscript by ≈1000 words to fulfil the journal's requirements (modification sites linked to shortening of the manuscript are marked in green in the manuscript). The points added to address the reviewers' comments are marked in yellow in the manuscript.

Reviewer 1. *Comments for the Author*:

I have only a few comments. I appreciate the careful titration of superoxide, a necessary component of the APEX biotinylation mapproach that is, however, problematic as it is known to trigger B cell signaling by itself. Did authors also try shorter treatment periods (i.e. less than 1 min)? Are the phospho-protein patterns detected upon superoxide treatment (shown in Fig.1 and SFig.1) similar or even identical to those observed upon BCR stimulation of A20 cells?

Ø The effect of H_2O_2 on phosphorylation and potential activation of the BCR signaling proteins is an important point. When we started optimizing the APEX2-mediated biotinylation in B cells, we also tested 30-second H_2O_2-treatment and found the resulting biotinylation considerably lower than with 1-min treatment, based on flow cytometry detection of biotinylated proteins. This finding, together with normal tyrosine phosphorylation levels upon1 min of 1 mM H_2O_2-treatment, convinced us to stick with 1 min treatment to optimize the coverage of biotinylation and we did not continue with 30-second biotinylation for proteomic analysis. However, to better compare the proteins patterns undergoing tyrosine phosphorylation upon H_2O_2 and anti-IgM treatments, we have now added analysis using a H_2O_2 concentration 5 times higher than in our proteomics experiments, 5mM. This concentration triggers robust phosphorylation and, thus, allows for comparison of the upregulated phosphorylation patterns to the ones in anti-IgM activated cells. The data with a line plot analysis of new comparative Western blots is added in **Supplementary Fig S1I, J (for full Fig S1, see the end of this letter**; additionally, all 3 replicates are shown in the new Supplementary file S7 containing all uncropped immunoblots). The results indicate that some of the H_2O_2-phosphorylated proteins are likely the same than those triggered via the BCR, but at the same time, the general patterns of phosphorylation appear considerably different, indicating somewhat different types of responses.

Ø This data is now added in the text, in **lines 149-152**: "*We then asked to what extent the response to these high levels of H_2O_2 resembles BCR signaling and compared the tyrosine phosphorylation pattern induced by 5 mM H_2O_2 to IgM cross-linking. We detected some similarities but also profound differences between the two signals (Fig S1I,J).*" Finally, to put all the experiments on the H_2O_2 together, we conclude, in **lines 152-154**: "*Together, while it is possible that the 1 min treatment with 1 mM H_2O_2 facilitates some low-level phosphorylation, our data suggests that it has no significant effect to the BCR triggered phosphorylation events in our experimental conditions.*"

Given that sumoylation is confined to sites of BCR activation, I wonder whether BCR components themselves undergo this modification, e.g. Ig-alpha and/or Ig-beta. This could be easily tested, and if turned out to be the case, mutant BCRs that cannot be modified could be expressed and tested for their signaling capability. This would be very interesting, yet the complete set of these experiments would go beyond the scope of this manuscript. However, the information of BCR sumoylation would be nice to have.

Ø We thank the reviewer for this suggestion that is, indeed, very interesting. We have now carried out an immunoprecipitation experiment using anti-IgM antibodies and detecting the blot with anti-Sumo antibodies. We indeed can detect some SUMOylated bands in the IgM immunoprecipitates, but not of the expected size for Iga/b. Unfortunately, in the given time frame, we have not been able to optimize the protocol for consistency to be able to proceed for identification of the SUMOylated protein bands, for which we would ideally involve mass spectrometry. We agree with the reviewer that this is an interesting point for follow-up studies and will continue to work on this topic in the future.

The BCR signal gate keeper SLP65 (BLNK) was found to be a component of the lipid rafts proteome. SLP65 is reported to exist in vesicle-associated phase-separated droplets as a result of its

© 2023. Published by The Company of Biologists under the terms of the Creative Commons Attribution License (https://creativecommons.org/licenses/by/4.0/).

association with CIN85. I thus wonder whether CIN85 was also found in the proximity proteome, and whether the identified trafficking molecules might be involved in the functional communication between the BCR and SLP65/CIN85 droplets (i.e. in the recruitment of vesicle-associated SLP65 to sites of BCR activation).

Ø Indeed, in our dataset we identified high levels of SLP65/BLNK with significant downregulation in the raft environment at 5 and 10 min after BCR triggering (manuscript Supplementary file 3). Also CIN85 (SH3KBP1) was identified in the dataset but at lower levels and without significant dynamics. We noticed that CIN85 had been omitted from the heatmap Fig 5C, as it had not been annotated to the gene ontology (GO) classification "B cell activation" (GO:0042113). We have now curated the list of proteins and updated the heatmap correspondingly to also contain CIN85/SH3KBP1. As these two proteins do not really show similar behavior in our dataset and SLP65/BLNK shows downregulation it is difficult to speculate how this could be linked to its phase separation properties or vesicle association behavior. Various vesicle trafficking proteins are identified in the dataset, some of them with significant dynamicity and some without. With the limited information that we have about SLP65/CIN85 carriers, we cannot unfortunately postulate on the potential trafficking molecules or mechanisms.

Ø We have now briefly discussed this point in **lines 291-292**: "*The recently demonstrated phase-separation properties of BLNK could also be related to its strong localization to ordered lipid domains (Wong et al., 2020).*"

Reviewer 2. *Advance Summary and Potential Significance to Field*:

The manuscript by Awoniyi et al provides some insight into early events of B cell activation by addressing the protein composition within the lipid rafts upon BCR activation. The authors employ the enzyme ascorbate peroxidase (APEX2), which in cellulo generates short-lived biotin radicals, that in turn randomly bind to proteins in their vicinity (10-20 nm) within 1 min. APEX2 is furthermore fused with with an mCherry marker and the 7-amino acid sequence MGCVCSS, allowing its localization to lipid raft domains (Raft- APEX2). Using quantitative "proximity" proteomics, the authors provide a spatially and temporally resolved dynamic insight into the changes within the lipid raft identifying various proteins in the lipid rafts previously not associated with BCR signaling. I have reviewed this manuscript when it was first uploaded to Review Commons (Reviewer 3). The authors somewhat hesitantly addressed most of my concerns. I have nothing to add to these comments other than "newly generated data should be included in the manuscript". However, during the revision process the authors have shifted their focus to SUMO, one of the hits in their APEX database, which definitely improved the manuscript. They for the first-time link SUMOylation to early B cell activation in primary mouse B cells, more precisely to BCR signalling through the PI3K/AKT and MAPK/ERK axes. This is interesting but there are still some open questions and comments:

Ø We thank the Reviewer for their positive comments. We have now revised the manuscript to address the comments from both of the reviewers. We would like to note also that, at the same time, we had to shorten the manuscript by ≈1000 words to fulfil the journal's requirements (modification sites linked to shortening of the manuscript are marked in green in the manuscript). The points added to address the reviewers' comments are marked in yellow in the manuscript.

Line 119: if possible, please show that not shown data

Ø We have now added the data showing that our stable raft-APEX2 cell line is > 99% positive for both mCherry, marking for the APEX2 construct, and for IgM BCR (Supplementary Figure **S1A; for full Fig S1, see the end of this letter**). In the revised manuscript, this is referred to in **lines 115-116**.

Line 146: if possible, please show that not shown data

© 2023. Published by The Company of Biologists under the terms of the Creative Commons Attribution License (https://creativecommons.org/licenses/by/4.0/).

Ø We have now added the data showing normal BCR internalization kinetics in the stable raft-APEX2 cell line as compared to the parental A20 D1.3 to the manuscript (Supplementary Figure **S1D**; for full Fig S1, see the end of this letter). In the revised manuscript, this is referred to in **line 141.**

Line 148 and Fig 1D and S1C: the quantification of three blots, which is provided with the comments to reviewer 3 should be included in the manuscript as the lanes in the Western blot presented in 1D remain different. Without the quantification the description of the blot is misleading

Ø We have now also added the quantification to the manuscript (Supplementary Figure **S1F**; for full Fig S1, see the end of this letter) as well as show all the immunoblots in question in the new Supplementary file S7 containing all uncropped immunoblots. In the revised manuscript, this is referred to in **line 143**.

Line 496: The lack of Syk should be mentioned (with data) in the Results section, as I had specifically asked for it during the first review. Syk is among the major signalling elements downstream of the BCR. The authors provide a congruent response as to why they do not detect Syk in their APEX experiments. However, if they have any experimental evidence in this regard, it should be shown.

Ø We apologize for not addressing this point sufficiently in the previous version of the manuscript. We have now included the data showing that the raft-APEX2 cells, as compared to the parental A20 D1.3 cells, feature unaltered expression levels of Syk, as well as normal phosphorylation of Syk upon BCR triggering (Supplementary Figure **S2C-D**; **for full Fig S2, see the end of this letter**). Thus, unfortunately, the reason for not identifying Syk in the proteomic analysis remains unknown.

Ø These results are discussed, in the Results section, in **lines 270-275**: "*Notably, we did not detect the prominent BCR proximal kinase Syk in our dataset. To test for possible alterations in the expression levels of Syk in our cell line, we performed immunoblotting. We detected normal levels of both total Syk and Syk phosphorylation in raft-APEX2 cells as compared to the parental A20 cells (Fig. S2C,D), suggesting that the lack of identification of Syk in the dataset could result from inefficient biotinylation due to steric obstruction or a lack of biotinylation-suitable and available amino acid moieties on the protein surface.*" The data is shown in Supplementary Figure S2C, D.

© 2023. Published by The Company of Biologists under the terms of the Creative Commons Attribution License (https://creativecommons.org/licenses/by/4.0/).

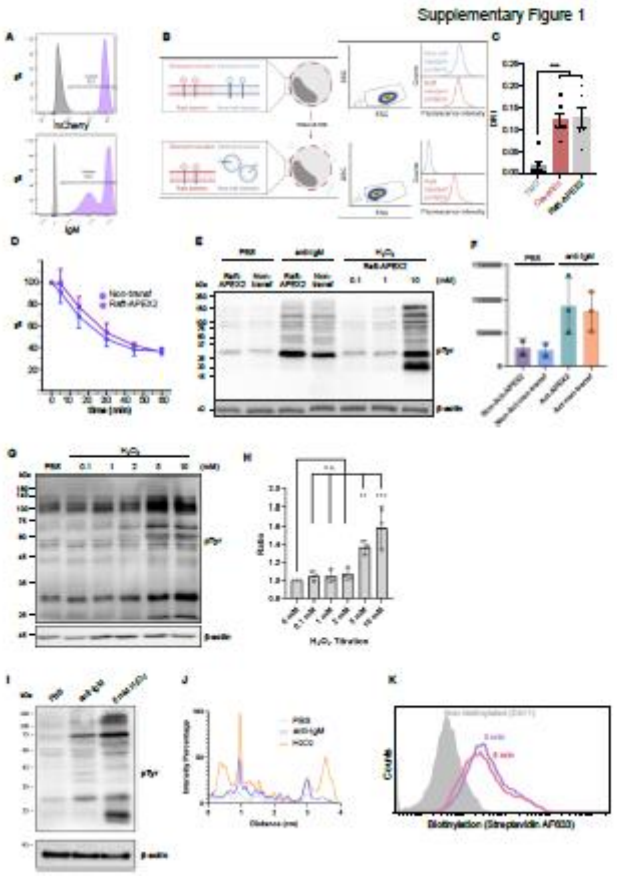

Supplementary Figure S1. Raft-APEX2 construct locates in the detergent-resistant membrane domains. Related to Fig. 1. **A)** Raft-APEX2 expressing A20 D1.3 cells used in the proteomic experiments were analysed for the expression levels of Raft-APEX2, by mCherry fluorescence (upper blot), and IgM BCR by fluorescently labelling the IgM (lower blot) using flow cytometry (n=3). **B)** A schematic representation of flow cytometry assay to detect lipid raft association of membrane proteins by analysis of detergent resistance. Upon treatment with TritonX-100 detergent, the proteins within lipid rafts are retained, whereas non-raft domains are dissolved. **C)** A20 D1.3 B cells were transfected with raft-APEX2, lipid raft marker (caveolin-1-RFP) or non- raft marker (TMD-GFP). The fluorescence of the markers was measured before, and after subjecting the samples to 0.1% Triton X-100 and the detergent resistance index was calculated as described in Materials and Methods (n = 6-8 independent experiments; unpaired t-test, *** < 0.001). **D)** BCR internalization assay by flow cytometry. A20 D1.3 cells transfected or not with Raft-APEX2 were stimulated with 10 µg/ml biotinylated anti- IgM $F(ab')_2$ fragments for 0, 5, 15, 30, 45, or 60 min, and the IgM remaining on the cell surface was detected with AF633-labelled streptavidin. Paired t-test, mean +/-SEM of three experiments. **E)** The original immunoblot that is shown rearranged in the main Figure 1 D and E. Raft-APEX2 expressing A20 D1.3 B cells and the parental A20 D1.3 (non-transfected) cells were treated with 0, 0.1, 1 and 10 mM H_2O_2, or 10 µg/ml anti-IgM $F(ab')_2$ fragments. Cells were lysed and subjected to Western blotting. The membranes were probed with HRP-anti phospho-Tyrosine antibodies and anti-b-actin as a loading control. **F)** Quantification of the pTyr-response of Raft-APEX2 expressing, or not, A20 D1.3 cells to anti-IgM $F(ab')_2$, from (E). T-test, mean +/-SEM of three experiments. **G)** An immunoblot like in (C), using Raft-APEX2 expressing A20 D1.3 B cells treated with 0, 0.1, 1, 2, 5 and 10 mM H_2O_2 for 1 min. **H)** Quantification of (G). One-way ANOVA, mean +/-SEM of three experiments. ** $p < 0.01$, *** $p < 0.001$. **I)** A pTyr-immunoblot of A20 D1.3 cells treated, or not, with 5 mM H_2O_2 for 1 min or 10 µg/ml anti-IgM $F(ab')_2$ for 10 min. **J)** Line plot comparison of the H_2O_2 and anti-IgM $F(ab')_2$ activated lanes in (I) (n=3, representative plot shown). **K)** Raft-APEX2 A20 D1.3 B cells were supplemented with biotin-phenol, activated (red line) or not with (violet line) $F(ab')_2$ fragments of anti-IgM antibodies for 5 min, and the biotinylation was

© 2023. Published by The Company of Biologists under the terms of the Creative Commons Attribution License (https://creativecommons.org/licenses/by/4.0/).

triggered or not (grey line) by adding 1 mM H_2O_2 for 1 min. Cells were fixed with 4% PFA, permeabilised, stained with AF633-labelled streptavidin and analyzed with flow cytometry. n=3, representative plot shown.

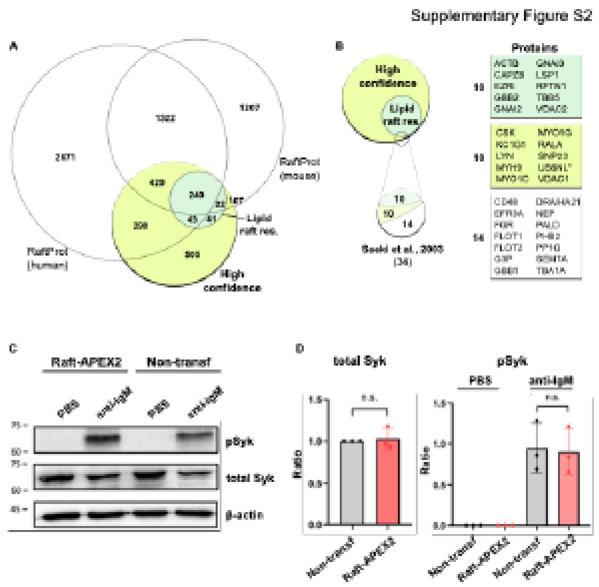

Supplementary Figure S2. Lipid raft-resident proteins in B cells. Related to Fig. 3. **A)** A Venn diagram showing the intersection between the data obtained in this study (Fig. 2C) to both mouse and human raft proteins in the RaftProt database. **B)** A Venn diagram showing the intersection between the data obtained in this study and the lipid raft proteins identified in Raji B cells by Saeki et al. (Saeki et al., 2003). **C)** A20 D1.3 cells transfected or not with Raft-APEX2 were stimulated, or not, with 10 µg/ml anti-IgM $F(ab')_2$ fragments for 10 min and immunoblotted for total Syk, phosphorylated Syk and b-actin for loading control (n=3). **D)** Quantification of (C). Paired t-test, data is shown as mean ± SEM, * $p < 0.05$.

<u>Second decision letter</u>

MS ID#: JOCES/2023/261119

MS TITLE: B cell receptor-induced protein dynamics and the emerging role of SUMOylation revealed by proximity proteomics

AUTHORS: Luqman O. Awoniyi, Diogo M. Cunha, Alexey V. Sarapulov, Sara Hernández-Pérez, Marika Runsala, Blanca Tejeda-González, Vid Šuštar, M. Özge Balci, Petar Petrov and Pieta K .Mattila
ARTICLE TYPE: Research Article

Thank you for sending your manuscript to Journal of Cell Science through Review Commons.

I am happy to tell you that your manuscript has been accepted for publication in Journal of Cell Science, pending standard ethics checks.

© 2023. Published by The Company of Biologists under the terms of the Creative Commons Attribution License (https://creativecommons.org/licenses/by/4.0/).

<u>Reviewer 1</u>

Advance summary and potential significance to field

Manuscript has further improved. All my comments and concerns were satisfactorily addressed. This is a solid piece of work which merits publication in Journal of Cell Science.

Comments for the author

Manuscript has further improved. All my comments and concerns were satisfactorily addressed. This is a solid piece of work which merits publication in Journal of Cell Science.

<u>Reviewer 2</u>

Advance summary and potential significance to field

The authors addressed all the concerns i had. best

Comments for the author

no comments . all concerns were addressed

© 2023. Published by The Company of Biologists under the terms of the Creative Commons Attribution License (https://creativecommons.org/licenses/by/4.0/).
